# Identification of a SARS-like bat coronavirus that shares structural features with the spike glycoprotein receptor-binding domain of SARS-CoV-2

**DOI:** 10.1099/acmi.0.000166

**Published:** 2020-09-08

**Authors:** Conchita Fraguas Bringas, David Booth

**Affiliations:** ^1^​ School of Life Sciences, University of Dundee, Nethergate, DD1 4HN, Scotland, UK

**Keywords:** angiotensin-converting enzyme 2, COVID-19, SARS-CoV-2, SARS coronavirus, SARS-like bat coronavirus, spike glycoprotein

## Abstract

SARS-CoV-2 is a recently emerged coronavirus that binds angiotensin-converting enzyme 2 (ACE2) for cell entry via its receptor-binding domain (RBD) on a surface-expressed spike glycoprotein. Studies show that despite its similarities to severe acute respiratory syndrome (SARS) coronavirus, there are critical differences in key RBD residues when compared to SARS-CoV-2. Here we present a short *in silico* study, showing that SARS-like bat coronavirus Rs3367 shares a high conservation with SARS-CoV-2 in important RBD residues for ACE2 binding: SARS-CoV-2’s Phe486, Thr500, Asn501 and Tyr505; implicated in receptor-binding strength and host-range determination. These features were not shared with other studied bat coronaviruses belonging to the *betacoronavirus* genus, including RaTG13, the closest reported bat coronavirus to SARS-CoV-2’s spike protein. Sequence and phylogeny analyses were followed by the computation of a reliable model of the RBD of SARS-like bat coronavirus Rs3367, which allowed structural insight of the conserved residues. Superimposition of this model on the SARS-CoV-2 ACE2-RBD complex revealed critical ACE2 contacts are also maintained. In addition, residue Asn488_Rs3367_ interacted with a previously defined pocket on ACE2 composed of Tyr41, Lys353 and Asp355. When compared to available SARS-CoV-2 crystal structure data, Asn501_SARS-CoV-2_ showed a different interaction with the ACE2 pocket. Taken together, this study offers molecular insights on RBD-receptor interactions with implications for vaccine design.

## Introduction

Since the first reports of pneumonia-like symptoms in December 2019 in the province of Hubei, PR China, the causative agent of COVID-19 disease was identified as severe acute respiratory syndrome (SARS) coronavirus 2 (SARS-CoV-2). The virus has rapidly spread worldwide, and has been declared a global health pandemic by the World Health Organization in March 2020 [1]. As of 8 August 2020, there have been 19 187 943 laboratory-confirmed cases of COVID-19 and 716 075 reported deaths [2]. However, many of its molecular mechanisms, although under rapid investigation, are yet to be elucidated.

SARS-CoV-2 is an enveloped, positive-sense RNA *betacoronavirus*, which belongs to the *sarbecovirus* subgenus. In order to gain cell entry, it requires a glycoprotein known as spike (S), which is expressed on its surface. The S protein is known to trigger host immune responses [3] and is the target of neutralizing antibodies[4, 5] and a focus of vaccine design.

S is a glycosylated homotrimer, composed of a large ectodomain, a single transmembrane anchor and a C-terminal intracellular tail [3]. The ectodomain encompasses two subunits, known as S1 and S2. The S1 subunit is responsible for receptor binding, with its C-terminal domain being critical for this interaction [6], also known as the receptor-binding domain (RBD). This spike region has been defined as the most variable part of SARS-CoV-2’s genome [4]. To engage with the host-cell receptor, the S1 subunit undergoes a number of conformational changes, where solved ectodomain cryo-EM structures have shown SARS-CoV-2’s spike can adopt both ‘open’ and ‘closed’ conformations [7, 8]. Upon receptor binding, the S1 subunit dissociates, and the S2 subunit, which contains the membrane fusion machinery, is responsible for mediating this process, which leads to viral entry into host cells [3].

SARS-CoV-2 shares many similarities with SARS-CoV [9], where they both use angiotensin-converting enzyme 2 (ACE2) as a host-cell receptor [4] and share conserved glycosylated sites on the S protein [7]. Moreover, it has been shown that alike SARS-CoV, SARS-CoV-2’s spike is primed by host-cell protease TMPRSS2 [10]. Despite their high sequence identity [4], SARS-CoV-2’s RBD differs in key residues [11], a region which has been identified as critical for ACE2 engagement [12]. These residues in SARS-CoV-2 correspond to Leu455, Phe486, Gln493, Asn501 and Tyr505, with four out of five residues differing between SARS-CoV and SARS-CoV-2 [4], thought to alter the binding affinity to ACE2 [13], with described binding affinities of SARS-CoV-2’s RBD to ACE2 being higher compared to that of SARS-CoV [8, 14].

Recent reports have shown evidence of cross-reactivity in antibodies that target the spike of SARS-CoV and SARS-CoV-2 [15, 16]. While other studies concluded that potent neutralizing antibodies of SARS-CoV such as m396 and CR3014, which target the ACE2-binding site did not bind SARS-CoV2, indicating the differences in the RBD can have an impact on antibody cross-reactivity [8, 17].

Pangolin CoV isolate MP789 is the closest reported coronavirus to SARS-CoV-2 in the RBD region [18], whereas bat CoV RaTG13 has the closest sequence to SARS-CoV-2 at both genome and spike-protein level [4], with confirmed ACE2 as the cell receptor [19].

Many horseshoe bats belonging to the *Rhinolophus* family are hosts of SARS-like (SL) coronaviruses, and they have been investigated in the context of the respiratory disease that first emerged in 2002–2003 [20], with some strains known to use ACE2 for cell entry [21–23]. Given the divergence in the molecular and structural spike-ACE2 interactions between SARS-CoV-2 and SARS-CoV, and bats being proposed as the original source of the virus [4], we set out to investigate other SL bat coronaviruses that may display sequence similarities in this variable albeit important region.

Bat SL coronavirus Rs3367 was first identified in March 2012 and isolated in Yunnan, PR China [22]. Its host is *Rhinolophus sinicus*, and when the Rs3367’s full genome was sequenced, it showed 99.9 % identity to WIV1 coronavirus, which has been shown to use ACE2 for cell entry [22]. Through a combination of sequence and structural modelling analyses, we show that this SARS-like bat CoV has a high conservation with SARS-CoV-2 in key ACE2-binding residues within the RBD region [12, 13], with four out of six amino acids conserved, in contrast with other studied coronaviruses, including bat CoV RaTG13, and other *alpha*- and *beta*-CoVs, that showed little conservation in these reported residues. Furthermore, we present *in silico* evidence for the maintenance of critical ACE2 contacts for viral cell entry, which have been involved in conferring spike-receptor-binding strength [24]. In addition, a key difference was identified in the interaction of Rs3367 and SARS-CoV-2 RBDs with a critical pocket on ACE2, a pocket previously defined as a hotspot for viral cell entry [25].

## Methods

### Sequence alignment

Sequence alignments of full-length spike proteins and for phylogeny analysis were performed in Clustal Omega [26] version 1.2.1. Percentage identity values were calculated from alignment data. All sequences were obtained from the National Centre for Biotechnology Information (NCBI) database [27], with recorded accession numbers in Tables 1 and S1 (available in the online version of this article).

**Table 1.** Percentage identity scores of full-length spike proteins of selected coronaviruses (CoVs) compared to that of SARS-CoV-2. Spike-protein sequences of HCoVs NL63 and OC43, MERS CoV, bat CoV RaTG13, SARS-CoV, SARS-CoV-2, SARS-like (SL) bat CoVs RsSHC014, Rs3367, ZC45 and bat CoVs Rm1 and Rp3 were aligned using Clustal Omega [26]. Percentage identity scores (%) were calculated from alignment data. Spike-protein sequences were retrieved from the NCBI [27] database, with used accession numbers shown

Spike protein	Accession no.	Percentage identity (%) to SARS-CoV-2
HCoV NL63	YP_003767.1	27.72
HCoV OC43	AAR01015.1	31.41
MERS CoV	YP_009047204.1	31.96
SARS CoV Tor2 isolate	YP_009825051.1	76.72
Bat SL CoV Rs3367	AGZ48818.1	77.70
Bat SL CoV RsSHC014	AGZ48806.1	77.94
Bat CoV RaTG13	QHR63300.2	97.56
Bat SL CoV ZC45	AVP78031.1	82.30
Bat SL CoV Rf4092	ATO98145.1	75.51
Bat SARS CoV Rm1	ABD75332.1	76.62
Bat SARS CoV Rp3	AAZ67052.1	77.10

Sequence alignments for structural analysis were performed in Jalview [28] using the T Coffee with defaults setting. The sequences of SARS-CoV Tor2 isolate (GenBank: JX163928.1) and SARS-like bat coronavirus Rs3367 (GenBank: KC881006.1) were aligned. This alignment was then used for model input in MODELLER [29].

### Phylogenetic analysis

Model selection and evolutionary analysis were performed in mega X [30, 31]. Model selection was conducted using mega’s Find Best-Fit substitution model setting [30, 31]. A maximum-likelihood tree was assembled using 43 spike protein sequences of selected coronaviruses (Fig. S1, Table S1), using the Whelan and Goldman frequency model [32]. Statistical support for nodes was assessed using 500 bootstrap replicates. Initial tree(s) for the heuristic search were obtained automatically by applying Neighbor-Join and BioNJ algorithms to a matrix of pairwise distances estimated using the JTT model, and then selecting the topology with superior log-likelihood value. A discrete Gamma distribution was used to model evolutionary rate differences among sites [five categories (+G, parameter=0.8911)]. There were a total of 1653 positions in the final dataset.

### ModBase protein modelling

UCSF’s ModBase web server [33] was used to model the RBD structure of Rs3367 SARS-like bat coronavirus based on known structural PDB data. Server input included a Jalview [28] sequence alignment file in fasta format of full-length Rs3367 (GenBank: KC881006.1) and SARS-CoV-2 (GenBank: MN908947.3) sequences. Model selection criteria was set to the best and longest scoring model and selected fold-assignment method was the Slow (Seq-Prf, PSI-Blast). The output was of two models, and the model with the highest sequence identity to the input sequence was chosen, with a value of 95 %. The structural data corresponding to the crystal structure of the spike-protein RBD from the 2002–2003 SARS coronavirus human strain was used as a template for the model [34] (PDB code 3D0G). Model quality criteria outputs classified the model as reliable [35, 36], with key parameters summarized in Table 2. The ModPipe version SVN.r1661 and MSALL method [35] was used for model creation.

**Table 2.** SARS-like bat coronavirus Rs3367 spike-protein RBD model output parameters. The spike-protein RBD was modelled using MODELLER [29], using known structural data [34] (PDB code 3D0G) as the template. Output parameters and model characteristics are summarized below

Sequence identity	95 %
**Protein length**	243
**Template PDB code**	3D0G
**Template region**	349–502
**E-value**	0
**GA341**	1
MPQS*	1.64604
**z-DOPE**	−0.09
**Predicted RMSD (Å**)	2.107
**Predicted native overlap** (**3.5 Å**)	0.969

*MPQS, ModPipe protein quality Score [35, 36].

### Model building of RBD of SARS-CoV-2 superimposed on Rs3367 SARS-like bat coronavirus RBD

The structure of the spike glycoprotein RBD of SARS-CoV-2 (PDB: 6LZG, chain B) [14] was superimposed on the computed model of Rs3367 SARS-like bat coronavirus RBD in Chimera [37] using MatchMaker [38] structural-alignment command tool.

## Results

### SARS-like bat coronavirus Rs3367 shares conserved structural features in the spike glycoprotein RBD

**Fig. 1.**
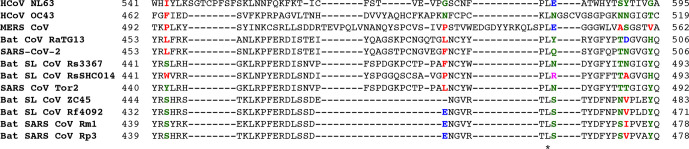
Sequence alignment of spike-protein region of HCoVs NL63 (541–595) and OC43 (462–519), MERS CoV (492–562), bat CoV RaTG13 (453–506), SARS-CoV-2 (453–506), SARS-CoV Tor2 isolate (440–492), SARS-like bat CoVs RsSHC014 (441–493), Rs3367 (441–493), ZC45 (444–483), Rf4092 (432–471), and bat SARS CoVs Rm1 (439–478) and Rp3 (439–478). A multiple sequence alignment was conducted in Clustal Omega [26]. Key residues critical for ACE2 binding [12, 13] are highlighted according to Clustal colouring. See Table 1 for selected accession numbers.

A multiple sequence alignment analysis of spike-protein sequences of selected *alpha-* and *betacoronaviruses* was conducted, focusing on the RBD region, which contains residues denoted as critical for human ACE2 receptor binding [12, 13]. The six residues correspond to Leu455, Phe486, Asn493, Thr500, Asn501 and Tyr505 in SARS-CoV-2. MERS-CoV and human coronavirus (HCoV) OC43 did not show any conservation in these residues, an expected result given both viruses are known to use a different receptor for cell entry [39, 40]. HCoV NL63, is able to use ACE2 for entry [41]. However, this *alphacoronavirus* did not have any conserved residues (Fig. 1).

Compared to SARS-CoV-2, the highest sequence identity of the spike-protein region corresponded to bat CoV RaTG13, with a value of 97.56 %, followed by SL bat CoVs ZC45, RsSHC014 and Rs3367, with corresponding identities of 82.30, 77.94 and 77.70 %; with the last two being slightly higher than in SARS-CoV, as previously reported [22] (Table 1). When investigating ACE2-binding residue conservation, Rs3367 had the most conserved residues compared to SARS-CoV-2, with four out of six conserved residues, as opposed to two in RaTG13 and one in ZC45 and RsSHC014 CoVs (Fig. 1). SARS-like bat coronavirus Rs3367’s conserved RBD residues are Phe473, Thr487, Asn488 and Tyr492, which correspond to Phe486, Thr500, Asn501 and Tyr505 in SARS-CoV-2.

**Fig. 2.**
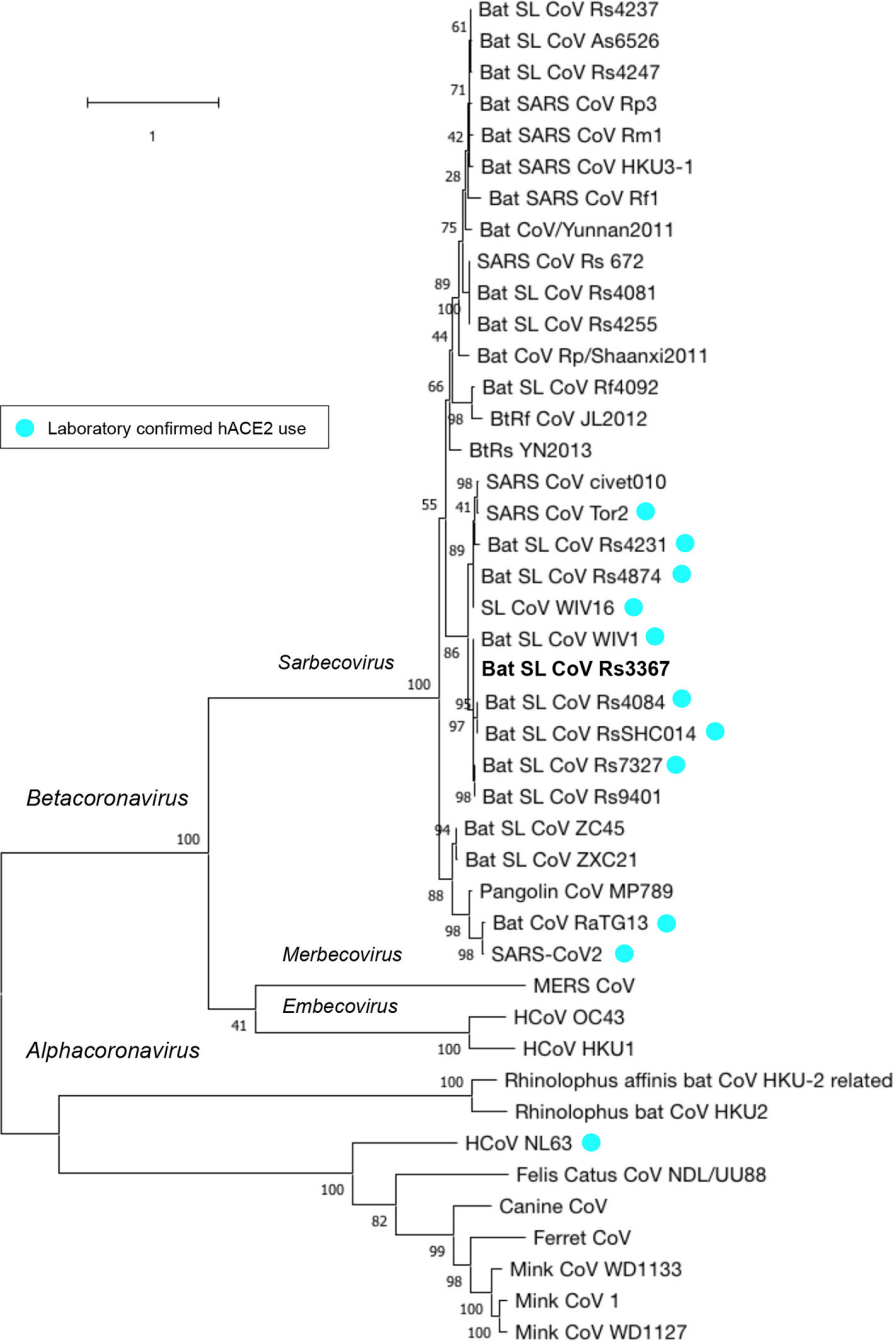
Phylogenetic tree based on the spike-protein sequences of CoVs and SARS-like CoVs. A maximum-likelihood tree was assembled in mega X [30, 31] using 43 spike-protein sequences of selected coronaviruses (Fig. S1,Table S1). The tree was inferred using the maximum-likelihood and Whelan and Goldman frequency model [32], with a discrete Gamma distribution to model evolutionary rate differences among sites [five categories (+*G*, parameter=0.8911)]. The tree with the highest log likelihood (−41046.54) is shown. The percentage of trees in which the associated taxa clustered together is shown next to the branches. The tree is drawn to scale, with branch lengths measured in the number of substitutions per site. Corresponding taxa names are indicated, with bat SARS-like CoV Rs3367 highlighted in bold and laboratory-confirmed ACE2-binding viruses indicated with cyan-coloured circles.

To place SARS-like bat CoV Rs3367 in an evolutionary context, we conducted a phylogenetic analysis focusing on the spike protein, including coronaviruses that both use and do not use ACE2 for cell entry (Fig. 2). Rs3367 clustered with other *betacoronaviruses* in the *sarbecovirus* subgenus, where SARS-CoV-2 and SARS-CoV also belong. Within the *sarbecovirus* branch, a highly supported node englobed bat SARS-like CoVs ZC45 and ZXC21, forming a distinct subclade from that containing pangolin CoV MP789, which branched off earlier than the subclade composed of SARS-CoV-2 and RaTG13, of 98 % statistical support (Fig. 2).

In line with previous reports[22], Rs3367’s spike sequence was closest to bat CoV WIV1 and bat SL CoV Rs4084, both known to use ACE2 [22, 42]. It also clustered with other bat SL CoVs that have been confirmed for ACE2 receptor usage, which included SARS-CoV but not SARS-CoV-2 (Fig. 2). Interestingly, Rs3367 did not cluster with another clade of SARS and SARS-like bat CoVs, which contained isolates that were reported as being incapable of ACE2 binding, such as bat SL CoV As6526 [23] and bat SARS CoV Rp3 [43].

**Fig. 3.**
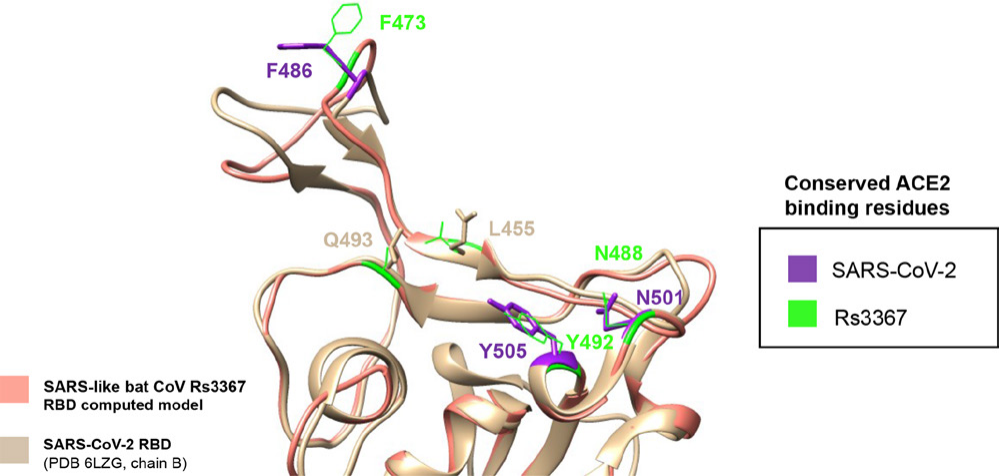
SARS-CoV-2 and SARS-like bat coronavirus Rs3367 spike-protein RBDs share key structural features. SARS-CoV-2 RBD (PDB 6LZG, chain B) [14] was superimposed on the computed RBD model for Rs3367 SARS-like bat coronavirus using the mmaker [38] structural alignment command in Chimera [37]. Key residues involved in ACE2 binding are highlighted in neon green (Rs3367) and purple (SARS-CoV-2).

Due to the observed high degree of conservation in the reported ACE2-binding residues between SARS-CoV-2 and bat SL CoV Rs3367, we investigated whether the global structural features of the spike’s RBD of this coronavirus also showed a conserved conformation when compared to SARS-CoV-2. Using a sequence alignment of SARS-like CoV Rs3367 and SARS-CoV as input, a structural model of the spike-protein RBD of this SARS-like bat strain was computed using ModBase web server [33], with known SARS-CoV structural data used as the template for modelling [34] (PDB: 3D0G). Output parameters classified the model as reliable [35, 36] (Table 2, Fig. 3).

The key conserved residues were observed and highlighted accordingly using Chimera [37], and the spike glycoprotein structure of SARS-CoV-2 [14] was superimposed on the modelled spike region from SARS-like CoV Rs3367 (Fig. 3). Modelling results revealed both RBD structures of the spikes have very similar three-dimensional structures, and indicate a conserved spatial conformation of SARS-CoV-2’s corresponding Phe486, Asn501 and Tyr505 residues.

The two other critical residues which are not conserved between SARS-CoV-2 and Rs3367 correspond to Gln493_SARS-CoV-2_, with an asparagine in Rs3367 (Asn480_Rs3367_) and residue Leu455_SARS-CoV-2_, with a serine present instead (Ser443_Rs3367_) (Fig. 3). The Gln-Asn change concerns two amino acids that both have polar uncharged side chains, whereas the Leu-Ser change involves a neutral non-polar amino acid changing to a neutral polar amino acid, pointing to a more significant residue difference. However, the contributions of these changes to spike-ACE2 interactions are yet to be investigated.

### Conserved ACE2-RBD interactions in SARS-CoV-2 and SARS-like Rs3367 CoV

**Fig. 4.**
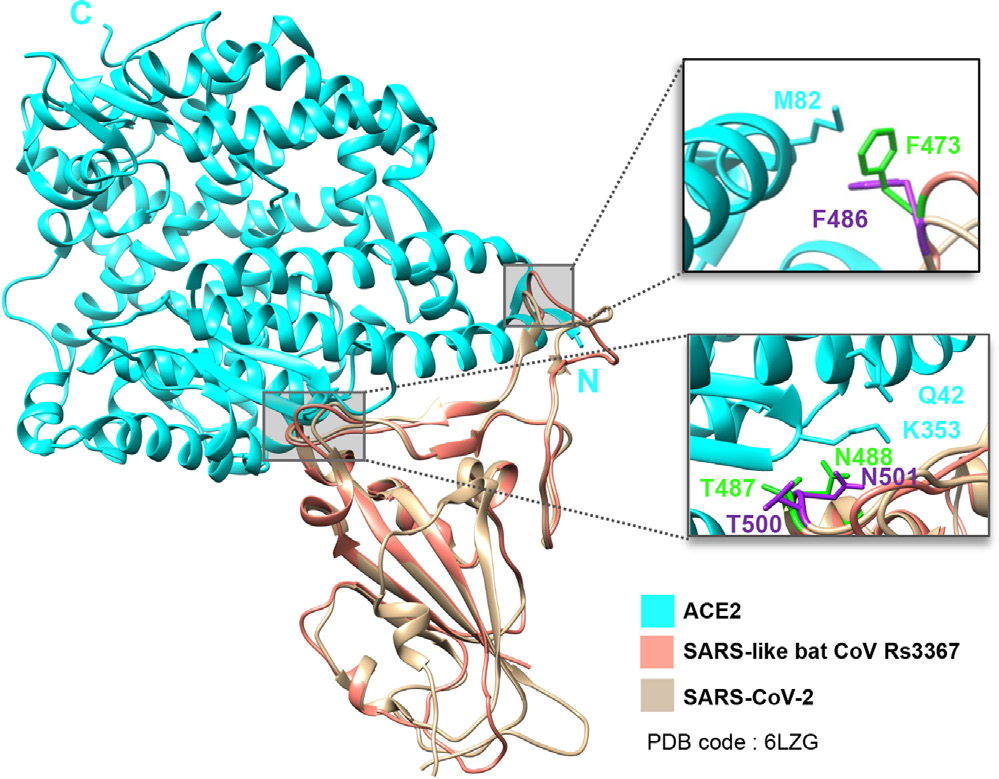
Conserved ACE2 and spike-protein RBD interactions in SARS-CoV-2 and SARS-like bat CoV Rs3367. The Rs3367 RBD model was superimposed on the structure of ACE2 receptor complexed with SARS-CoV-2 (PDB 6LZG) [14] in Chimera [37]. Conserved RBD residues are shown in neon green (Rs3367) and purple (SARS-CoV-2). ACE2 residues are shown in cyan.

**Fig. 5.**
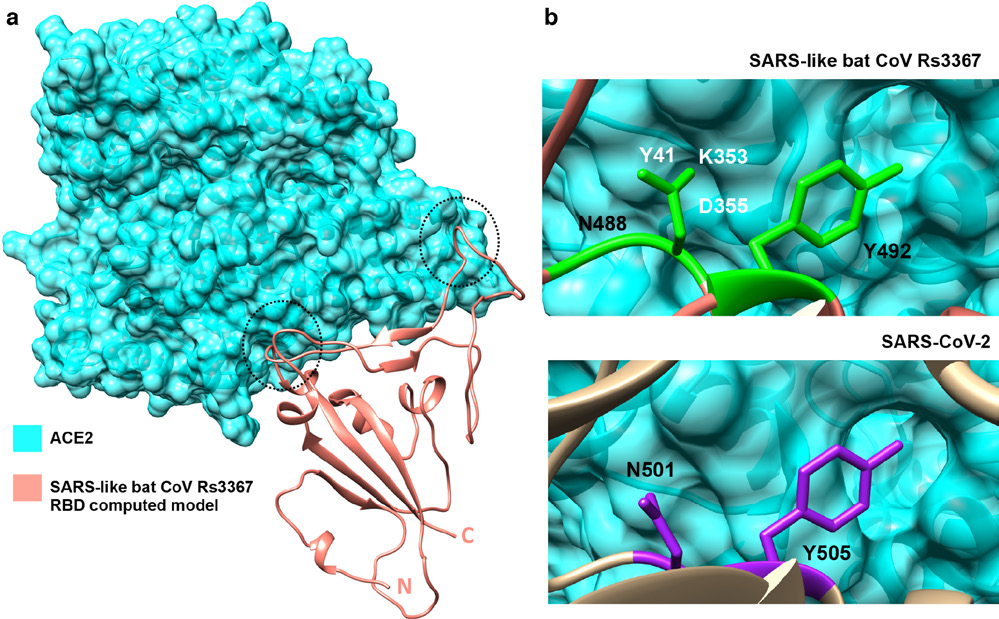
Predicted SARS-like bat CoV Rs3367 interactions with ACE2 receptor. (a) The surface structure of ACE2 (shown in cyan; PDB code: 6LZG, chain A) [14] complexed with the RBD model of SARS-like bat Rs3367 coronavirus (salmon) viewed in Chimera [37], with key contact areas shown with black circles. (b) ACE2 surface-RBD interactions of conserved Y505_SARS-_ CoV-2 and N501_SARS-CoV-2_ residues in SARS-like Rs3367 coronavirus isolate (top panel; shown in neon green) and in SARS-CoV-2 (bottom panel; shown in purple). White residues in the top panel represent the composition of the ACE2 pocket [24] interacting with N488.

We then investigated whether the high degree of conservation in the spike RBD between SARS-like bat CoV Rs3367 and SARS-CoV-2 translated to conserved ACE2-spike contacts. To test this, the modelled RBD of Rs3367 was superimposed on the solved structure of ACE2 complexed with SARS-CoV-2 RBD [14]. This analysis revealed that not only are there key residues conserved, but it suggests, that ACE2 residues Met82, Gln42 and Lys353 interactions with Phe486, Thr500 and Asn501 [13] are conserved in Rs3367, corresponding to Phe473, Thr487 and Asn488 (Fig. 4). To gain further detail on the receptor surface-RBD interactions between the spike proteins of Rs3367 and SARS-CoV-2, the solved structure of ACE2 complexed with SARS-CoV-2’s RBD (PDB: 6LZG) [14] was superimposed on the Rs3367 computed model in Chimera [37]. The Rs3367 model and ACE2 were then selected to be visualized alone (Fig. 5a).

This confirmed the conservation of two points of interaction of Rs3367 with ACE2 receptor, which have also been observed in SARS-CoV-2, corresponding to the atomic interactions of the spike RBD with Met82_ACE2_ near the N-terminus and with residues Gln42_ACE2_ and Lys353_ACE2_ farther away from the amino terminus.

Observing the surface-receptor conformation, two major pockets can be observed at those same locations (Fig. 5a). The pocket farther away from the N-terminus, which appears less exposed was further studied, given the involvement of two ACE2-RBD residue interactions instead of a single residue as in the case of Met82_ACE2_. *In silico* results showed the tyrosine-ACE2 pocket interactions, which take place in SARS-CoV-2 are conserved in Rs3367 (Fig. 5b, top panel), while in SARS-CoV-2 (Fig. 5b, bottom panel) Tyr505 residue conformation suggests a deeper interaction with the pocket, although structural refinement with additional data should confirm this.

Furthermore, residue Asn488_Rs3367_, which corresponds to Asn501_SARS-CoV-2_, interacts with a pocket composed of the previously reported Lys353 [13] and residues Asp355 and Tyr41 (pocket shown in Fig. 5b). Although structural data has confirmed SARS-CoV-2’s Asn501 residue forms a hydrogen bond with ACE2’s Tyr41 [44], we found that, compared to Asn488_Rs3367_, SARS-CoV-2’s asparagine residue points outward and does not accommodate as well into the Tyr41-Lys353-Asp355 pocket compared to Rs3367’s interaction with the receptor surface (Fig. 5b). This difference may result in a change in ACE2-binding strength between the two viruses, however, its significance and whether this should prove advantageous for cell entry is yet to be ascertained.

## Discussion

To date, the closest identified coronavirus to SARS-CoV-2 with bat origin is RaTG13 CoV, which shares a 96 % identity at whole-genome-sequence level and more than 93.1 % identity in the spike glycoprotein region [4]. Alongside other reported coronaviruses of the *sarbecovirus* subgenus such as SARS-CoV and SL CoV RsSHC014, RaTG13 has been shown to use receptor ACE2 for entry [19]. Despite its high sequence identity to SARS-CoV-2, RaTG13 and other studied CoVs showed little conservation in key ACE2-binding reported residues within the spike-protein RBD.

Although SARS-like bat coronavirus Rs3367 has a 77.70 % identity to SARS-CoV-2 in the spike sequence, it showed a high conservation in the studied ACE2-binding residues. This bat coronavirus shares a 99.9 % sequence identity with bat coronavirus WIV1, which was confirmed to use human, bat and civet ACE2 for cell entry [22]. Phylogeny spike-protein analysis revealed this strain clustered closely with other ACE2-using bat SL CoVs, where many shared the bat host *Rhinolophus sinicus* (Fig. 2).

Here, we reported using sequence data and through spike RBD structural modelling, a previously identified SARS-like bat coronavirus [22], which shares conserved structural features with SARS-CoV-2 in critical residues known from SARS-CoV studies to mediate ACE2-spike binding interactions [13, 14].

A study looking at SARS-CoV-2’s RBD concluded its interactions with ACE2 are stronger than those between SARS-CoV and ACE2 [45], where researchers defined Phe486 as a key residue, which has the ability to reach into a deep hydrophobic pocket in ACE2, and has a major role in conferring binding strength to this receptor [45]. Here we have shown that not only is this key residue conserved in Rs3367 at a sequence level, but its three-dimensional conformation also points to a conservation in its interaction with Met82_ACE2_, alongside conserved interactions with other key ACE2 residues, which include Gln42 and Lys353 as shown by the *in silico* structural studies of superimposing the RBDs of Rs3367 and SARS-CoV-2 (Fig. 4).

Surface ACE2 analyses with the RBDs of SARS-CoV-2 and Rs3367 have shown another pocket composed of Lys353, Asp355 and Tyr41 is important in the receptor-binding interactions of both Rs3367 and SARS-CoV-2, views which have been confirmed in SARS-CoV-2 by x-ray crystallography data [44]. We found that SARS-like bat CoV Rs3367 has an interaction with a critical ACE2 pocket, which differs from that of SARS-CoV-2 (Fig. 5b). This pocket has been previously defined as a viral hotspot for ACE2 interaction, where a study conducted in 2011 concluded its structure confers important energy contributions to ACE2-viral RBD interactions in SARS-CoV and NL63-CoV [46]. Tyr41 corresponds to a histidine residue (His41) in the ACE2 receptor of several studied bat species [47], and has been proposed to be responsible for the weak binding of human SARS-CoV, where mutation of this residue to a tyrosine greatly increases receptor activity [47], implicating this pocket in human infectivity. Furthermore, when Asn501_SARS-CoV-2_ was mutated to a threonine, this significantly reduced ACE2-binding affinity [19], indicating the importance of this residue in receptor binding. This residue is also conserved in SL bat CoV Rs3367 and together with the presented *in silico* data, feature important ACE2-RBD interactions, which may have implications in the context of vaccine design.

### Study limitations

It is important to note that there are limitations to the MatchMaker [38] alignment command used to superimpose and compare RBDs from SARS-CoV-2 and Rs3367 strains. The *in silico* data shown, although computed behind a reliable model (Table 2), is a representation of the plausible structural conformations that can ensue. Future biochemical data will allow gaining further insight into the nature of these molecular interactions between ACE2 and the spike viral protein of Rs3367.

## Supplementary Data

Supplementary material 1
