# Supplementary material for: Identification of a SARS-like bat coronavirus that shares structural features with the spike glycoprotein receptor-binding domain of SARS-CoV-2
Source: Access Microbiol. 2020 Sep 8;2(11):acmi000166. doi: 10.1099/acmi.0.000166 (PMC7717483; doi:10.1099/acmi.0.000166)
Supplement: Supplementary material 1 [file acmi-2-166-s001.pdf]

**Table S1.** Accession numbers of spike protein sequences of viral strains used for conducted sequence alignments. All sequences were retrieved from the National Center for Biotechnology Information (NCBI) database [27].

| **Spike protein** | **Accession no.** |
|---|---|
| Mink coronavirus 1 | QKX95789.1 |
| Mink coronavirus strain WD1127 | YP_009019182.1 |
| Mink coronavirus strain WD1133 | ADI80523.1 |
| Bat SARS-like coronavirus ZC45 | AVP78031.1 |
| Bat SARS-like coronavirus ZXC21 | AVP78042.1 |
| Bat SARS-like coronavirus Rs4081 | ATO98120.1 |
| Bat SARS-like coronavirus Rs4084 | ATO98132.1 |
| Bat SARS-like coronavirus Rs4231 | ATO98157.1 |
| Bat SARS-like coronavirus Rs4237 | ATO98169.1 |
| Bat SARS-like coronavirus Rs4247 | ATO98181.1 |
| Bat SARS-like coronavirus Rs4255 | ATO98193.1 |
| Bat SARS-like coronavirus Rs4874 | ATO98205.1 |
| Bat SARS-like coronavirus Rs7327 | ATO98218.1 |
| Bat SARS-like coronavirus Rs9401 | ATO98231.1 |
| Bat SARS-like coronavirus Rf4092 | ATO98145.1 |
| Bat SARS-like coronavirus As6526 | ATO98108.1 |
| SARS-like coronavirus WIV16 | ALK02457.1 |
| Bat SARS-like coronavirus WIV1 | AGZ48831.1 |
| Pangolin coronavirus MP789 | QIG55945.1 |
| Bat SARS-like coronavirus RsSHC014 | AGZ48806.1 |
| Bat SARS-like coronavirus Rs3367 | AGZ48818.1 |
| SARS coronavirus Rs_672/2006 | ACU31032.1 |
| BtRs-BetaCoV/YN2013 | AIA62330.1 |
| Bat coronavirus Rp/Shaanxi2011 | AGC74165.1 |
| Bat coronavirus Cp/Yunnan2011 | AGC74176.1 |
| Bat SARS CoV Rf1/2004 | ABD75323.1 |
| Bat SARS CoV Rm1/2004 | ABD75332.1 |

| | |
|---|---|
| Bat SARS CoV Rp3/2004 | AAZ67052.1 |
| Bat SARS coronavirus HKU3-1 | AAY88866.1 |
| BtRf-BetaCoV/JL2012 | AIA62277.1 |
| Human coronavirus NL63 | YP_003767.1 |
| Human coronavirus OC43 | AAR01015.1 |
| Middle East respiratory syndrome-related coronavirus (MERS-CoV) | YP_009047204.1 |
| Human coronavirus HKU1 | YP_173238.1 |
| SARS coronavirus civet010 | AAU04649.1 |
| Canine coronavirus | ADB28907.1 |
| Feline coronavirus | AGT52080.1 |
| Ferret coronavirus | YP_009256197.1 |
| Bat coronavirus RaTG13 | QHR63300.2 |
| Rhinolophus affinis bat coronavirus HKU2-related | QHA24703.1 |
| Rhinolophus bat coronavirus HKU2 | YP_001552236.1 |
| Severe acute respiratory syndrome coronavirus (SARS) Tor2 isolate | YP_009825051.1 |
| Severe acute respiratory syndrome coronavirus 2 (SARS-CoV-2) | YP_009724390.1 |

**Figure S1. Multiple sequence alignment of spike proteins from selected *alpha*- and *betacoronaviruses*.** A multiple sequence alignment was conducted using Clustal Omega [26] version 1.2.1. Used accession numbers recorded in Table S1.

```
HCoV_NL63/1-1356                                  MKLFLILLVLPL-----------ASCFFTCNS-----------------NANLSML---- 28

Felis_Catus_CoV_NDL/UU88/1-1467                   MILLILALV-------S---T-----VTSEDA-----------------PHGVTLPQFNT 28

Ferret_CoV/1-1438                                 MFEMRTPVFLTVCIVLL---QYAHCDTFNCST-----------------LHNTTLWATDQ 40

Canine_CoV/1-1457                                 MKFLVFVV-------IV---PLIYGDEFPCSK-----------------FINRTIG--NH 31

Mink_CoV_WD1133/1-1429                            MRKMLLILVLIPC-VL--------GDLFPCRT-----------------SINSTIG--NH 32

Mink_CoV_1/1-1439                                 MR--LLVVAFLIVAVNC---DDIANLTSRCGV-----------------LKNESIG--SH 36

Mink_CoV_WD1127/1-1438                            MFTKLLLLAVVSVAVKC---DDIRNLTARCAT-----------------LQNVSIG--SH 38

Rhinolophus_affinis_bat_CoV_HKU-2_related/1-1119  ------------------------------------------------------------ 0

Rhinolophus_bat_CoV_HKU2/1-1128                   ------------------------------------------------------------ 0
```

| | | |
|---|---|---|
| HCoV_OC43/1-1361 | MFLILLISL--PTA-------FAVIGDLKCTSDTSYINDKDTGPPPISTDTVDVTNGLGT | 51 |
| HCoV_HKU1/1-1356 | MLLI-IFIL--PTT-------LAVIGDFNCTNFA--INDLNTTVPRISEYVVDVSYGLGT | 48 |
| MERS_CoV/1-1353 | MIHSVFLLMFLLTPTESYVDVGPDSVKSACIEVDIQQTFFDKTWP---RP-IDVSKADGI | 56 |
| Bat_SL_CoV_ZC45/1-1246 | MLFFLF--------------LQFALVNSQCVN------LTGRTPL---NPNYTNSSQRGV | 37 |
| Bat_SL_CoV_ZXC21/1-1245 | MLFFLF--------------LQFALVNSQCDL-------TGRTPL---NPNYTNSSQRGV | 36 |
| Pangolin_CoV_MP789/1-1265 | MLFFFF--------------LHFALVNSQCVN------LTGRAAI---QPSFTNSSQRGV | 37 |
| Bat_CoV_RaTG13/1-1269 | -MFVFL--------------VLLPLVSSQCVN------LTTRTQL---PPAYTNSSTRGV | 36 |
| SARS-CoV2/1-1273 | -MFVFL--------------VLLPLVSSQCVN------LTTRTQL---PPAYTNSFTRGV | 36 |
| Bat_SL_CoV_Rs4084/1-1256 | MKLLVLVFA----------TLVSSYTIEKCLD------FDDRTPP---ANTQFLSSHRGV | 41 |
| Bat_SL_CoV_RsSHC014/1-1256 | MKLLVLVFA----------TLVSSYTIEKCLD------FDDRTPP---ANTQFLSSHRGV | 41 |
| Bat_SL_CoV_Rs7327/1-1256 | MKLLVLVFA----------TLVSSYTIEKCLD------FDDRTPP---ANTQFLSSHRGV | 41 |
| Bat_SL_CoV_Rs9401/1-1256 | MKLLVLVFA----------TLVSSYTIEKCLD------FDDRTPP---ANTQFLSSHRGV | 41 |
| Bat_SL_CoV_WIV1/1-1256 | MKLLVLVFA----------TLVSSYTIEKCLD------FDDRTPP---ANTQFLSSHRGV | 41 |
| Bat_SL_CoV_Rs3367/1-1256 | MKLLVLVFA----------TLVSSYTIEKCLD------FDDRTPP---ANTQFLSSHRGV | 41 |
| Bat_SL_CoV_Rs4231/1-1255 | -MFIFLFFL----------TLTSGSDLESCTT------FDDVQAP---NYPQHSSSRRGV | 40 |
| Bat_SL_CoV_Rs4874/1-1255 | -MFIFLFFL----------TLTSGSDLESCTT------FDDVQAP---NYPQHSSSRRGV | 40 |
| SL_CoV_WIV16/1-1255 | -MFIFLFFL----------TLTSGSDLESCTT------FDDVQAP---NYPQHSSSRRGV | 40 |
| SARS_CoV_civet010/1-1255 | -MFIFLLFL----------TLTSGSDLDRCTT------FDDVQAP---NYTQHTSSMRGV | 40 |
| SARS_CoV_Tor2/1-1255 | -MFIFLLFL----------TLTSGSDLDRCTT------FDDVQAP---NYTQHTSSMRGV | 40 |
| Bat_SL_CoV__Rf4092/1-1234 | --MFFLIGY---TA-----FLIGYTAATTCV--------TGPTTE---NKLNISSGSRGV | 39 |
| BtRf_CoV_JL2012/1-1236 | MFIVFLISY---TT-----FLIPYTATTTCF--------KGPTTE---NKLNISSGSRGV | 41 |
| BtRs_YN2013/1-1233 | ML---ILFV-----------FLPFIAADTCLN------FTNLAAP---AYNIASSSRRGV | 37 |
| Bat_CoV_Rp/Shaanxi2011/1-1240 | --MILLLLF-----------LSSAKAQEGCGV------ISNKPQR---TFDQYSSTFRGV | 38 |
| Bat_SARS_CoV_Rf1/2004/1-1241 | MKILIFAFL-----------VTLVKAQEGCGV------INLRTQP---KLTQVSSSRRGV | 40 |
| Bat_CoV/Yunnan2011/1-1241 | MKIFLFSLL-----------FSAALAQEGCGL------LSFKPQP---KLAQFSSSKRGV | 40 |
| SARS_CoV_Rs_672/2006/1-1241 | MKVLIVLLC-----------LGLVTAQDGCGH------ISTKPQP---LMDKFSSSRRGV | 40 |
| Bat_SL_CoV_Rs4081/1-1241 | MKVLIVLLS-----------LGLVTAQDGCGH------ISTKPQP---LMDKFSSSRRGV | 40 |
| Bat_SL_CoV_Rs4255/1-1241 | MKVLIVLLC-----------LGLVTAQDGCGH------ISTKPQP---LMDKFSSSRRGV | 40 |
| Bat_SARS_CoV_Rm1/2004/1-1241 | MKVLIFALL-----------FSLAKAQEGCGI------ISRKPQP---KMEKVSSSRRGV | 40 |
| Bat_SARS_CoV_HKU3-1/1-1242 | MKILIFAFL-----------ANLAKAQEGCGI------ISRKPQP---KMAQVSSSRRGV | 40 |
| Bat_SARS_CoV_Rp3/1-1241 | MKILILAFL-----------ASLAKAQEGCGI------ISRKPQP---KMAQVSSSRRGV | 40 |
| Bat_SL_CoV_As6526/1-1241 | MKILIFAFL-----------VTLVKAQEGCGI------ISRKPQP---KMAQVSSSRRGV | 40 |
| Bat_SL_CoV_Rs4237/1-1241 | MKILIFAFL-----------VTLVKAQEGCGL------ISRKPQP---KMAQVSSSRRGV | 40 |
| Bat_SL_CoV_Rs4247/1-1242 | MKILIFAFL-----------VTLVKAQEGCGI------ISRKPQP---KMAQVSSSRRGV | 40 |

| | | |
|---|---|---|
| HCoV_NL63/1-1356 | ----------------QLGVPDNSSTIVTGLLPTHWFCANQSTSVYSANGFFYIDVGNHR | 72 |
| Felis_Catus_CoV_NDL/UU88/1-1467 | SYNNAKFELNFYNFLQTWDIPPGTETILGGYLPYCGDG---------------------- | 66 |
| Ferret_CoV/1-1438 | TRLLDYFIANY-----SSRLPTGASVVLGDYFPTL------------------------- | 70 |
| Canine_CoV/1-1457 | WNLIETFLLNY-----SSRLPPNSDVVLGDYFPTV------------------------- | 61 |

| | | |
|---|---|---|
| Mink_CoV_WD1133/1-1429 | WYLIDNFVKNY-----SVSLPSNSDVVLGDYFPTV------------------------- | 62 |
| Mink_CoV_1/1-1439 | WYLIDNFVKNY-----STRLPPNSDVVLGDYFPTV------------------------- | 66 |
| Mink_CoV_WD1127/1-1438 | WYLIDNFVKNY-----SSRLPSNSDVVLGDYFPTV------------------------- | 68 |
| Rhinolophus_affinis_bat_CoV_HKU-2_related/1-1119 | ------------------------------------------------------------ | 0 |
| Rhinolophus_bat_CoV_HKU2/1-1128 | ------------------------------------------------------------ | 0 |
| HCoV_OC43/1-1361 | YYVLDRVYLNTTLFLNGYYPTSGSTYR--N-MALKGSV---------------------- | 86 |
| HCoV_HKU1/1-1356 | YYILDRVYLNTTILFTGYFPKSGANFR--D-LSLKGTT---------------------- | 83 |
| MERS_CoV/1-1353 | IYPQGRTYSNITITYQGLFPYQGDHGDMYVYSAGHA------------------------ | 92 |
| Bat_SL_CoV_ZC45/1-1246 | YYPDTIYRSDTLVLSQGYFLPFYSNV--SWYYSLTTNN---------------------- | 73 |
| Bat_SL_CoV_ZXC21/1-1245 | YYPDTIYRSDTLVLSQGYFLPFYSNV--SWYYSLTTNN---------------------- | 72 |
| Pangolin_CoV_MP789/1-1265 | YYPDTIFRSNTLVLSQGYFLPFYSNV--SWYYALTKTN---------------------- | 73 |
| Bat_CoV_RaTG13/1-1269 | YYPDKVFRSSVLHLTQDLFLPFFSNV--TWFHAIHVSG---------------------- | 72 |
| SARS-CoV2/1-1273 | YYPDKVFRSSVLHSTQDLFLPFFSNV--TWFHAIHVSG---------------------- | 72 |
| Bat_SL_CoV_Rs4084/1-1256 | YYPDDIFRSNVLHLVQDHFLPFDSNV--TRFITFGLN----------------------- | 76 |
| Bat_SL_CoV_RsSHC014/1-1256 | YYPDDIFRSNVLHLVQDHFLPFDSNV--TRFITFGLN----------------------- | 76 |
| Bat_SL_CoV_Rs7327/1-1256 | YYPDDIFRSNVLHLVQDHFLPFDSNV--TRFITFGLN----------------------- | 76 |
| Bat_SL_CoV_Rs9401/1-1256 | YYPDDIFRSNVLHLVQDHFLPFDSNV--TRFITFGLN----------------------- | 76 |
| Bat_SL_CoV_WIV1/1-1256 | YYPDDIFRSNVLHLVQDHFLPFDSNV--TRFITFGLN----------------------- | 76 |
| Bat_SL_CoV_Rs3367/1-1256 | YYPDDIFRSNVLHLVQDHFLPFDSNV--TRFITFGLN----------------------- | 76 |
| Bat_SL_CoV_Rs4231/1-1255 | YYPDEIFRSDTLYLTQDLFLPFYSNV--TGFHTINHR----------------------- | 75 |
| Bat_SL_CoV_Rs4874/1-1255 | YYPDEIFRSDTLYLTQDLFLPFYSNV--TGFHTINHR----------------------- | 75 |
| SL_CoV_WIV16/1-1255 | YYPDEIFRSDTLYLTQDLFLPFYSNV--TGFHTINHR----------------------- | 75 |
| SARS_CoV_civet010/1-1255 | YYPDEIFRSDTLYLTQDLFLPFYSNV--TGFHTINHT----------------------- | 75 |
| SARS_CoV_Tor2/1-1255 | YYPDEIFRSDTLYLTQDLFLPFYSNV--TGFHTINHT----------------------- | 75 |
| Bat_SL_CoV__Rf4092/1-1234 | YYPDDIFRSDVSVLVTGPFLRFNTTL--TWYNSWNQA----------------------- | 74 |
| BtRf_CoV_JL2012/1-1236 | YYPDDIFRSNVSVLVTGRFLRFNTTL--TWYNSWNQA----------------------- | 76 |
| BtRs_YN2013/1-1233 | YYPDDIFRSDFLHLVNDYFLPFGSNV--TQFFTQGTN----------------------- | 72 |
| Bat_CoV_Rp/Shaanxi2011/1-1240 | YYNDDIFRSDVLHLTQDYFLPFNTNV--TRYLSLNAAQ---------------------- | 74 |
| Bat_SARS_CoV_Rf1/2004/1-1241 | YYNDDIFRSDVLHLTQDYFLPFHSNL--TQYFSLNIES---------------------- | 76 |
| Bat_CoV/Yunnan2011/1-1241 | YYNDDIFRSDVLHLTQDYFLPFHSNL--TQYFSLNVDS---------------------- | 76 |
| SARS_CoV_Rs_672/2006/1-1241 | YYNDDIFRSDVLHLTQDYFLPFDTNL--TRYLSFNMDS---------------------- | 76 |
| Bat_SL_CoV_Rs4081/1-1241 | YYNDDIFRSDVLHLTQDYFLPFDTNL--TRYLSFNMDS---------------------- | 76 |
| Bat_SL_CoV_Rs4255/1-1241 | YYNDDIFRSDVLHLTQDYFLPFDTNL--TRYLSFNMDS---------------------- | 76 |
| Bat_SARS_CoV_Rm1/2004/1-1241 | YYNDDIFRSDVLHLTQDYFLPFDSNL--TQYFSLNIDS---------------------- | 76 |
| Bat_SARS_CoV_HKU3-1/1-1242 | YYNDDIFRSDVLHLTQDYFLPFDSNL--TQYFSLNVDS---------------------- | 76 |
| Bat_SARS_CoV_Rp3/1-1241 | YYNDDIFRSNVLHLTQDYFLPFDSNL--TQYFSLNVDS---------------------- | 76 |
| Bat_SL_CoV_As6526/1-1241 | YYNDDIFRSDVLHLTQDYFLPFDSNL--TQYFSLNVDS---------------------- | 76 |
| Bat_SL_CoV_Rs4237/1-1241 | YYNDDIFRSDVLHLTQDYFLPFDSNL--TQYFSLNVDS---------------------- | 76 |
| Bat_SL_CoV_Rs4247/1-1242 | YYNDDIFRSDVLHLTQDYFLPFDSNL--TQYFSLNVDS---------------------- | 76 |

| | | |
|---|---|---|
| HCoV_NL63/1-1356 | SAFALHTGYYDANQYYIYVTNEIGLNASV------------------------------- | 101 |
| Felis_Catus_CoV_NDL/UU88/1-1467 | ----------VNCGWYNFVFNTSVGSNA------KYSYINTQNLNIPNVHGVYFDVREH- | 109 |
| Ferret_CoV/1-1438 | ------------GPWYDCVSNTTYGGV--VLEDLRALYLDHQQG-----VARDLAFAVYG | 111 |
| Canine_CoV/1-1457 | ------------TPWFNCIRNNNNSLYV-TMENLKALYWDYATENITADHRQRLHVVVQG | 108 |
| Mink_CoV_WD1133/1-1429 | ------------EPWHNCLLEQTNSTV--IFDGLRALYWDYNNGQV-P-VRRYLAVAVYG | 106 |
| Mink_CoV_1/1-1439 | ------------NPWYNCMFRHWGERNALLLENIKALYWDTVQNNPGV-PNKYLLVTVYG | 113 |
| Mink_CoV_WD1127/1-1438 | ------------KPWYNCVFRYWGAHNALLLENLKALYWDTVQGVTGV-NNKYLLVSIHG | 115 |
| Rhinolophus_affinis_bat_CoV_HKU-2_related/1-1119 | ------------------------------------------------------------ | 0 |
| Rhinolophus_bat_CoV_HKU2/1-1128 | ------------------------------------------------------------ | 0 |
| HCoV_OC43/1-1361 | ---------LLSRLWFKPPFLSDF-------------------------INGIFAKVKNT | 112 |
| HCoV_HKU1/1-1356 | ---------YLSTLWYQKPFLSDF-------------------------NNGIFSRVKNT | 109 |
| MERS_CoV/1-1353 | -----------------TGTTPQK-------------------------LFVANYSQDVK | 110 |
| Bat_SL_CoV_ZC45/1-1246 | ----------AATKRTDNPILDFK-------------------------DGIYFAATEHS | 98 |
| Bat_SL_CoV_ZXC21/1-1245 | ----------AATKRTDNPILDFK-------------------------DGIYFAATEHS | 97 |
| Pangolin_CoV_MP789/1-1265 | ---------S-AEKRVDNPVLDFK-------------------------DGIYFAATEKS | 98 |
| Bat_CoV_RaTG13/1-1269 | ---------TNGIKRFDNPVLPFN-------------------------DGVYFASTEKS | 98 |
| SARS-CoV2/1-1273 | ---------TNGTKRFDNPVLPFN-------------------------DGVYFASTEKS | 98 |
| Bat_SL_CoV_Rs4084/1-1256 | ---------------FDNPIIPFR-------------------------DGIYFAATEKS | 96 |
| Bat_SL_CoV_RsSHC014/1-1256 | ---------------FDNPIIPFR-------------------------DGIYFAATEKS | 96 |
| Bat_SL_CoV_Rs7327/1-1256 | ---------------FDNPIIPFR-------------------------DGIYFAATEKS | 96 |
| Bat_SL_CoV_Rs9401/1-1256 | ---------------FDNPIIPFR-------------------------DGIYFAATEKS | 96 |
| Bat_SL_CoV_WIV1/1-1256 | ---------------FDNPIIPFK-------------------------DGIYFAATEKS | 96 |
| Bat_SL_CoV_Rs3367/1-1256 | ---------------FDNPIIPFK-------------------------DGIYFAATEKS | 96 |
| Bat_SL_CoV_Rs4231/1-1255 | ---------------FDNPVIPFK-------------------------DGVYFAATEKS | 95 |
| Bat_SL_CoV_Rs4874/1-1255 | ---------------FDNPVIPFK-------------------------DGVYFAATEKS | 95 |
| SL_CoV_WIV16/1-1255 | ---------------FDNPVIPFK-------------------------DGVYFAATEKS | 95 |
| SARS_CoV_civet010/1-1255 | ---------------FDNPVIPFK-------------------------DGIYFAATEKS | 95 |
| SARS_CoV_Tor2/1-1255 | ---------------FGNPVIPFK-------------------------DGIYFAATEKS | 95 |
| Bat_SL_CoV__Rf4092/1-1234 | ---------------YSSPILPFG-------------------------HGVYFSTIDKS | 94 |
| BtRf_CoV_JL2012/1-1236 | ---------------YSSPVLPFG-------------------------HGVYFSTIDKS | 96 |
| BtRs_YN2013/1-1233 | ---------------IDNPTLPFR-------------------------DGVYFAATEKS | 92 |
| Bat_CoV_Rp/Shaanxi2011/1-1240 | ----------NTIVYFDNHVIPFY-------------------------DGIYFAATERS | 99 |
| Bat_SARS_CoV_Rf1/2004/1-1241 | ----------DKIVYFDNPILKFG-------------------------DGVYFAATEKS | 101 |
| Bat_CoV/Yunnan2011/1-1241 | ----------DRQVYFDNPTLNFG-------------------------DGVYFAATEKS | 101 |
| SARS_CoV_Rs_672/2006/1-1241 | ----------ATKVYFDNPTLPFG-------------------------DGIYFAATEKS | 101 |
| Bat_SL_CoV_Rs4081/1-1241 | ----------ATKVYFDNPTLPFG-------------------------DGIYFAATEKS | 101 |
| Bat_SL_CoV_Rs4255/1-1241 | ----------ATKVYFDNPTLPFG-------------------------DGIYFAATEKS | 101 |
| Bat_SARS_CoV_Rm1/2004/1-1241 | ----------NKYTYFDNPILDFG-------------------------DGVYFAATEKS | 101 |
| Bat_SARS_CoV_HKU3-1/1-1242 | ----------DRYTYFDNPILDFG-------------------------DGVYFAATEKS | 101 |

| | | |
|---|---|---|
| Bat_SARS_CoV_Rp3/1-1241 | ----------DRFTYFDNPILDFG-------------------------DGVYFAATEKS | 101 |
| Bat_SL_CoV_As6526/1-1241 | ----------DRYTYFDNPILDFG-------------------------DGVYFAATEKS | 101 |
| Bat_SL_CoV_Rs4237/1-1241 | ----------DRYTYFDNPILDFG-------------------------DGVYFAATEKS | 101 |
| Bat_SL_CoV_Rs4247/1-1242 | ----------DRYTYFDNPILDFG-------------------------DGVYFAATEKS | 101 |

| | | |
|---|---|---|
| HCoV_NL63/1-1356 | -------------------------------------------------TLKICKFSRNT | 112 |
| Felis_Catus_CoV_NDL/UU88/1-1467 | --------NSDGIWDTRDRIGLLILVRGKSQYSLLMVLEDDVEANAPDVSVKICNWRHLH | 161 |
| Ferret_CoV/1-1438 | YPYTAIVVLNKELFNGQGYGGLLCFCNGNSTIPAF----------N-------ANCSTSC | 154 |
| Canine_CoV/1-1457 | NPYSITVTTTRDF-NSAEG-AIICICKGTPPKTTT----------VNSASQLNCNWGTEC | 156 |
| Mink_CoV_WD1133/1-1429 | DPYSVSVYFTLDY-EGPRG-AKICLCKGMPQPTHI--------------SGEANCWSSQC | 150 |
| Mink_CoV_1/1-1439 | APYSVTVYHNNYP-NSDVG-TRICLCKGSINLT-A--------------TTYNNCWGYGC | 156 |
| Mink_CoV_WD1127/1-1438 | SPYSATVYHKNGE-MARYDSGRFCLCKGAINTTVA--------------TGSGNCWGYDC | 160 |
| Rhinolophus_affinis_bat_CoV_HKU-2_related/1-1119 | ------------------------------------------------------------ | 0 |
| Rhinolophus_bat_CoV_HKU2/1-1128 | ------------------------------------------------------------ | 0 |
| HCoV_OC43/1-1361 | KVIK----------------DR-VMYSEFPAITIGS----TFVNTSYSVVVQPRTINSTQ | 151 |
| HCoV_HKU1/1-1356 | KLYV----------------NK-TLYSEFSTIVIGS----VFINNSYTIVVQPH------ | 142 |
| MERS_CoV/1-1353 | QFAN----------------GF-VVRIGAAANSTGT----VIISPSTSATIRKIYPAFML | 149 |
| Bat_SL_CoV_ZC45/1-1246 | NIIR----------------GW-IFGTTLDNTSQSL----LIVNNATNVIIKVCNF---- | 133 |
| Bat_SL_CoV_ZXC21/1-1245 | NIVR----------------GW-IFGTTLDNTSQSL----LIVNNATNVIIKVCNF---- | 132 |
| Pangolin_CoV_MP789/1-1265 | NIVR----------------GW-IFGTTLDNTSQSL----LIVNNATNVIIKVCNF---- | 133 |
| Bat_CoV_RaTG13/1-1269 | NIIR----------------GW-IFGTTLDSKTQSL----LIVNNATNVVIKVCEF---- | 133 |
| SARS-CoV2/1-1273 | NIIR----------------GW-IFGTTLDSKTQSL----LIVNNATNVVIKVCEF---- | 133 |
| Bat_SL_CoV_Rs4084/1-1256 | NVIR----------------GW-VFGSTMNNKSQSV----IIMNNSTNLVIRACNF---- | 131 |
| Bat_SL_CoV_RsSHC014/1-1256 | NVIR----------------GW-VFGSTMNNKSQSV----IIMNNSTNLVIRACNF---- | 131 |
| Bat_SL_CoV_Rs7327/1-1256 | NVIR----------------GW-VFGSTMNNKSQSV----IIMNNSTNLVIRACNF---- | 131 |
| Bat_SL_CoV_Rs9401/1-1256 | NVIR----------------GW-VFGSTMNNKSQSV----IIMNNSTNLVIRACNF---- | 131 |
| Bat_SL_CoV_WIV1/1-1256 | NVIR----------------GW-VFGSTMNNKSQSV----IIMNNSTNLVIRACNF---- | 131 |
| Bat_SL_CoV_Rs3367/1-1256 | NVIR----------------GW-VFGSTMNNKSQSV----IIMNNSTNLVIRACNF---- | 131 |
| Bat_SL_CoV_Rs4231/1-1255 | NVVR----------------GW-VFGSTMNNKSQSV----IIINNSTNVVIRACNF---- | 130 |
| Bat_SL_CoV_Rs4874/1-1255 | NVVR----------------GW-VFGSTMNNKSQSV----IIINNSTNVVIRACNF---- | 130 |
| SL_CoV_WIV16/1-1255 | NVVR----------------GW-VFGSTMNNKSQSV----IIINNSTNVVIRACNF---- | 130 |
| SARS_CoV_civet010/1-1255 | NVVR----------------GW-VFGSTMNNKSQSV----IIINNSTNVVIRACNF---- | 130 |
| SARS_CoV_Tor2/1-1255 | NVVR----------------GW-VFGSTMNNKSQSV----IIINNSTNVVIRACNF---- | 130 |
| Bat_SL_CoV__Rf4092/1-1234 | NVVR----------------GW-IFGTTLDNTTQSA----LLVNNGSAITIQVCYF---- | 129 |
| BtRf_CoV_JL2012/1-1236 | NVVR----------------GW-IFGTTLDNTTQSA----LLVNNGSAITIEVCYF---- | 131 |
| BtRs_YN2013/1-1233 | NIVR----------------GW-IFGSTLDSTSQSA----IILNNSTNLIVRVCNF---- | 127 |
| Bat_CoV_Rp/Shaanxi2011/1-1240 | NVIR----------------GW-IFGSTFDNRSQSA----IIVNNSTHILVKVCNF---- | 134 |
| Bat_SARS_CoV_Rf1/2004/1-1241 | NVIR----------------GW-VFGSTFDNTTQSA----IIVNNSTHIIIRVCYF---- | 136 |
| Bat_CoV/Yunnan2011/1-1241 | NVIR----------------GW-IFGSTMDNSTQSA----IIVNNSTHIIIRVCNF---- | 136 |

| | | |
|---|---|---|
| SARS_CoV_Rs_672/2006/1-1241 | NVVR----------------GW-IFGSTMDNTTQSA----IIVNNSTHIIIRVCYF---- | 136 |
| Bat_SL_CoV_Rs4081/1-1241 | NVVR----------------GW-IFGSTMDNTTQSA----IIVNNSTHIIIRVCYF---- | 136 |
| Bat_SL_CoV_Rs4255/1-1241 | NVVR----------------GW-IFGSTMDNTTQSA----IIVNNSTHIIIRVCYF---- | 136 |
| Bat_SARS_CoV_Rm1/2004/1-1241 | NVIR----------------GW-IFGSSFDNTTQSA----IIVNNSTHIIIRVCNF---- | 136 |
| Bat_SARS_CoV_HKU3-1/1-1242 | NVIR----------------GW-IFGSSFDNTTQSA----VIVNNSTHIIIRVCNF---- | 136 |
| Bat_SARS_CoV_Rp3/1-1241 | NVIR----------------GW-IFGSTFDNTTQSA----VIVNNSTHIIIRVCNF---- | 136 |
| Bat_SL_CoV_As6526/1-1241 | NVIR----------------GW-IFGSTFDNTTQSA----VIVNNSTHIIIRVCNF---- | 136 |
| Bat_SL_CoV_Rs4237/1-1241 | NVIR----------------GW-IFGSTFDNTTQSA----VIVNNSTHIIIRVCNF---- | 136 |
| Bat_SL_CoV_Rs4247/1-1242 | NVIR----------------GW-IFGSTFDNTTQSA----VIVNNSTHIIIRVCNF---- | 136 |

| | | |
|---|---|---|
| HCoV_NL63/1-1356 | T---------FDFLSNASSSF----DCIVNLLFTEQLGAPLGITISGETVRLHLYNV--T | 157 |
| Felis_Catus_CoV_NDL/UU88/1-1467 | GNISEHHEWSASLGDGGQCVFNRRFSLDTTLTANDFYGFQWTDTYVDVYLSGTVTKVWIE | 221 |
| Ferret_CoV/1-1438 | T---------VRS-------FRLC--ADESICHERILGLKWSSSEVVAYLAGEVYSYKLS | 196 |
| Canine_CoV/1-1457 | R---------LQHTGTQHTKFPICPSNIGSNCGNMLYGLQWFTDEVVAYLHGAVYRINFE | 207 |
| Mink_CoV_WD1133/1-1429 | K---------LNHY------FPIC--NNTDHCGHFLYGLKWSQTELFAYLYGYVYNIKLV | 193 |
| Mink_CoV_1/1-1439 | R---------LNTT------FATC--NVTGMCGDLLYGLQWSNSELMAYSSGAIYRIKID | 199 |
| Mink_CoV_WD1127/1-1438 | K---------INST------FWNC--NN-TGCGDLLYGLQWSNSELMAYLSGDIYRIKLY | 202 |
| Rhinolophus_affinis_bat_CoV_HKU-2_related/1-1119 | -----------------------------------------------------MKFI--- | 4 |
| Rhinolophus_bat_CoV_HKU2/1-1128 | -----------------------------------------------------MKLF--- | 4 |
| HCoV_OC43/1-1361 | ------------------------------------------------------------ | 151 |
| HCoV_HKU1/1-1356 | ------------------------------------------------------------ | 142 |
| MERS_CoV/1-1353 | GS------------------------------------------SVGNFSDGKMGRF--- | 164 |
| Bat_SL_CoV_ZC45/1-1246 | ------------------------------------------------------------ | 133 |
| Bat_SL_CoV_ZXC21/1-1245 | ------------------------------------------------------------ | 132 |
| Pangolin_CoV_MP789/1-1265 | ------------------------------------------------------------ | 133 |
| Bat_CoV_RaTG13/1-1269 | ------------------------------------------------------------ | 133 |
| SARS-CoV2/1-1273 | ------------------------------------------------------------ | 133 |
| Bat_SL_CoV_Rs4084/1-1256 | ------------------------------------------------------------ | 131 |
| Bat_SL_CoV_RsSHC014/1-1256 | ------------------------------------------------------------ | 131 |
| Bat_SL_CoV_Rs7327/1-1256 | ------------------------------------------------------------ | 131 |
| Bat_SL_CoV_Rs9401/1-1256 | ------------------------------------------------------------ | 131 |
| Bat_SL_CoV_WIV1/1-1256 | ------------------------------------------------------------ | 131 |
| Bat_SL_CoV_Rs3367/1-1256 | ------------------------------------------------------------ | 131 |
| Bat_SL_CoV_Rs4231/1-1255 | ------------------------------------------------------------ | 130 |
| Bat_SL_CoV_Rs4874/1-1255 | ------------------------------------------------------------ | 130 |
| SL_CoV_WIV16/1-1255 | ------------------------------------------------------------ | 130 |
| SARS_CoV_civet010/1-1255 | ------------------------------------------------------------ | 130 |
| SARS_CoV_Tor2/1-1255 | ------------------------------------------------------------ | 130 |
| Bat_SL_CoV__Rf4092/1-1234 | ------------------------------------------------------------ | 129 |

```
BtRf_CoV_JL2012/1-1236                                  ------------------------------------------------------------ 131
BtRs_YN2013/1-1233                                      ------------------------------------------------------------ 127
Bat_CoV_Rp/Shaanxi2011/1-1240                           ------------------------------------------------------------ 134
Bat_SARS_CoV_Rf1/2004/1-1241                            ------------------------------------------------------------ 136
Bat_CoV/Yunnan2011/1-1241                               ------------------------------------------------------------ 136
SARS_CoV_Rs_672/2006/1-1241                             ------------------------------------------------------------ 136
Bat_SL_CoV_Rs4081/1-1241                                ------------------------------------------------------------ 136
Bat_SL_CoV_Rs4255/1-1241                                ------------------------------------------------------------ 136
Bat_SARS_CoV_Rm1/2004/1-1241                            ------------------------------------------------------------ 136
Bat_SARS_CoV_HKU3-1/1-1242                              ------------------------------------------------------------ 136
Bat_SARS_CoV_Rp3/1-1241                                 ------------------------------------------------------------ 136
Bat_SL_CoV_As6526/1-1241                                ------------------------------------------------------------ 136
Bat_SL_CoV_Rs4237/1-1241                                ------------------------------------------------------------ 136
Bat_SL_CoV_Rs4247/1-1242                                ------------------------------------------------------------ 136


HCoV_NL63/1-1356                                        RTFYVPAAYK--------LTKLSVKCYFNYSCVFSVVNATVTVN---VTTHNGRVVNYTV 206
Felis_Catus_CoV_NDL/UU88/1-1467                         NDWDVVEA------SISYKWNKINYGYYIQFVNRTTYYAYNNTGGSNYTRLRLSECNS-- 273
Ferret_CoV/1-1438                                       NHWYNNVTIK-------TQTNANQIYWWFNPVRDLSYYNVNKTV---DSTIVVSNCT--- 243
Canine_CoV/1-1457                                       NQWSGTVTLGDMRATTAQTAGAVVDLWWFNPVYDITYYRVNSK----NSTTVIFNCT--- 260
Mink_CoV_WD1133/1-1429                                  NHWFNNASLALM-----RAASVGQEGWWFNPVRNLTYYSVYKN----SSSTIVQNCT--- 241
Mink_CoV_1/1-1439                                       NLWFNNATISNTIASNITGESLLMNSWWFNPVYNLTYYRVNKT----ETTVVVQNCT--- 252
Mink_CoV_WD1127/1-1438                                  NTWFNNATISNT----FASNGTTSNSWWFNPVYNLTYYRVNKT----DNTVVVQNCT--- 251
Rhinolophus_affinis_bat_CoV_HKU-2_related/1-1119        ---I-AACLL----HLVCG-CEYVAIDWFKPVFY--------TPKGLVNGTTVISGVYPS 47
Rhinolophus_bat_CoV_HKU2/1-1128                         ---I-VFVLL----FRVCYCCDYVDFRLFNGIFS--------TSRGLSNTTTVITGAYPS 48
HCoV_OC43/1-1361                                        -----------------DGYNKL------------------------QGLLEVSVCQYNM 170
HCoV_HKU1/1-1356                                        -----------------------------------------------NGVLEITACQYTM 155
MERS_CoV/1-1353                                         ---F-NHTLV----LLPDGCGTLL--RAFYCILE--------PRSGNHCPAGNSYTSFAT 206
Bat_SL_CoV_ZC45/1-1246                                  ----------------------------------------------------------DF 135
Bat_SL_CoV_ZXC21/1-1245                                 ----------------------------------------------------------DF 134
Pangolin_CoV_MP789/1-1265                               ----------------------------------------------------------QF 135
Bat_CoV_RaTG13/1-1269                                   ----------------------------------------------------------QF 135
SARS-CoV2/1-1273                                        ----------------------------------------------------------QF 135
Bat_SL_CoV_Rs4084/1-1256                                ----------------------------------------------------------EL 133
Bat_SL_CoV_RsSHC014/1-1256                              ----------------------------------------------------------EL 133
Bat_SL_CoV_Rs7327/1-1256                                ----------------------------------------------------------EL 133
Bat_SL_CoV_Rs9401/1-1256                                ----------------------------------------------------------EL 133
Bat_SL_CoV_WIV1/1-1256                                  ----------------------------------------------------------EL 133
Bat_SL_CoV_Rs3367/1-1256                                ----------------------------------------------------------EL 133
Bat_SL_CoV_Rs4231/1-1255                                ----------------------------------------------------------EL 132
```

| | | |
|---|---|---|
| Bat_SL_CoV_Rs4874/1-1255 | ----------------------------------------------------------EL | 132 |
| SL_CoV_WIV16/1-1255 | ----------------------------------------------------------EL | 132 |
| SARS_CoV_civet010/1-1255 | ----------------------------------------------------------EL | 132 |
| SARS_CoV_Tor2/1-1255 | ----------------------------------------------------------EL | 132 |
| Bat_SL_CoV__Rf4092/1-1234 | ----------------------------------------------------------QF | 131 |
| BtRf_CoV_JL2012/1-1236 | ----------------------------------------------------------QF | 133 |
| BtRs_YN2013/1-1233 | ----------------------------------------------------------EL | 129 |
| Bat_CoV_Rp/Shaanxi2011/1-1240 | ----------------------------------------------------------VL | 136 |
| Bat_SARS_CoV_Rf1/2004/1-1241 | ----------------------------------------------------------NL | 138 |
| Bat_CoV/Yunnan2011/1-1241 | ----------------------------------------------------------NL | 138 |
| SARS_CoV_Rs_672/2006/1-1241 | ----------------------------------------------------------NL | 138 |
| Bat_SL_CoV_Rs4081/1-1241 | ----------------------------------------------------------NL | 138 |
| Bat_SL_CoV_Rs4255/1-1241 | ----------------------------------------------------------NL | 138 |
| Bat_SARS_CoV_Rm1/2004/1-1241 | ----------------------------------------------------------NL | 138 |
| Bat_SARS_CoV_HKU3-1/1-1242 | ----------------------------------------------------------NL | 138 |
| Bat_SARS_CoV_Rp3/1-1241 | ----------------------------------------------------------NL | 138 |
| Bat_SL_CoV_As6526/1-1241 | ----------------------------------------------------------NL | 138 |
| Bat_SL_CoV_Rs4237/1-1241 | ----------------------------------------------------------NL | 138 |
| Bat_SL_CoV_Rs4247/1-1242 | ----------------------------------------------------------NL | 138 |

| | | |
|---|---|---|
| HCoV_NL63/1-1356 | CDDCNGYTDN-------------IF------SVQQDGRIPNGFPFNNWFLLTNGSTLVDG | 247 |
| Felis_Catus_CoV_NDL/UU88/1-1467 | -TYCAGYAKN-------------VF------VP-IDGKIPESFSFSNWFLLSDKSTLVQG | 312 |
| Ferret_CoV/1-1438 | -SDCSGYAAN-------------IF------AVETGGFIPSSFSFNNWFVLTNSSTIVSG | 283 |
| Canine_CoV/1-1457 | -DQCASYVDN-------------IF------TTQPGGFIPSDFSFNNWFLLTNSSTVLNG | 300 |
| Mink_CoV_WD1133/1-1429 | -GNCEEYANN-------------IF------STEPGGIIPEGFSFNNWFVLTNDSTVLDG | 281 |
| Mink_CoV_1/1-1439 | -NNCADYVNN-------------IF------STEDGGIIPEGFSFNNWFVLTNDSTITDG | 292 |
| Mink_CoV_WD1127/1-1438 | -NNCADYVNN-------------IF------STESGGIIPEGFSFNNWFVLTNDSTITDG | 291 |
| Rhinolophus_affinis_bat_CoV_HKU-2_related/1-1119 | VNKSDWFCPTDVVRPYGHGRGIGLFIQTASA---ASDDTYGGYTFSVTP----------- | 93 |
| Rhinolophus_bat_CoV_HKU2/1-1128 | TNKAKWFCPTNVGRPVGTGVGIGVYAQTAQASYETGGSGAGGYTFSVSP----------- | 97 |
| HCoV_OC43/1-1361 | CEYPQTICH--------------------------------------------------- | 179 |
| HCoV_HKU1/1-1356 | CEYPHTICK--------------------------------------------------- | 164 |
| MERS_CoV/1-1353 | YHTPATDCS--------------------------------------------------- | 215 |
| Bat_SL_CoV_ZC45/1-1246 | CYDPYLSGY--------------------------------------------------- | 144 |
| Bat_SL_CoV_ZXC21/1-1245 | CYDPYLSGY--------------------------------------------------- | 143 |
| Pangolin_CoV_MP789/1-1265 | CYDPYLSGY--------------------------------------------------- | 144 |
| Bat_CoV_RaTG13/1-1269 | CNDPFLGVY--------------------------------------------------- | 144 |
| SARS-CoV2/1-1273 | CNDPFLGVY--------------------------------------------------- | 144 |
| Bat_SL_CoV_Rs4084/1-1256 | CDNPFFVVL--------------------------------------------------- | 142 |
| Bat_SL_CoV_RsSHC014/1-1256 | CDNPFFVVL--------------------------------------------------- | 142 |

| | | |
|---|---|---|
| Bat_SL_CoV_Rs7327/1-1256 | CDNPFFVVL--------------------------------------------------- | 142 |
| Bat_SL_CoV_Rs9401/1-1256 | CDNPFFVVL--------------------------------------------------- | 142 |
| Bat_SL_CoV_WIV1/1-1256 | CDNPFFVVL--------------------------------------------------- | 142 |
| Bat_SL_CoV_Rs3367/1-1256 | CDNPFFVVL--------------------------------------------------- | 142 |
| Bat_SL_CoV_Rs4231/1-1255 | CDNPFFAVS--------------------------------------------------- | 141 |
| Bat_SL_CoV_Rs4874/1-1255 | CDNPFFAVS--------------------------------------------------- | 141 |
| SL_CoV_WIV16/1-1255 | CDNPFFAVS--------------------------------------------------- | 141 |
| SARS_CoV_civet010/1-1255 | CDNPFFVVS--------------------------------------------------- | 141 |
| SARS_CoV_Tor2/1-1255 | CDNPFFAVS--------------------------------------------------- | 141 |
| Bat_SL_CoV__Rf4092/1-1234 | CDNPAFVVT--------------------------------------------------- | 140 |
| BtRf_CoV_JL2012/1-1236 | CDNPAFIIR--------------------------------------------------- | 142 |
| BtRs_YN2013/1-1233 | CKVPLFVVF--------------------------------------------------- | 138 |
| Bat_CoV_Rp/Shaanxi2011/1-1240 | CTEPMFTVS--------------------------------------------------- | 145 |
| Bat_SARS_CoV_Rf1/2004/1-1241 | CKDPMYTVS--------------------------------------------------- | 147 |
| Bat_CoV/Yunnan2011/1-1241 | CKEPMFTVS--------------------------------------------------- | 147 |
| SARS_CoV_Rs_672/2006/1-1241 | CKEPMYAIS--------------------------------------------------- | 147 |
| Bat_SL_CoV_Rs4081/1-1241 | CKEPMYAIS--------------------------------------------------- | 147 |
| Bat_SL_CoV_Rs4255/1-1241 | CKEPMYAIS--------------------------------------------------- | 147 |
| Bat_SARS_CoV_Rm1/2004/1-1241 | CKEPMYTVS--------------------------------------------------- | 147 |
| Bat_SARS_CoV_HKU3-1/1-1242 | CKEPMYTVS--------------------------------------------------- | 147 |
| Bat_SARS_CoV_Rp3/1-1241 | CKEPMYTVS--------------------------------------------------- | 147 |
| Bat_SL_CoV_As6526/1-1241 | CKEPMYTVS--------------------------------------------------- | 147 |
| Bat_SL_CoV_Rs4237/1-1241 | CKEPMYTVS--------------------------------------------------- | 147 |
| Bat_SL_CoV_Rs4247/1-1242 | CKEPMYTVS--------------------------------------------------- | 147 |

| | | |
|---|---|---|
| HCoV_NL63/1-1356 | VSRLYQPLRLTCLWPVPGLKSSTGFVYFNATGSDVNCNGYQHNSVVDVMRYNLNFSANSL | 307 |
| Felis_Catus_CoV_NDL/UU88/1-1467 | RVLSRQPVLVQCLRPVPTLSNNSAVASFNND-------AFCPNVSADVLRFNLNFSDTDV | 365 |
| Ferret_CoV/1-1438 | KFVSSQPLLVNCLTPVPSFGDETSIINFDT--VPSQCNGATVNGSFDVVRFNLNFTANVA | 341 |
| Canine_CoV/1-1457 | KLVTRQPLLVNCLWPVPSFKGAASTFCFEGA-GFDQCNGAVLNNTVDVIRFNLNFTADVQ | 359 |
| Mink_CoV_WD1133/1-1429 | RFLTRQPLLVNCLWPVPSFSEISQEFCFASA-DFTQCNGYKLNSTADVIRFNLNFTHETA | 340 |
| Mink_CoV_1/1-1439 | RFLTSQPLLVNCLWPVPSFSETSQNFCFDTE-DFAQCNGYKLNSTADVIRFNLNFTHETV | 351 |
| Mink_CoV_WD1127/1-1438 | RFLTRQPLLVNCLWPVPSFSETSQNFCFNTE-DFAQCNGYRLNSTADVIRFNLNFTHETV | 350 |
| Rhinolophus_affinis_bat_CoV_HKU-2_related/1-1119 | ---EYVTNLTWALWVHRAWGINS-------------------PVTVRLCRWSNTL----- | 126 |
| Rhinolophus_bat_CoV_HKU2/1-1128 | ---KHVTNLTWSLWVHRPWGANA-------------------NVTVRLCRWWQKF----- | 130 |
| HCoV_OC43/1-1361 | ------PNLGNHRKE--LWHLDT---------------------GVVSCLYKRNF----- | 205 |
| HCoV_HKU1/1-1356 | ------SK-GSSRNE--SWHFDK---------------------SEPLCLFKKNF----- | 189 |
| MERS_CoV/1-1353 | ------DGNYNRNASLNSFKEYF---------------------NLRNCTFMYTYNIT-- | 246 |
| Bat_SL_CoV_ZC45/1-1246 | ------Y-HNNKTWSIREFAVYS---------------------SYANCTFEYVSKSF-- | 174 |
| Bat_SL_CoV_ZXC21/1-1245 | ------Y-HNNKTWSIREFAVYS---------------------FYANCTFEYVSKSF-- | 173 |

```
Pangolin_CoV_MP789/1-1265                           ------Y-HNNKTWSTREFAVYS---------------------SYANCTFEYVSKSF-- 174
Bat_CoV_RaTG13/1-1269                               ------YHKNNKSWMESEFRVYS---------------------SANNCTFEYVSQPF-- 175
SARS-CoV2/1-1273                                    ------YHKNNKSWMESEFRVYS---------------------SANNCTFEYVSQPF-- 175
Bat_SL_CoV_Rs4084/1-1256                            ------KSNNTQIPSY----IFN---------------------NAFNCTFEYVSKDF-- 169
Bat_SL_CoV_RsSHC014/1-1256                          ------KSNNTQIPSY----IFN---------------------NAFNCTFEYVSKDF-- 169
Bat_SL_CoV_Rs7327/1-1256                            ------KSNNTQIPSY----IFN---------------------NAFNCTFEYVSKDF-- 169
Bat_SL_CoV_Rs9401/1-1256                            ------KSNNTQIPSY----IFN---------------------NAFNCTFEYVSKDF-- 169
Bat_SL_CoV_WIV1/1-1256                              ------KSNNTQIPSY----IFN---------------------NAFNCTFEYVSKDF-- 169
Bat_SL_CoV_Rs3367/1-1256                            ------KSNNTQIPSY----IFN---------------------NAFNCTFEYVSKDF-- 169
Bat_SL_CoV_Rs4231/1-1255                            ------KPTGTQTHTM----IFD---------------------NAFNCTFEYISDSF-- 168
Bat_SL_CoV_Rs4874/1-1255                            ------KPTGTQTHTM----IFD---------------------NAFNCTFEYISDSF-- 168
SL_CoV_WIV16/1-1255                                 ------KPTGTQTHTM----IFD---------------------NAFNCTFEYISDSF-- 168
SARS_CoV_civet010/1-1255                            ------KPMGTQTHTM----IFD---------------------NAFNCTFEYISDAF-- 168
SARS_CoV_Tor2/1-1255                                ------KPMGTQTHTM----IFD---------------------NAFNCTFEYISDAF-- 168
Bat_SL_CoV__Rf4092/1-1234                           ------AGAQTSTA-I----Y-T---------------------NLRNCTYVDTLRDL-- 165
BtRf_CoV_JL2012/1-1236                              ------DGAQINTA-I----Y-I---------------------NLRNCTYVDTLRDL-- 167
BtRs_YN2013/1-1233                                  ------KSNNSQLS-H----LFS---------------------DSFNCTFEYVSRAF-- 164
Bat_CoV_Rp/Shaanxi2011/1-1240                       ------RNQHYKSW-V----Y-Q---------------------HARNCTYDVAYPSF-- 170
Bat_SARS_CoV_Rf1/2004/1-1241                        ------AGTQKSSW-V----Y-Q---------------------SAFNCTYDRVEKSF-- 172
Bat_CoV/Yunnan2011/1-1241                           ------RGVHFSSW-V----Y-Q---------------------SAFNCTYDRVEKSF-- 172
SARS_CoV_Rs_672/2006/1-1241                         ------NEQHYKSW-V----Y-Q---------------------NAYNCTYDRVEQSF-- 172
Bat_SL_CoV_Rs4081/1-1241                            ------NEQHYKSW-V----Y-Q---------------------NAYNCTYDRVEQSF-- 172
Bat_SL_CoV_Rs4255/1-1241                            ------NEQHYKSW-V----Y-Q---------------------NAYNCTYDRVGQSF-- 172
Bat_SARS_CoV_Rm1/2004/1-1241                        ------KGTQQSSW-V----Y-Q---------------------SAFNCTYDRVEKSF-- 172
Bat_SARS_CoV_HKU3-1/1-1242                          ------RGTQQNAW-V----Y-Q---------------------SAFNCTYDRVEKSF-- 172
Bat_SARS_CoV_Rp3/1-1241                             ------RGAQQSSW-V----Y-Q---------------------SAFNCTYDRVEKSF-- 172
Bat_SL_CoV_As6526/1-1241                            ------RGTQQSSW-V----Y-Q---------------------SAFNCTYDRVERSF-- 172
Bat_SL_CoV_Rs4237/1-1241                            ------RGTQQSSW-V----Y-Q---------------------SAFNCTYDRVERSF-- 172
Bat_SL_CoV_Rs4247/1-1242                            ------RGTQQSSW-V----Y-Q---------------------SAFNCTYDRVERSF-- 172
                                                                                                      :

HCoV_NL63/1-1356                                    DNLKSGV--IVFKTLQYDVL-FYCSNSSS-------GVLDTTIPFGPSSQPYYCFINSTI 357
Felis_Catus_CoV_NDL/UU88/1-1467                     YTASNNDEQLYFTFEDNTTASIACYSSANVTDFQPANNSASHIPFGKTMHSYFCFANFSH 425
Ferret_CoV/1-1438                                   SSSGTS--FLALNTTGGVVLYLSCFNETKT---TNLYMSEGALPFGTHEGALYCYVSYNE 396
Canine_CoV/1-1457                                   SGMGAT--VFSLNTTGGVILEISCYNDTVS---ESSFYSYGEIPFGVTDGPRYCYVLYNG 414
Mink_CoV_WD1133/1-1429                              AASGTT--FFELNTTGGVVLYISCYNQSRT---EAQ-VSEGFVPFGNHEGSLYCYVSYNE 394
Mink_CoV_1/1-1439                                   AASGTT--FFMLNTTGGVVLYVSCYNQSRT---EAQ-VSDSFIPFGNHDGSLYCYVSYNE 405
Mink_CoV_WD1127/1-1438                              AASGTT--FFELNTTGGVVLHISCYNQSRT---EAV-TSEGFLPFGYHENSLYCYVSYNE 404
Rhinolophus_affinis_bat_CoV_HKU-2_related/1-1119    -------------------------PFNITSHTDNAN---PTFAID--CLINGSYPSHS- 155
Rhinolophus_bat_CoV_HKU2/1-1128                     -------------------------SFNETAHFQPAG---PSSAFE--CLVNGSFPSSQ- 159
```

```
HCoV_OC43/1-1361                      -------------------------TYDV-------NADYLYFHFYQEGGTFYAYFTDTG 233
HCoV_HKU1/1-1356                      -------------------------TYNV-------STDWLYFHFYQERGTFYAYYADSG 217
MERS_CoV/1-1353                       -------------------------EDEILEWFGITQTAQGVHLF--SSRYVDLYGG--- 276
Bat_SL_CoV_ZC45/1-1246                -------------------------MLNISGN-GGLFNTLREFVFRNVDGHFKIYSKFTP 208
Bat_SL_CoV_ZXC21/1-1245               -------------------------MLNISGN-GGLFNTLREFVFRNVDGHFKIYSKFTP 207
Pangolin_CoV_MP789/1-1265             -------------------------MLDIAGK-SGLFDTLREFVFRNVDGYFKIYSKYTP 208
Bat_CoV_RaTG13/1-1269                 -------------------------LMDLEGK-QGNFKNLREFVFKNIDGYFKIYSKHTP 209
SARS-CoV2/1-1273                      -------------------------LMDLEGK-QGNFKNLREFVFKNIDGYFKIYSKHTP 209
Bat_SL_CoV_Rs4084/1-1256              -------------------------NLDLGEK-PGNFKDLREFVFRNKDGFLHVYSGYQP 203
Bat_SL_CoV_RsSHC014/1-1256            -------------------------NLDLGEK-PGNFKDLREFVFRNKDGFLHVYSGYQP 203
Bat_SL_CoV_Rs7327/1-1256              -------------------------NLDLGEK-PGNFKDLREFVFRNKDGFLHVYSGYQP 203
Bat_SL_CoV_Rs9401/1-1256              -------------------------NLDLGEK-PGNFKDLREFVFRNKDGFLHVYSGYQP 203
Bat_SL_CoV_WIV1/1-1256                -------------------------NLDLGEK-PGNFKDLREFVFRNKDGFLHVYSGYQP 203
Bat_SL_CoV_Rs3367/1-1256              -------------------------NLDLGEK-PGNFKDLREFVFRNKDGFLHVYSGYQP 203
Bat_SL_CoV_Rs4231/1-1255              -------------------------SLDVAEK-SGNFKHLREFVFKNKDGFLYVYKGYQP 202
Bat_SL_CoV_Rs4874/1-1255              -------------------------SLDVAEK-SGNFKHLREFVFKNKDGFLYVYKGYQP 202
SL_CoV_WIV16/1-1255                   -------------------------SLDVAEK-SGNFKHLREFVFKNKDGFLYVYKGYQP 202
SARS_CoV_civet010/1-1255              -------------------------SLDVSEK-SGNFKHLREFVFKNKDGFLYVYKGYQP 202
SARS_CoV_Tor2/1-1255                  -------------------------SLDVSEK-SGNFKHLREFVFKNKDGFLYVYKGYQP 202
Bat_SL_CoV__Rf4092/1-1234             -------------------------PLSLTEV-DGGFKHLREFVFKTSDGFLHIYGAYQP 199
BtRf_CoV_JL2012/1-1236                -------------------------PLSFAEV-DGGFKHLREFVFKNSDGFLHIYGAYQP 201
BtRs_YN2013/1-1233                    -------------------------SLDIREQS-GNFVDLREFVFRNRNGFLHIYEGYEA 198
Bat_CoV_Rp/Shaanxi2011/1-1240         -------------------------QLDVSLKNNVNFQHLREFIFKNVDGFLKIYSSYEP 205
Bat_SARS_CoV_Rf1/2004/1-1241          -------------------------QLDTSPKT-GNFTDLREFVFKNRDGFFTAYQTYTP 206
Bat_CoV/Yunnan2011/1-1241             -------------------------QLDTAPKT-GNFKDLREYVFKNRDGFLSVYHSYTP 206
SARS_CoV_Rs_672/2006/1-1241           -------------------------QLDTAPQT-GNFKDLREYVFKNKDGFLSVYNAYSP 206
Bat_SL_CoV_Rs4081/1-1241              -------------------------QLDTAPQT-GNFKDLREYVFKNKDGFLSVYNAYSP 206
Bat_SL_CoV_Rs4255/1-1241              -------------------------QLDTAPQT-GNFKDLREYVFKNKDGFLSVYNAYSP 206
Bat_SARS_CoV_Rm1/2004/1-1241          -------------------------QLDTAPKT-GNFKDLREYVFKNKGGFLRVYQTYTA 206
Bat_SARS_CoV_HKU3-1/1-1242            -------------------------QLDTTPKT-GNFKDLREYVFKNRDGFLSVYQTYTA 206
Bat_SARS_CoV_Rp3/1-1241               -------------------------QLDTAPKT-GNFKDLREYVFKNRDGFLSVYQTYTA 206
Bat_SL_CoV_As6526/1-1241              -------------------------QLDTAPKT-GNFKDLREYVFKNRDGFLSVYQTYTA 206
Bat_SL_CoV_Rs4237/1-1241              -------------------------QLDTAPKT-GNFKDLREYVFKNRDGFLSVYQTYTA 206
Bat_SL_CoV_Rs4247/1-1242              -------------------------QLDTAPKT-GNFKDLREYVFKNRDGFLSVYQTYTA 206
                                                                                  :         :


HCoV_NL63/1-1356                      NTTHVSTFVGILPPTVREIVVARTGQFYINGFKYFDLGFIEAVNF------------NVT 405
Felis_Catus_CoV_NDL/UU88/1-1467       -SVVSRQFLGILPPTVRELAFGRDGSIFVNGYKYFSLPPIKSVNF------------SIS 472
Ferret_CoV/1-1438                     -T--SFKYIGVLPPSVKEIAVSKWGFVYINGYNYFQTFPIDSVAF------------NLT 441
Canine_CoV/1-1457                     -T--ALKYLGTLPPSVKEIAISKWGHFYINGYNFFSTFPIDCISF------------NLT 459
```

```
Mink_CoV_WD1133/1-1429                                -T--LQKFLGVLPPSVKEIAISKDGGFYINGYNYFQTFPIDCISF------------NLT 439
Mink_CoV_1/1-1439                                     -T--LQKFIGVLPPSVKEIAISKDGGFYINGYNYFQVFPIDCISF------------NIT 450
Mink_CoV_WD1127/1-1438                                -T--LQKFLGVLPPSVKEIAISKDGGFYINGYNYFQTFPIDCISF------------NLT 449
Rhinolophus_affinis_bat_CoV_HKU-2_related/1-1119      --------GKGYTLGVSWYN---------D-LVRIVFPPTSFEIQLDGLQWINVQFTSPV 197
Rhinolophus_bat_CoV_HKU2/1-1128                       --------HKGYMFGVTWYN---------D-FVRIIFPPTVFELQLDGLQWEYVQFTGPV 201
HCoV_OC43/1-1361                                      V----------------VTKFLFNVYLGMA-LSHYYVMPLTCNSK-------------VK 263
HCoV_HKU1/1-1356                                      M----------------PTTFLFSLYLGTL-LSHYYVLPLTCNAI-----------SSNT 249
MERS_CoV/1-1353                                       -----------------NMFQFATLPVYDT-IKYYSIIPHSIRSI------------QSD 306
Bat_SL_CoV_ZC45/1-1246                                VNLN-----RGLPTGLSVLQPLVELPVSIN-ITKFRTLLTIHRGDPMP-----N---NGW 254
Bat_SL_CoV_ZXC21/1-1245                               VNLN-----RGLPTGLSVLQPLVELPVSIN-ITKFRTLLTIHRGDPMS-----N---NGW 253
Pangolin_CoV_MP789/1-1265                             VNVN-----SNLPIGFSALEPLVEIPAGIN-ITKFRTLLTIHRGDPMP-----N---NGW 254
Bat_CoV_RaTG13/1-1269                                 INLV-----RDLPPGFSALEPLVDLPIGIN-ITRFQTLLALHRSYLTP-----GDSSSGW 258
SARS-CoV2/1-1273                                      INLV-----RDLPQGFSALEPLVDLPIGIN-ITRFQTLLALHRSYLTP-----GDSSSGW 258
Bat_SL_CoV_Rs4084/1-1256                              ISAA-----SGLPTGFNALKPIFKLPLGIN-ITNFRTLLTAFPPR-----------PDYW 246
Bat_SL_CoV_RsSHC014/1-1256                            ISAA-----SGLPTGFNALKPIFKLPLGIN-ITNFRTLLTAFPPR-----------PDYW 246
Bat_SL_CoV_Rs7327/1-1256                              ISAA-----SGLPTGFNALKPIFKLPLGIN-ITNFRTLLTAFPPR-----------PDYW 246
Bat_SL_CoV_Rs9401/1-1256                              ISAA-----SGLPTGFNALKPIFKLPLGIN-ITNFRTLLTAFPPR-----------PDYW 246
Bat_SL_CoV_WIV1/1-1256                                ISAA-----SGLPTGFNALKPIFKLPLGIN-ITNFRTLLTAFPPR-----------PDYW 246
Bat_SL_CoV_Rs3367/1-1256                              ISAA-----SGLPTGFNALKPIFKLPLGIN-ITNFRTLLTAFPPR-----------PDYW 246
Bat_SL_CoV_Rs4231/1-1255                              IDVV-----RDLPSGFNILKPIFKLPLGIN-ITNFRAILTAFLPA-----------QDTW 245
Bat_SL_CoV_Rs4874/1-1255                              IDVV-----RDLPSGFNILKPIFKLPLGIN-ITNFRAILTAFLPA-----------QDTW 245
SL_CoV_WIV16/1-1255                                   IDVV-----RDLPSGFNILKPIFKLPLGIN-ITNFRAILTAFLPA-----------QDTW 245
SARS_CoV_civet010/1-1255                              IDVV-----RDLPSGFNTLKPIFKLPLGIK-ITNFRAILTAFSPA-----------QGTW 245
SARS_CoV_Tor2/1-1255                                  IDVV-----RDLPSGFNTLKPIFKLPLGIN-ITNFRAILTAFSPA-----------QDIW 245
Bat_SL_CoV__Rf4092/1-1234                             YDHAIG-ATAALPAQFLPLKPLWKLPLGLN-ITSYKVVTTLKPTN--------------- 242
BtRf_CoV_JL2012/1-1236                                YDLAIG-ATAALPAQFLPLKPLWKLPLGLN-ITNYKVVTTLKPTN--------------- 244
BtRs_YN2013/1-1233                                    ISIV-----RGLPAGFNVLKPLLKIPFGLN-VTSFKTFLAVYRVA-----------AGSI 241
Bat_CoV_Rp/Shaanxi2011/1-1240                         INVV-----SGIPSGFSVLKPVMSLPLGIN-ITGMRVVMTMFSNT-----------QANF 248
Bat_SARS_CoV_Rf1/2004/1-1241                          VNLL-----RGLPSGLSVLKPILKLPFGIN-ITSFRVVMAMFSKT-----------TSNY 249
Bat_CoV/Yunnan2011/1-1241                             VDII-----RGIPVGFSVLKPILKLPIGIN-ITSFKVVMTMYSQT-----------TSNF 249
SARS_CoV_Rs_672/2006/1-1241                           IDIP-----RGLPVGFSVLKPILKLPISIN-ITSFKVVMSMFSRT-----------TSNF 249
Bat_SL_CoV_Rs4081/1-1241                              IDIP-----RGLPVGFSVLKPILKLPIGIN-ITSFKVVMSMFSRT-----------TSNF 249
Bat_SL_CoV_Rs4255/1-1241                              IDIP-----RGLPVGFSVLKPILKLPIGIN-ITSFKVVMSMFSRT-----------TSNF 249
Bat_SARS_CoV_Rm1/2004/1-1241                          VNLP-----RGFPAGFSVLRPILKLPFGIN-ITSYRVVMTMFSQF-----------NSNF 249
Bat_SARS_CoV_HKU3-1/1-1242                            VNLP-----RGLPTGFSVLKPILKLPFGIN-ITSYRVVMAMFSQT-----------TSNF 249
Bat_SARS_CoV_Rp3/1-1241                               VNLP-----RGLPIGFSVLRPILKLPFGIN-ITSYRVVMAMFSQT-----------TSNF 249
Bat_SL_CoV_As6526/1-1241                              VNLP-----RGLPIGFSVLRPILKLPFGIN-ITSYRVVMAMFSQT-----------TSNF 249
Bat_SL_CoV_Rs4237/1-1241                              VNLP-----RGLPTGFSVLRPILKLPFGIN-ITSYRVVMAMFSQT-----------TSNF 249
Bat_SL_CoV_Rs4247/1-1242                              VNLP-----RGLPTGFSVLRPILKLPFGIN-ITSYRVVMAMFSQT-----------TSNF 249
```

| | | |
|---|---|---|
| HCoV_NL63/1-1356 | TASATDFWTVAFATFVDVLVNVSATNIQNLLYCDS-PFEKLQCEHLQFGLQDGFYSANFL | 464 |
| Felis_Catus_CoV_NDL/UU88/1-1467 | SVENYGFWTIAYTNYTDVMVDVNGTGITRLLYCDS-PLNRIKCQQMKHELPDGFYSASML | 531 |
| Ferret_CoV/1-1438 | TGNSGAFWTIAYTTFTDVLLEVSDTQIKSVTYCNS-HINDIKCSQMSENLPDGFYPVSQH | 500 |
| Canine_CoV/1-1457 | TGASGAFWTIAYTSYTEALVQVENTAIKKVTYCNS-HINNIKCSQLTANLQNGFYPVASS | 518 |
| Mink_CoV_WD1133/1-1429 | TGNTGAFWTIAYTSYTDVMVDVENTAIKRVIYCNS-HINDIRCNQLTPSLPDGFYPVSPK | 498 |
| Mink_CoV_1/1-1439 | TGNTGAFWTIAYTSYTDVMVDVENTAIKRVIYCNS-HINDIRCNQLTPSLPDGFYPVSPK | 509 |
| Mink_CoV_WD1127/1-1438 | TGNTGAFWTIAYTSYTDVMVDVENTAIKRVVYCNS-HIDDIRCNQLTPNLPDGFYPVSPK | 508 |
| Rhinolophus_affinis_bat_CoV_HKU-2_related/1-1119 | SYGRSTKFNVVQSTSSVLVSTDSSGNVNNFVYCDSGFVESLQCKLGRFDVASGVYSNSEV | 257 |
| Rhinolophus_bat_CoV_HKU2/1-1128 | NAGRMTKFNVVTEISSVLVLTDQSGAVTRYSYCADGFVNGLQCKLRLFDIPPGVYSNSEV | 261 |
| HCoV_OC43/1-1361 | NGFTLEYWVTPLTSRQYLLAFNQDGIIFNAVDCMSDFMSEIKCKTQSIAPPTGVYELNGY | 323 |
| HCoV_HKU1/1-1356 | DNETLQYWVTPLSKRQYLLKFDNRGVITNAVDCSSSFFSEIQCKTKSLLPNTGVYDLSGF | 309 |
| MERS_CoV/1-1353 | RKAWAAFYVYKLQPLTFLLDFSVDGYIRRAIDCGFNDLSQLHCSYESFDVESGVYSVSSF | 366 |
| Bat_SL_CoV_ZC45/1-1246 | TAFSAAYFVGYLKPRTFMLKYNENGTITDAVDCALDPLSETKCTLKSLTVQKGIYQTSNF | 314 |
| Bat_SL_CoV_ZXC21/1-1245 | TAFSAAYFVGYLKPRTFMLKYNENGTITDAVDCALDPLSETKCTLKSLSVQKGIYQTSNF | 313 |
| Pangolin_CoV_MP789/1-1265 | TVFSAAYYVGYLAPRTFMLNYNENGTITDAVDCALDPLSEAKCTLKSLTVEKGIYQTSNF | 314 |
| Bat_CoV_RaTG13/1-1269 | TAGAAAYYVGYLQPRTFLLKYNENGTITDAVDCALDPLSETKCTLKSFTVEKGIYQTSNF | 318 |
| SARS-CoV2/1-1273 | TAGAAAYYVGYLQPRTFLLKYNENGTITDAVDCALDPLSETKCTLKSFTVEKGIYQTSNF | 318 |
| Bat_SL_CoV_Rs4084/1-1256 | GTSAAAYFVGYLKPTTFMLKYDENGTITDAVDCSQNPLAELKCSVKSFEIDKGIYQTSNF | 306 |
| Bat_SL_CoV_RsSHC014/1-1256 | GTSAAAYFVGYLKPTTFMLKYDENGTITDAVDCSQNPLAELKCSVKSFEIDKGIYQTSNF | 306 |
| Bat_SL_CoV_Rs7327/1-1256 | GTSAAAYFVGYLKPTTFMLKYDENGTITDAVDCSQNPLAELKCSVKSFEIDKGIYQTSNF | 306 |
| Bat_SL_CoV_Rs9401/1-1256 | GTSAAAYFVGYLKPTTFMLKYDENGTITDAVDCSQNPLAELKCSVKSFEIDKGIYQTSNF | 306 |
| Bat_SL_CoV_WIV1/1-1256 | GTSAAAYFVGYLKPTTFMLKYDENGTITDAVDCSQNPLAELKCSVKSFEIDKGIYQTSNF | 306 |
| Bat_SL_CoV_Rs3367/1-1256 | GTSAAAYFVGYLKPTTFMLKYDENGTITDAVDCSQNPLAELKCSVKSFEIDKGIYQTSNF | 306 |
| Bat_SL_CoV_Rs4231/1-1255 | GTSAAAYFVGYLKPATFMLKYDENGTITDAVDCSQNPLAELKCSVKSFEIDKGIYQTSNF | 305 |
| Bat_SL_CoV_Rs4874/1-1255 | GTSAAAYFVGYLKPATFMLKYDENGTITDAVDCSQNPLAELKCSVKSFEIDKGIYQTSNF | 305 |
| SL_CoV_WIV16/1-1255 | GTSAAAYFVGYLKPATFMLKYDENGTITDAVDCSQNPLAELKCSVKSFEIDKGIYQTSNF | 305 |
| SARS_CoV_civet010/1-1255 | GTSAAAYFVGYLKPTTFMLKYDENGTITDAVDCSQNPLAELKCSVKSFEIDKGIYQTSNF | 305 |
| SARS_CoV_Tor2/1-1255 | GTSAAAYFVGYLKPTTFMLKYDENGTITDAVDCSQNPLAELKCSVKSFEIDKGIYQTSNF | 305 |
| Bat_SL_CoV__Rf4092/1-1234 | QAFQAVYIVGNLKHTTMMLSFNENGTMSNAVDCSQDPLAELKCTLKLSDVGKGIYQTSNF | 302 |
| BtRf_CoV_JL2012/1-1236 | QAFQAAYIVGNLKHTTMMLSFNENGTMSNAIDCSQDPLAELKCTLKQFDVGKGIYQTSNF | 304 |
| BtRs_YN2013/1-1233 | SVASSAYYVGYLKPLTFMLSYDLNGTIKNAVDCSQDPLAELKCTIKNFNVSKGIYQTSNF | 301 |
| Bat_CoV_Rp/Shaanxi2011/1-1240 | LTENAAYYVGYLKPRTFMLQFNTNGTIVNAVDCSQDPLSELKCTLKNFNITKGIYQTSNF | 308 |
| Bat_SARS_CoV_Rf1/2004/1-1241 | VPESAAYYVGNLKQSTFMLSFNQNGTIVDAVDCSQDPLAELKCTTKSFNVSKGIYQTSNF | 309 |
| Bat_CoV/Yunnan2011/1-1241 | LSESAAYYVGNLKYVTFMFQFNENGTIADAVDCSQNPLAELKCTLKNFNVSKGIYQTSNF | 309 |
| SARS_CoV_Rs_672/2006/1-1241 | LPEVAAYFVGNLKYSTFMLNFNENGTITDAIDCAQNPLSELKCTIKNFNVSKGIYQTSNF | 309 |
| Bat_SL_CoV_Rs4081/1-1241 | LPEIAAYFVGNLKYSTFMLNFNENGTITDAIDCAQNPLSELKCTIKNFNVSKGIYQTSNF | 309 |
| Bat_SL_CoV_Rs4255/1-1241 | LPEVAAYFVGNLKYSTFMLNFNENGTITDAIDCAQNPLSELKCTIKNFNVSKGIYQTSNF | 309 |
| Bat_SARS_CoV_Rm1/2004/1-1241 | LPESAAYYVGNLKYTTFMLSFNENGTITDAVDCSQNPLAELKCTIKNFNVSKGIYQTSNF | 309 |
| Bat_SARS_CoV_HKU3-1/1-1242 | LPESAAYYVGNLKYSTFMLRFNENGTITDAVDCSQNPLAELKCTIKNFNVDKGIYQTSNF | 309 |

```
Bat_SARS_CoV_Rp3/1-1241                            LPESAAYYVGNLKYTTFMLSFNENGTITNAIDCAQNPLAELKCTIKNFNVSKGIYQTSNF 309
Bat_SL_CoV_As6526/1-1241                           LPESAAYYVGNLKYTTFMLRFNENGTITDAIDCAQNPLAELKCTIKNFNVSKGIYQTSNF 309
Bat_SL_CoV_Rs4237/1-1241                           LPESAAYYVGNLKYTTFMLSFNENGTITDAIDCAQNPLAELKCTIKNFNVSKGIYQTSNF 309
Bat_SL_CoV_Rs4247/1-1242                           LPESAAYYVGNLKYTTFMLSFNENGTITDAIDCAQNPLAELKCTIKNFNVSKGIYQTSNF 309
                                                                     .       :     *    .   :*         *.*


HCoV_NL63/1-1356                                   DDNVLPETY-----------------VALPIYYQHTDINFTATASFGGSCY----V---- 499
Felis_Catus_CoV_NDL/UU88/1-1467                    VKKDLPKTF-----------------VTMPQFYNWMNVTLHVVLNDTEKGT----DIILA 570
Ferret_CoV/1-1438                                  SLPNVNKTF-----------------VTLPANFEHTSINVTGNVLLI-----------YY 532
Canine_CoV/1-1457                                  EVGLVNKSV-----------------VLLPSFFAHTAVNITIDLGMKR---------SGY 552
Mink_CoV_WD1133/1-1429                             AIGFINKTF-----------------VTLPANFDHVYVNITGNVNLKAERG------RPY 535
Mink_CoV_1/1-1439                                  AIGLVNKTF-----------------VTLPANFDHVYVNITGNVKLKIQRG------QLY 546
Mink_CoV_WD1127/1-1438                             AIGFVNKTF-----------------VTLPANFDHVYVNITGNVRLKIQRG------RPY 545
Rhinolophus_affinis_bat_CoV_HKU-2_related/1-1119   EYP--TALYTVVNNMSACPSRPESYCG--SNFCPPKRAVFSNCVVNYTNWVGNPTDVLDY 313
Rhinolophus_bat_CoV_HKU2/1-1128                    EYP--VALYTVVHNMSVCPQRPESYCG--SNYCPFKRVVFSNCVVNYTSWTSG--LLRDY 315
HCoV_OC43/1-1361                                   TVQPIADVYRRKLNLPNCNIEAWLNDKSVPSPLNWERKTFSNCNFNMSSLMSFIQ----- 378
HCoV_HKU1/1-1356                                   TVKPVATVHRRIPDLPDCDIDKWLNNFNVPSPLNWERKIFSNCNFNLSTLLRLVH----- 364
MERS_CoV/1-1353                                    EAKPSGSVVEQAE-GVECDFSPLLS-GTPPQVYNFKRLVFTNCNYNLTKLLSLFS----- 419
Bat_SL_CoV_ZC45/1-1246                             RVQPTQSVVRFPNITNVCPFHKVFNATRFPSVYAWERTKISDCIADYTVFYNSTS----- 369
Bat_SL_CoV_ZXC21/1-1245                            RVQPTQSIVRFPNITNVCPFHKVFNATRFPSVYAWERTKISDCIADYTVFYNSTS----- 368
Pangolin_CoV_MP789/1-1265                          RVQPTESIVRFPNITNLCPFGEVFNATTFASVYAWNRKRISNCVADYSVLYNSTS----- 369
Bat_CoV_RaTG13/1-1269                              RVQPTDSIVRFPNITNLCPFGEVFNATTFASVYAWNRKRISNCVADYSVLYNSTS----- 373
SARS-CoV2/1-1273                                   RVQPTESIVRFPNITNLCPFGEVFNATRFASVYAWNRKRISNCVADYSVLYNSAS----- 373
Bat_SL_CoV_Rs4084/1-1256                           RVAPSKEVVRFPNITNLCPFGEVFNATTFPSVYAWERKRISNCVADYSILYNSTS----- 361
Bat_SL_CoV_RsSHC014/1-1256                         RVAPSKEVVRFPNITNLCPFGEVFNATTFPSVYAWERKRISNCVADYSVLYNSTS----- 361
Bat_SL_CoV_Rs7327/1-1256                           RVAPSKEVVRFPNITNLCPFGEVFNATTFPSVYAWERKRISNCVADYSVLYNSTS----- 361
Bat_SL_CoV_Rs9401/1-1256                           RVAPSKEVVRFPNITNLCPFGEVFNATTFPSVYAWERKRISNCVADYSVLYNSTS----- 361
Bat_SL_CoV_WIV1/1-1256                             RVAPSKEVVRFPNITNLCPFGEVFNATTFPSVYAWERKRISNCVADYSVLYNSTS----- 361
Bat_SL_CoV_Rs3367/1-1256                           RVAPSKEVVRFPNITNLCPFGEVFNATTFPSVYAWERKRISNCVADYSVLYNSTS----- 361
Bat_SL_CoV_Rs4231/1-1255                           RVAPSKEVVRFPNITNLCPFGEVFNATTFPSVYAWERKRISNCVADYSVLYNSTS----- 360
Bat_SL_CoV_Rs4874/1-1255                           RVAPSKEVVRFPNITNLCPFGEVFNATTFPSVYAWERKRISNCVADYSVLYNSTS----- 360
SL_CoV_WIV16/1-1255                                RVAPSKEVVRFPNITNLCPFGEVFNATTFPSVYAWERKRISNCVADYSVLYNSTS----- 360
SARS_CoV_civet010/1-1255                           RVVPSGDVVRFPNITNLCPFGEVFNATKFPSVYAWERKRISNCVADYSVLYNSTS----- 360
SARS_CoV_Tor2/1-1255                               RVVPSGDVVRFPNITNLCPFGEVFNATKFPSVYAWERKKISNCVADYSVLYNSTF----- 360
Bat_SL_CoV__Rf4092/1-1234                          RVQPTVDVVRFPNITNLCPFDAVFNATRFPSVYAWERVKISNCVADYTAFYNSTS----- 357
BtRf_CoV_JL2012/1-1236                             RVQPTVDVARFPNITNVCPFDKVFNATRFPSVYAWERTKISDCVADYTVFYNSTS----- 359
BtRs_YN2013/1-1233                                 RVSPTREVVRFPNITNRCPFDSIFNASRFPSVYAWERTKISDCVADYTVLYNSTL----- 356
Bat_CoV_Rp/Shaanxi2011/1-1240                      RVSPTQEVVRFPNITNRCPFDKVFNATRFPSVYAWERTKISDCVADYTVLYNSTS----- 363
Bat_SARS_CoV_Rf1/2004/1-1241                       RVSPVTEVVRFPNITNLCPFDKVFNATRFPSVYAWERTKISDCVADYTVFYNSTS----- 364
Bat_CoV/Yunnan2011/1-1241                          RVSPSTEVIRFPNITNRCPFDRVFNASRFPSVYAWERTKISDCVADYTVLYNSTS----- 364
```

```
SARS_CoV_Rs_672/2006/1-1241                           RVSPTHEVIRFPNITNRCPFDKVFNASRFPNVYAWERTKISDCVADYTVLYNSTS----- 364
Bat_SL_CoV_Rs4081/1-1241                              RVSPTHEVVRFPNITNRCPFDKVFNASRFPNVYAWERTKISDCVADYTVLYNSTS----- 364
Bat_SL_CoV_Rs4255/1-1241                              RVSPTHEVIRFPNITNRCPFDKVFNASRFPNVYAWERTKISDCVADYTVLYNSTS----- 364
Bat_SARS_CoV_Rm1/2004/1-1241                          RVTPTQEVVRFPNITNRCPFDKVFNASRFPNVYAWERTKISDCVADYTVLYNSTS----- 364
Bat_SARS_CoV_HKU3-1/1-1242                            RVSPTQEVIRFPNITNRCPFDKVFNATRFPNVYAWERTKISDCVADYTVLYNSTS----- 364
Bat_SARS_CoV_Rp3/1-1241                               RVSPTQEVIRFPNITNRCPFDKVFNATRFPNVYAWERTKISDCVADYTVLYNSTS----- 364
Bat_SL_CoV_As6526/1-1241                              RVSPTQEVVRFPNITNRCPFDKVFNATRFPSVYAWERTKISDCVADYTVLYNSTS----- 364
Bat_SL_CoV_Rs4237/1-1241                              RVSPTQEVIRFPNITNRCPFDKVFNASRFPNVYAWERTKISDCVADYTVLYNSTS----- 364
Bat_SL_CoV_Rs4247/1-1242                              RVSPTQEVIRFPNITNRCPFDKVFNASRFPNVYAWERTKISDCVADYTVLYNSTS----- 364
                                                                                             .

HCoV_NL63/1-1356                                      -----------------CKPHQVNISLNGNTSVC------------------------- 516
Felis_Catus_CoV_NDL/UU88/1-1467                       KAN-----ELASLADIHFEIAQANGSVTNVTSLC------------------------- 599
Ferret_CoV/1-1438                                     AHP-----VFRSGN---V-----TLHPQGTQTIC------------------------- 553
Canine_CoV/1-1457                                     GQP-----IASTLSNITL-----PMQDNNTDVYC------------------------- 576
Mink_CoV_WD1133/1-1429                                NNG-----GNATLS----------------FQGC------------------------- 548
Mink_CoV_1/1-1439                                     NGG-----GNATLS----------------FQGC------------------------- 559
Mink_CoV_WD1127/1-1438                                NNG-----GNATLS----------------FQGC------------------------- 558
Rhinolophus_affinis_bat_CoV_HKU-2_related/1-1119      THLMLPNGLFNPFI--VC----NGL--NKLVEGCVPGFVLR-----VGRGTATN---GTV 357
Rhinolophus_bat_CoV_HKU2/1-1128                       QHLVLPNGKFNPFT--EC----NGL--NRIVDDCVTGFVLR-----VGRGTAVN---RTV 359
HCoV_OC43/1-1361                                      ---------ADSF---TC----NNIDAAKIYGMCFSSITIDKFAIPNGRKVDLQLGNLGY 422
HCoV_HKU1/1-1356                                      ---------TDSF---SC----NNFDESKIYGSCFKSIVLDKFAIPNSRRSDLQLGSSGF 408
MERS_CoV/1-1353                                       ---------VNDF---TC----SQISPAAIASNCYSSLILDYFSYPLSMKSDLSVSSAGP 463
Bat_SL_CoV_ZC45/1-1246                                ---------FSTF---KC----YGVSPSKLIDLCFTSVYADTFLIRFSEVRQVAPGQTGV 413
Bat_SL_CoV_ZXC21/1-1245                               ---------FSTF---KC----YGVSPSKLIDLCFTSVYADTFLIRFSEVRQVAPGQTGV 412
Pangolin_CoV_MP789/1-1265                             ---------FSTF---KC----YGVSPTKLNDLCFTNVYADSFVVRGDEVRQIAPGQTGR 413
Bat_CoV_RaTG13/1-1269                                 ---------FSTF---KC----YGVSPTKLNDLCFTNVYADSFVITGDEVRQIAPGQTGK 417
SARS-CoV2/1-1273                                      ---------FSTF---KC----YGVSPTKLNDLCFTNVYADSFVIRGDEVRQIAPGQTGK 417
Bat_SL_CoV_Rs4084/1-1256                              ---------FSTF---KC----YGVSATKLNDLCFSNVYADSFVVKGDDVRQIAPGQTGV 405
Bat_SL_CoV_RsSHC014/1-1256                            ---------FSTF---KC----YGVSATKLNDLCFSNVYADSFVVKGDDVRQIAPGQTGV 405
Bat_SL_CoV_Rs7327/1-1256                              ---------FSTF---KC----YGVSATKLNDLCFSNVYADSFVVKGDDVRQIAPGQTGV 405
Bat_SL_CoV_Rs9401/1-1256                              ---------FSTF---KC----YGVSATKLNDLCFSNVYADSFVVKGDDVRQIAPGQTGV 405
Bat_SL_CoV_WIV1/1-1256                                ---------FSTF---KC----YGVSATKLNDLCFSNVYADSFVVKGDDVRQIAPGQTGV 405
Bat_SL_CoV_Rs3367/1-1256                              ---------FSTF---KC----YGVSATKLNDLCFSNVYADSFVVKGDDVRQIAPGQTGV 405
Bat_SL_CoV_Rs4231/1-1255                              ---------FSTF---KC----YGVSATKLNDLCFSNVYADSFVVKGDDVRQIAPGQTGV 404
Bat_SL_CoV_Rs4874/1-1255                              ---------FSTF---KC----YGVSATKLNDLCFSNVYADSFVVKGDDVRQIAPGQTGV 404
SL_CoV_WIV16/1-1255                                   ---------FSTF---KC----YGVSATKLNDLCFSNVYADSFVVKGDDVRQIAPGQTGV 404
SARS_CoV_civet010/1-1255                              ---------FSTF---KC----YGVSATKLNDLCFSNVYADSFVVKGDDVRQIAPGQTGV 404
SARS_CoV_Tor2/1-1255                                  ---------FSTF---KC----YGVSATKLNDLCFSNVYADSFVVKGDDVRQIAPGQTGV 404
Bat_SL_CoV__Rf4092/1-1234                             ---------FSTF---KC----YGVSPSKLIDLCFTSVYADTFLIRFSEVRQVAPGETGV 401
```

```
BtRf_CoV_JL2012/1-1236                                ---------FSTF---NC----YGVSPSKLIDLCFTSVYADTFLIRFSEVRQVAPGQTGV 403
BtRs_YN2013/1-1233                                    ---------FSTF---KC----YGVSPSKLIDLCFTSVYADTFLIRFSEVRQVAPGETGV 400
Bat_CoV_Rp/Shaanxi2011/1-1240                         ---------FSTF---KC----YGVSPSKLIDLCFTSVYADTFLIRSSEVRQVAPGETGV 407
Bat_SARS_CoV_Rf1/2004/1-1241                          ---------FSTF---NC----YGVSPSKLIDLCFTSVYADTFLIRFSEVRQVAPGQTGV 408
Bat_CoV/Yunnan2011/1-1241                             ---------FSTF---KC----YGVSPSKLIDLCFTSVYADTFLIRFSEVRQIAPGETGV 408
SARS_CoV_Rs_672/2006/1-1241                           ---------FSTF---KC----YGVSPSKLIDLCFTSVYADTFLIRSSEVRQVAPGETGV 408
Bat_SL_CoV_Rs4081/1-1241                              ---------FSTF---KC----YGVSPSKLIDLCFTSVYADTFLIRSSEVRQVAPGETGV 408
Bat_SL_CoV_Rs4255/1-1241                              ---------FSTF---KC----YGVSPSKLIDLCFTSVYADTFLIRSSEVRQVAPGETGV 408
Bat_SARS_CoV_Rm1/2004/1-1241                          ---------FSTF---KC----YGVSPSKLIDLCFTSVYADTFLIRSSEVRQVAPGETGV 408
Bat_SARS_CoV_HKU3-1/1-1242                            ---------FSTF---KC----YGVSPSKLIDLCFTSVYADTFLIRSSEVRQVAPGETGV 408
Bat_SARS_CoV_Rp3/1-1241                               ---------FSTF---KC----YGVSPSKLIDLCFTSVYADTFLIRSSEVRQVAPGETGV 408
Bat_SL_CoV_As6526/1-1241                              ---------FSTF---KC----YGVSPSKLIDLCFTSVYADTFLIRSSEVRQVAPGETGV 408
Bat_SL_CoV_Rs4237/1-1241                              ---------FSTF---KC----YGVSPSKLIDLCFTSVYADTFLIRSSEVRQVAPGETGV 408
Bat_SL_CoV_Rs4247/1-1242                              ---------FSTF---KC----YGVSPSKLIDLCFTSVYADTFLIRSSEVRQVAPGETGV 408
                                                                                       *
```

```
HCoV_NL63/1-1356                                      VRTSHFSIR-------YIYNRVKSGS--------PGDSSWHIYLKSGTCPFSFSKLNNFQ 561
Felis_Catus_CoV_NDL/UU88/1-1467                       VQTRQLALFYKYTSLQGL-----------------YTYSNLVELQNHDCPFSPQQFNNYL 642
Ferret_CoV/1-1438                                     VNTTQFTFNFDSQCHQL-----AGGG-------CDGIGASMISIDSGNCPFSPDKLNQHL 601
Canine_CoV/1-1457                                     IRSNQFSVYTHSTCKSSLWDNNFNRD-------CTDVLYATAVIKTGTCPFSFDKLNNYL 629
Mink_CoV_WD1133/1-1429                                IAVSQFTVNLNHTCTIT---NAGSSG-------CTNAYEANVYMTSGSCPFAFDRLNNHM 598
Mink_CoV_1/1-1439                                     ITASQFTVNLNHTCTLD----SASGA-------CEKAYAANVYMTSGSCPFAFDRLNNHM 608
Mink_CoV_WD1127/1-1438                                IDVSQFTVNVNHTCTLI----DANGQ-------CTGAYEARVYVTSGSCPFAFDRLNNHM 607
Rhinolophus_affinis_bat_CoV_HKU-2_related/1-1119      P--TPYLN---------------------------------------------------- 363
Rhinolophus_bat_CoV_HKU2/1-1128                       I--TPYLK---------------------------------------------------- 365
HCoV_OC43/1-1361                                      LQSFNYRIDTTATSCQLYYNLPAANVSVSRFNPSTWNKRFGFIEDSVFKPRPAGVLTNHD 482
HCoV_HKU1/1-1356                                      LQSSNYKIDTTSSSCQLYYSLPAINVTINNYNPSSWNRRYGFN------NF---NLSSHS 459
MERS_CoV/1-1353                                       ISQFNYKQSFSNPTCLILATVPHNLT-----------TITKPLKYSYINKCSRLLSDDRT 512
Bat_SL_CoV_ZC45/1-1246                                IADYNYKLPDDFTGCVIAWNTAKQDV---------GNYFYRSHRSTKLKPFERDLSSDEN 464
Bat_SL_CoV_ZXC21/1-1245                               IADYNYKLPDDFTGCVIAWNTAKQDT---------GHYFYRSHRSTKLKPFERDLSSDEN 463
Pangolin_CoV_MP789/1-1265                             IADYNYKLPDDFTGCVIAWNSNNLDSKVG----GNYNYLYRLFRKSNLKPFERDISTEIY 469
Bat_CoV_RaTG13/1-1269                                 IADYNYKLPDDFTGCVIAWNSKHIDAKEG----GNFNYLYRLFRKANLKPFERDISTEIY 473
SARS-CoV2/1-1273                                      IADYNYKLPDDFTGCVIAWNSNNLDSKVG----GNYNYLYRLFRKSNLKPFERDISTEIY 473
Bat_SL_CoV_Rs4084/1-1256                              IADYNYKLPDDFLGCVLAWNTNSKDSSTS----GNYNYLYRWVRRSKLNPYERDLSNDIY 461
Bat_SL_CoV_RsSHC014/1-1256                            IADYNYKLPDDFLGCVLAWNTNSKDSSTS----GNYNYLYRWVRRSKLNPYERDLSNDIY 461
Bat_SL_CoV_Rs7327/1-1256                              IADYNYKLPDDFMGCVLAWNTRNIDATST----GNYNYKYRSLRHGKLRPFERDISNVPF 461
Bat_SL_CoV_Rs9401/1-1256                              IADYNYKLPDDFMGCVLAWNTRNIDATST----GNYNYKYRSLRHGKLRPFERDISNVPF 461
Bat_SL_CoV_WIV1/1-1256                                IADYNYKLPDDFTGCVLAWNTRNIDATQT----GNYNYKYRSLRHGKLRPFERDISNVPF 461
Bat_SL_CoV_Rs3367/1-1256                              IADYNYKLPDDFTGCVLAWNTRNIDATQT----GNYNYKYRSLRHGKLRPFERDISNVPF 461
Bat_SL_CoV_Rs4231/1-1255                              IADYNYKLPDDFLGCVLAWNTNSKDSSTS----GNYNYLYRWVRRSKLNPYERDLSNDIY 460
```

| | | |
|---|---|---|
| Bat_SL_CoV_Rs4874/1-1255 | IADYNYKLPDDFTGCVLAWNTRNIDATQT----GNYNYKYRSLRHGKLRPFERDISNVPF | 460 |
| SL_CoV_WIV16/1-1255 | IADYNYKLPDDFTGCVLAWNTRNIDATQT----GNYNYKYRSLRHGKLRPFERDISNVPF | 460 |
| SARS_CoV_civet010/1-1255 | IADYNYKLPDDFMGCVLAWNTRNIDATST----GNYNYKYRYLRHGKLRPFERDISNVPF | 460 |
| SARS_CoV_Tor2/1-1255 | IADYNYKLPDDFMGCVLAWNTRNIDATST----GNYNYKYRYLRHGKLRPFERDISNVPF | 460 |
| Bat_SL_CoV__Rf4092/1-1234 | IADYNYKLPDDFTGCVIAWNTAKQDVG---------SYFYRSHRSSKLKPFERDLSSD-- | 450 |
| BtRf_CoV_JL2012/1-1236 | IADYNYKLPDDFIGCVIAWNTAKQDVG---------SYFYRSHRSSKLKPFERDLSSE-- | 452 |
| BtRs_YN2013/1-1233 | IADYNYRLPDDFTGCVIAWNTANQDVG---------SYFYRSHRSTKLKPFERDLSSD-- | 449 |
| Bat_CoV_Rp/Shaanxi2011/1-1240 | IADYNYKLPDDFTGCVIAWNTANQDQG---------QYYYRSSRKEKLKPFERDLSSD-- | 456 |
| Bat_SARS_CoV_Rf1/2004/1-1241 | IADYNYKLPDDFTGCVIAWNTAKQDVG---------SYFYRSHRSSKLKPFERDLSSE-- | 457 |
| Bat_CoV/Yunnan2011/1-1241 | IADYNYKLPDEFTGCVIAWNTANQDRG---------QYYYRSSRKTKLKPFERDLSSD-- | 457 |
| SARS_CoV_Rs_672/2006/1-1241 | IADYNYKLPDDFTGCVIAWNTAKQDQG---------QYYYRSSRKTKLKPFERDLTSD-- | 457 |
| Bat_SL_CoV_Rs4081/1-1241 | IADYNYKLPDDFTGCVIAWNTAKQDQG---------QYYYRSSRKTKLKPFERDLTSD-- | 457 |
| Bat_SL_CoV_Rs4255/1-1241 | IADYNYKLPDDFTGCVIAWNTAKQDQG---------QYYYRSSRKTKLKPFERDLSSD-- | 457 |
| Bat_SARS_CoV_Rm1/2004/1-1241 | IADYNYKLPDDFTGCVIAWNTAQQDQG---------QYYYRSYRKEKLKPFERDLSSD-- | 457 |
| Bat_SARS_CoV_HKU3-1/1-1242 | IADYNYKLPDDFTGCVIAWNTAKHDTG---------NYYYRSHRKTKLKPFERDLSSDD- | 458 |
| Bat_SARS_CoV_Rp3/1-1241 | IADYNYKLPDDFTGCVIAWNTAKQDQG---------QYYYRSHRKTKLKPFERDLSSDE- | 458 |
| Bat_SL_CoV_As6526/1-1241 | IADYNYKLPDDFTGCVIAWNTAQQDKG---------QYYYRSSRKTKLKPFERDLSSDE- | 458 |
| Bat_SL_CoV_Rs4237/1-1241 | IADYNYKLPDDFTGCVIAWNTAKQDQG---------QYYYRSSRKTKLKPFERDLSSDE- | 458 |
| Bat_SL_CoV_Rs4247/1-1242 | IADYNYKLPDDFTGCVIAWNTAKQDTG---------HYYYRSHRKTKLKPFERDLSSDD- | 458 |

| | | |
|---|---|---|
| HCoV_NL63/1-1356 | KFK-TICFST--------------------VEVPGSCNFPLEATW--------------- | 585 |
| Felis_Catus_CoV_NDL/UU88/1-1467 | QFE-TLCFDV--------------------NPAVAGCRWSLVHDN--------------- | 666 |
| Ferret_CoV/1-1438 | AFE-TLCFST--------------------LPTGSDCAFSLTVQN--------------- | 625 |
| Canine_CoV/1-1457 | TFN-KLCLSL--------------------NPTGANCKFDVAART--------------- | 653 |
| Mink_CoV_WD1133/1-1429 | SFS-KLCFST--------------------VPLGDDCRLDLTVQT--------------- | 622 |
| Mink_CoV_1/1-1439 | SFA-KLCFSP--------------------VPLGDDCRLDLTVQT--------------- | 632 |
| Mink_CoV_WD1127/1-1438 | SFS-KICFSP--------------------VPLGDDCRLDLTVQT--------------- | 631 |
| Rhinolophus_affinis_bat_CoV_HKU-2_related/1-1119 | ------------------------------------------------------------ | 363 |
| Rhinolophus_bat_CoV_HKU2/1-1128 | ------------------------------------------------------------ | 365 |
| HCoV_OC43/1-1361 | VVYAQHCFKAPKNFCPCKLNGSCVGSGPGKNNGIGTCPAGTNYLT------------CDN | 530 |
| HCoV_HKU1/1-1356 | VVYSRYCFSVNNTFCPCAKPSFASSCK-SHKPPSASCPIGTNYRSCESTTVLDHTDWCRC | 518 |
| MERS_CoV/1-1353 | EVPQLVN---------------ANQYSPCVSI-VPSTVWEDGDYYRKQLSPLEGGGWLVA | 556 |
| Bat_SL_CoV_ZC45/1-1246 | ------------------------------------GVRTLSTY-----------DFNPN | 477 |
| Bat_SL_CoV_ZXC21/1-1245 | ------------------------------------GVRTLSTY-----------DFNPN | 476 |
| Pangolin_CoV_MP789/1-1265 | QAG----------------------STPCNGVEGFNCYFPLQSY-----------GFHPT | 496 |
| Bat_CoV_RaTG13/1-1269 | QAG----------------------SKPCNGQTGLNCYYPLYRY-----------GFYPT | 500 |
| SARS-CoV2/1-1273 | QAG----------------------STPCNGVEGFNCYFPLQSY-----------GFQPT | 500 |
| Bat_SL_CoV_Rs4084/1-1256 | SPG----------------------GQSCSAV-GPNCYNPLRPY-----------GFFTT | 487 |
| Bat_SL_CoV_RsSHC014/1-1256 | SPG----------------------GQSCSAV-GPNCYNPLRPY-----------GFFTT | 487 |

| | | |
|---|---|---|
| Bat_SL_CoV_Rs7327/1-1256 | SPD----------------------GKPCTPP-AFNCYWPLNDY-----------GFFTT | 487 |
| Bat_SL_CoV_Rs9401/1-1256 | SPD----------------------GKPCTPP-AFNCYWPLNDY-----------GFFTT | 487 |
| Bat_SL_CoV_WIV1/1-1256 | SPD----------------------GKPCTPP-AFNCYWPLNDY-----------GFYIT | 487 |
| Bat_SL_CoV_Rs3367/1-1256 | SPD----------------------GKPCTPP-AFNCYWPLNDY-----------GFYIT | 487 |
| Bat_SL_CoV_Rs4231/1-1255 | SPG----------------------GQSCSAI-GPNCYNPLRPY-----------GFFTT | 486 |
| Bat_SL_CoV_Rs4874/1-1255 | SPD----------------------GKPCTPP-AFNCYWPLNDY-----------GFYIT | 486 |
| SL_CoV_WIV16/1-1255 | SPD----------------------GKPCTPP-AFNCYWPLNDY-----------GFYIT | 486 |
| SARS_CoV_civet010/1-1255 | SSD----------------------GKPCTPP-APNCYWPLRGY-----------GFYTT | 486 |
| SARS_CoV_Tor2/1-1255 | SPD----------------------GKPCTPP-ALNCYWPLNDY-----------GFYTT | 486 |
| Bat_SL_CoV__Rf4092/1-1234 | ----------------------------------ENGVRTLSTY-----------DFNPN | 465 |
| BtRf_CoV_JL2012/1-1236 | ----------------------------------ENGVLTLSTY-----------DFNQN | 467 |
| BtRs_YN2013/1-1233 | ----------------------------------ENGVRTLSTY-----------DFNPN | 464 |
| Bat_CoV_Rp/Shaanxi2011/1-1240 | ----------------------------------ENGVYTLSTY-----------DFYPS | 471 |
| Bat_SARS_CoV_Rf1/2004/1-1241 | ----------------------------------ENGVRTLSTY-----------DFNQN | 472 |
| Bat_CoV/Yunnan2011/1-1241 | ----------------------------------ENGVRTLSTY-----------DFYPS | 472 |
| SARS_CoV_Rs_672/2006/1-1241 | ----------------------------------ENGVRTLSTY-----------DFYPN | 472 |
| Bat_SL_CoV_Rs4081/1-1241 | ----------------------------------ENGVRTLSTY-----------DFYPN | 472 |
| Bat_SL_CoV_Rs4255/1-1241 | ----------------------------------ENGVRTLSTY-----------DFYPT | 472 |
| Bat_SARS_CoV_Rm1/2004/1-1241 | ----------------------------------ENGVYTLSTY-----------DFYPS | 472 |
| Bat_SARS_CoV_HKU3-1/1-1242 | ----------------------------------GNGVYTLSTY-----------DFNPN | 473 |
| Bat_SARS_CoV_Rp3/1-1241 | -----------------------------------NGVRTLSTY-----------DFYPS | 472 |
| Bat_SL_CoV_As6526/1-1241 | -----------------------------------NGVRTLSTY-----------DFYPT | 472 |
| Bat_SL_CoV_Rs4237/1-1241 | -----------------------------------NGVRTLSTY-----------DFYPT | 472 |
| Bat_SL_CoV_Rs4247/1-1242 | ----------------------------------GNGVYTLSTY-----------DFNPN | 473 |

| | | |
|---|---|---|
| HCoV_NL63/1-1356 | ------------------------------------------------------------ | 585 |
| Felis_Catus_CoV_NDL/UU88/1-1467 | ------------------------------------------------------------ | 666 |
| Ferret_CoV/1-1438 | ------------------------------------------------------------ | 625 |
| Canine_CoV/1-1457 | ------------------------------------------------------------ | 653 |
| Mink_CoV_WD1133/1-1429 | ------------------------------------------------------------ | 622 |
| Mink_CoV_1/1-1439 | ------------------------------------------------------------ | 632 |
| Mink_CoV_WD1127/1-1438 | ------------------------------------------------------------ | 631 |
| Rhinolophus_affinis_bat_CoV_HKU-2_related/1-1119 | ------------------------------------------------------PSECFG | 369 |
| Rhinolophus_bat_CoV_HKU2/1-1128 | ------------------------------------------------------PNECFG | 371 |
| HCoV_OC43/1-1361 | LCTPDPITFKATGTYKCPQTKSLVGIGEHCSGLAVKSDYCGG------NSCTCRPQAFLG | 584 |
| HCoV_HKU1/1-1356 | SCLPDPITA--YDPRSCSQKKSLVGVGEHCAGFGVDEEKCGVLDGSYNVSCLCSTDAFLG | 576 |
| MERS_CoV/1-1353 | SGS----T---------------------------------------------------- | 560 |
| Bat_SL_CoV_ZC45/1-1246 | VPL----E---------------------------------------------------- | 481 |
| Bat_SL_CoV_ZXC21/1-1245 | VPL----E---------------------------------------------------- | 480 |

| | | |
|---|---|---|
| Pangolin_CoV_MP789/1-1265 | NGV----G---------------------------------------------------- | 500 |
| Bat_CoV_RaTG13/1-1269 | DGV----G---------------------------------------------------- | 504 |
| SARS-CoV2/1-1273 | NGV----G---------------------------------------------------- | 504 |
| Bat_SL_CoV_Rs4084/1-1256 | AGV----G---------------------------------------------------- | 491 |
| Bat_SL_CoV_RsSHC014/1-1256 | AGV----G---------------------------------------------------- | 491 |
| Bat_SL_CoV_Rs7327/1-1256 | NGI----G---------------------------------------------------- | 491 |
| Bat_SL_CoV_Rs9401/1-1256 | NGI----G---------------------------------------------------- | 491 |
| Bat_SL_CoV_WIV1/1-1256 | NGI----G---------------------------------------------------- | 491 |
| Bat_SL_CoV_Rs3367/1-1256 | NGI----G---------------------------------------------------- | 491 |
| Bat_SL_CoV_Rs4231/1-1255 | AGV----G---------------------------------------------------- | 490 |
| Bat_SL_CoV_Rs4874/1-1255 | NGI----G---------------------------------------------------- | 490 |
| SL_CoV_WIV16/1-1255 | NGI----G---------------------------------------------------- | 490 |
| SARS_CoV_civet010/1-1255 | SGI----G---------------------------------------------------- | 490 |
| SARS_CoV_Tor2/1-1255 | TGI----G---------------------------------------------------- | 490 |
| Bat_SL_CoV__Rf4092/1-1234 | VPL----D---------------------------------------------------- | 469 |
| BtRf_CoV_JL2012/1-1236 | VPL----E---------------------------------------------------- | 471 |
| BtRs_YN2013/1-1233 | VPL----D---------------------------------------------------- | 468 |
| Bat_CoV_Rp/Shaanxi2011/1-1240 | VPL----D---------------------------------------------------- | 475 |
| Bat_SARS_CoV_Rf1/2004/1-1241 | VPL----E---------------------------------------------------- | 476 |
| Bat_CoV/Yunnan2011/1-1241 | VPL----E---------------------------------------------------- | 476 |
| SARS_CoV_Rs_672/2006/1-1241 | VPI----E---------------------------------------------------- | 476 |
| Bat_SL_CoV_Rs4081/1-1241 | VPI----E---------------------------------------------------- | 476 |
| Bat_SL_CoV_Rs4255/1-1241 | VPI----E---------------------------------------------------- | 476 |
| Bat_SARS_CoV_Rm1/2004/1-1241 | IPV----E---------------------------------------------------- | 476 |
| Bat_SARS_CoV_HKU3-1/1-1242 | VPV----A---------------------------------------------------- | 477 |
| Bat_SARS_CoV_Rp3/1-1241 | VPV----A---------------------------------------------------- | 476 |
| Bat_SL_CoV_As6526/1-1241 | VPI----E---------------------------------------------------- | 476 |
| Bat_SL_CoV_Rs4237/1-1241 | VPI----E---------------------------------------------------- | 476 |
| Bat_SL_CoV_Rs4247/1-1242 | VPV----A---------------------------------------------------- | 477 |

| | | |
|---|---|---|
| HCoV_NL63/1-1356 | -HYTSYTIVGALYVTWSEG--NSITGVPYPVSG-----IREFSNLVLNNCTKYNIYDYVG | 637 |
| Felis_Catus_CoV_NDL/UU88/1-1467 | -KWRT--QFATITVSYKDG--AMITTMPKAQLG-----FQDISNIVKDECTDYNIYGFQG | 716 |
| Ferret_CoV/1-1438 | -RYFT-RVFAHLFVTYKYG--LDHLGVQTPDVG-----VKDLSVVYQNVCTEYNIYGHAG | 676 |
| Canine_CoV/1-1457 | -RTSE-QVVRSLYVIYEEG--DNIVGVPSDNSG-----LHDLSVLHLDSCTDYNIYGRTG | 704 |
| Mink_CoV_WD1133/1-1429 | -RYFT-GVFAHVYVSYKFG--LDIVGLPTSDAG-----LKDLSVLHLNVCTEYNVYGFSG | 673 |
| Mink_CoV_1/1-1439 | -RYFT-GVFAHVYVSYKFG--LDIVGLPKSDAG-----LKDLSVLHLNVCTEYNIYGFAG | 683 |
| Mink_CoV_WD1127/1-1438 | -RYFT-GVFAHVYVSYKFG--LDIVGLPRADAG-----LKDLSVLHLNVCTEYNVYGFAG | 682 |
| Rhinolophus_affinis_bat_CoV_HKU-2_related/1-1119 | WAWND-RSDR----IYDWW-IADFVSSGAYVCQ------ANPRAPQVGVCITYTIEKTTF | 417 |
| Rhinolophus_bat_CoV_HKU2/1-1128 | WSWND-YQDS----IYDWW-IADFVSTGAFVCE------KNPDAPRTGVCITYTIEKVTF | 419 |

```
HCoV_OC43/1-1361                 WSADS-CLQGDKCNIFANF--ILHDVNSGLTCSTD--LQKANTDIILGVCVNYDLYGILG 639
HCoV_HKU1/1-1356                 WSYDT-CVSNNRCNIFSNF--ILNGINSGTTCSND--LLQPNTEVFTDVCVDYDLYGITG 631
MERS_CoV/1-1353                  -------VAMTEQLQMGFGITVQYGTDTNSVCPKLEFANDTKIASQLGNCVEYSLYGVSG 613
Bat_SL_CoV_ZC45/1-1246           -------YQATRVVVLSFELLNA----PATVCG-----PKLSTQLVKNQCVNFNFNGLKG 525
Bat_SL_CoV_ZXC21/1-1245          -------YQATRVVVLSFELLNA----PATVCG-----PKLSTQLVKNQCVNFNFNGLKG 524
Pangolin_CoV_MP789/1-1265        -------YQPYRVVVLSFELLKA----PATVCG-----PKQSTNLVKNKCVNFNFNGLTG 544
Bat_CoV_RaTG13/1-1269            -------HQPYRVVVLSFELLNA----PATVCG-----PKKSTNLVKNKCVNFNFNGLTG 548
SARS-CoV2/1-1273                 -------YQPYRVVVLSFELLHA----PATVCG-----PKKSTNLVKNKCVNFNFNGLTG 548
Bat_SL_CoV_Rs4084/1-1256         -------HQPYRVVVLSFELLNA----PATVCG-----PKLSTDLIKNQCVNFNFNGLTG 535
Bat_SL_CoV_RsSHC014/1-1256       -------HQPYRVVVLSFELLNA----PATVCG-----PKLSTDLIKNQCVNFNFNGLTG 535
Bat_SL_CoV_Rs7327/1-1256         -------YQPYRVVVLSFELLNA----PATVCG-----PKLSTDLIKNQCVNFNFNGLTG 535
Bat_SL_CoV_Rs9401/1-1256         -------YQPYRVVVLSFELLNA----PATVCG-----PKLSTDLIKNQCVNFNFNGLTG 535
Bat_SL_CoV_WIV1/1-1256           -------YQPYRVVVLSFELLNA----PATVCG-----PKLSTDLIKNQCVNFNFNGLTG 535
Bat_SL_CoV_Rs3367/1-1256         -------YQPYRVVVLSFELLNA----PATVCG-----PKLSTDLIKNQCVNFNFNGLTG 535
Bat_SL_CoV_Rs4231/1-1255         -------HQPYRVVVLSFELLNA----PATVCG-----PKLSTDLIKNQCVNFNFNGLTG 534
Bat_SL_CoV_Rs4874/1-1255         -------YQPYRVVVLSFELLNA----PATVCG-----PKLSTDLIKNQCVNFNFNGLTG 534
SL_CoV_WIV16/1-1255              -------YQPYRVVVLSFELLNA----PATVCG-----PKLSTDLIKNQCVNFNFNGLTG 534
SARS_CoV_civet010/1-1255         -------YQPYRVVVLSFELLNA----PATVCG-----PKLSTDLIKNQCVNFNFNGLTG 534
SARS_CoV_Tor2/1-1255             -------YQPYRVVVLSFELLNA----PATVCG-----PKLSTDLIKNQCVNFNFNGLTG 534
Bat_SL_CoV__Rf4092/1-1234        -------YQATRVVVLSFELLNA----PATVCG-----PKLSTQLVKNQCVNFNFNGLKG 513
BtRf_CoV_JL2012/1-1236           -------YQATRVVVLSFELLNA----PATVCG-----PKLSTPLVKNQCVNFNFNGLKG 515
BtRs_YN2013/1-1233               -------YQATRVVVLSFELLNA----PATVCG-----PKLSTELVKNQCVNFNFNGLKG 512
Bat_CoV_Rp/Shaanxi2011/1-1240    -------YQATRVVVLSFELLNA----PATVCG-----PKLSTTLVKNQCVNFNFNGLKG 519
Bat_SARS_CoV_Rf1/2004/1-1241     -------YQATRVVVLSFELLNA----PATVCG-----PKLSTSLVKNQCVNFNFNGFKG 520
Bat_CoV/Yunnan2011/1-1241        -------YQATRVVVLSFELLNA----PATVCG-----PKLSTSLIKNQCVNFNFNGLKG 520
SARS_CoV_Rs_672/2006/1-1241      -------YQATRVVVLSFELLNA----PATVCG-----PKLSTGLVKNQCVNFNFNGLKG 520
Bat_SL_CoV_Rs4081/1-1241         -------YQATRVVVLSFELLNA----PATVCG-----PKLSTALVKNQCVNFNFNGLKG 520
Bat_SL_CoV_Rs4255/1-1241         -------YQATRVVVLSFELLNA----PATVCG-----PKLSTGLVKNQCVNFNFNGLKG 520
Bat_SARS_CoV_Rm1/2004/1-1241     -------YQATRVVVLSFELLNA----PATVCG-----PKLSTQLVKNQCVNFNFNGLRG 520
Bat_SARS_CoV_HKU3-1/1-1242       -------YQATRVVVLSFELLNA----PATVCG-----PKLSTELVKNQCVNFNFNGLKG 521
Bat_SARS_CoV_Rp3/1-1241          -------YQATRVVVLSFELLNA----PATVCG-----PKLSTQLVKNQCVNFNFNGLKG 520
Bat_SL_CoV_As6526/1-1241         -------YQATRVVVLSFELLNA----PATVCG-----PKLSTGLVKNQCVNFNFNGLKG 520
Bat_SL_CoV_Rs4237/1-1241         -------YQATRVVVLSFELLNA----PATVCG-----PKLSTGLVKNQCVNFNFNGLKG 520
Bat_SL_CoV_Rs4247/1-1242         -------YQATRVVVLSFELLNA----PATVCG-----PKLSTQLVKNQCVNFNFNGLKG 521
                                                                             . *  : .


HCoV_NL63/1-1356                 TGIIRSSNQS-LAGGITYVSN-SGNLLGFKNVSTGNIFIVTPCNQPDQVAVYQQSIIGAM 695
Felis_Catus_CoV_NDL/UU88/1-1467  TGIIRNTTSR-LVAGLYYTSI-SGDLLAFKNSTTGEIFTVVPCDLTAQAAVINDEIVGVI 774
Ferret_CoV/1-1438                VGIIRSTNQT-LLGGLYYTSL-SGDLLGFKNVTTGEVYSIVPCQLSAQAAVINGKIVGAV 734
Canine_CoV/1-1457                VGIIRQTNST-ILSGLYYTSL-SGDLLGFKNVSDGVVYSVTPCDVSAQAAVIDGAIVGAM 762
```

```
Mink_CoV_WD1133/1-1429                              TGIIRQTNQS-IPGGLYYTSL-SGDLLGFKNVTTGTVYSITPCEVSAQAAVIDGSIVGAI 731
Mink_CoV_1/1-1439                                   TGIIRVTNQT-VLGGLYYTSL-SGDLLGFKNVTTGTVYSITPCEVSAQAAVIDGSIVGAI 741
Mink_CoV_WD1127/1-1438                              TGIIRETNQT-VMGGLYYTSL-SGDLLGFKNVTTGTVYSITPCELSAQAAVIDGSIVGAI 740
Rhinolophus_affinis_bat_CoV_HKU-2_related/1-1119    QGILHYSNYS-FSQFYNMLYVGSQLRY---VRILGQVYEVQSCFQASYDVLYSS---AST 470
Rhinolophus_bat_CoV_HKU2/1-1128                     QGVLYESNFT-FAQYYNVLYFGSQLKY---VRILGKVYEVAPCFEASYDVLFRS---SSS 472
HCoV_OC43/1-1361                                    QGIFVEVNATYYNSWQNLLYDSNGNLYGFRDYITNRTFMIRSCYSGRVSAAFHAN--SSE 697
HCoV_HKU1/1-1356                                    QGIFKEVSAVYYNSWQNLLYDSNGNIIGFKDFVTNKTYNIFPCYAGRVSAAFHQN--ASS 689
MERS_CoV/1-1353                                     RGVFQNCTAV-GVRQQRFVYDAYQNLVGYYSD-DGNYYCLRACVSVPVSVIYDKE--TKT 669
Bat_SL_CoV_ZC45/1-1246                              TGVLTDSSKR-FQSFQQFGKDASDFIDSVRDPQTLEILDITPCSFGGVSVITPGTNTSLE 584
Bat_SL_CoV_ZXC21/1-1245                             TGVLTDSSKR-FQSFQQFGKDASDFIDSVRDPQTLEILDITPCSFGGVSVITPGTNTSSE 583
Pangolin_CoV_MP789/1-1265                           TGVLTESSKK-FLPFQQFGRDIADTTDAVRDPQTLEILDITPCSFGGVSVITPGTNTSNQ 603
Bat_CoV_RaTG13/1-1269                               TGVLTESNKK-FLPFQQFGRDIADTTDAVRDPQTLEILDITPCSFGGVSVITPGTNASNQ 607
SARS-CoV2/1-1273                                    TGVLTESNKK-FLPFQQFGRDIADTTDAVRDPQTLEILDITPCSFGGVSVITPGTNTSNQ 607
Bat_SL_CoV_Rs4084/1-1256                            TGVLTPSSKR-FQPFQQFGRDVSDFTDSVRDPKTSEILDISPCSFGGVSVITPGTNTSSE 594
Bat_SL_CoV_RsSHC014/1-1256                          TGVLTPSSKR-FQPFQQFGRDVSDFTDSVRDPKTSEILDISPCSFGGVSVITPGTNTSSE 594
Bat_SL_CoV_Rs7327/1-1256                            TGVLTSSSKR-FQPFQQFGRDVSDFTDSVRDPKTSEILDISPCSFGGVSVITPGTNTSSE 594
Bat_SL_CoV_Rs9401/1-1256                            TGVLTSSSKR-FQPFQQFGRDVSDFTDSVRDPKTSEILDISPCSFGGVSVITPGTNTSSE 594
Bat_SL_CoV_WIV1/1-1256                              TGVLTPSSKR-FQPFQQFGRDVSDFTDSVRDPKTSEILDISPCSFGGVSVITPGTNTSSE 594
Bat_SL_CoV_Rs3367/1-1256                            TGVLTPSSKR-FQPFQQFGRDVSDFTDSVRDPKTSEILDISPCSFGGVSVITPGTNTSSE 594
Bat_SL_CoV_Rs4231/1-1255                            TGVLTSSSKR-FQPFQQFGRDVSDFTDSVRDPKTSEILDISPCSFGGVSVITPGTNTSSE 593
Bat_SL_CoV_Rs4874/1-1255                            TGVLTPSSKR-FQPFQQFGRDVSDFTDSVRDPKTSEILDISPCSFGGVSVITPGTNTSSE 593
SL_CoV_WIV16/1-1255                                 TGVLTPSSKR-FQPFQQFGRDVLDFTDSVRDPKTSEILDISPCSFGGVSVITPGTNTSSE 593
SARS_CoV_civet010/1-1255                            TGVLTPSSKR-FQPFQQFGRDVSDFTDSVRDPKTSEILDISPCSFGGVSVITPGTNASSE 593
SARS_CoV_Tor2/1-1255                                TGVLTPSSKR-FQPFQQFGRDVSDFTDSVRDPKTSEILDISPCAFGGVSVITPGTNASSE 593
Bat_SL_CoV__Rf4092/1-1234                           TGVLTDSSKR-FQSFQQFGRDTSDFTDSVRDPQTLDILDITPCSFGGVSVITPGTNASSE 572
BtRf_CoV_JL2012/1-1236                              TGVLTDSSKT-FQSFQQFGRDASDFTDSVRDPQTLQILDISPCSFGGVSVITPGTNTSSA 574
BtRs_YN2013/1-1233                                  TGVLTSSSKR-FQSFQQFGRDASDFTDSVRDPQTLEILDITPCSFGGVSVITPGTNASSE 571
Bat_CoV_Rp/Shaanxi2011/1-1240                       TGVLTASSKK-FQSFQQFGRDASDFTDSVRDPQTLEILDISPCSFGGVSVITPGTNASTE 578
Bat_SARS_CoV_Rf1/2004/1-1241                        TGVLTDSSKT-FQSFQQFGRDASDFTDSVRDPQTLRILDISPCSFGGVSVITPGTNTSSA 579
Bat_CoV/Yunnan2011/1-1241                           TGVLTDSSKK-FQSFQQFGRDASDFTDSVRDPQTLQILDISPCSFGGVSVITPGTNASSE 579
SARS_CoV_Rs_672/2006/1-1241                         TGVLTDSSKR-FQSFQQFGRDTSDFTDSVRDPQTLQILDITPCSFGGVSVITPGTNASSE 579
Bat_SL_CoV_Rs4081/1-1241                            IGVLTDSSKR-FQSFQQFGRDTSDFTDSVRDPQTLQILDITPCSFGGVSVITPGTNASSE 579
Bat_SL_CoV_Rs4255/1-1241                            TGVLTDSSKR-FQSFQQFGRDTSDFTDSVRDPQTLQILDITPCSFGGVSVITPGTNASSE 579
Bat_SARS_CoV_Rm1/2004/1-1241                        TGVLTTSSKR-FQSFQQFGRDTSDFTDSVRDPQTLEILDISPCSFGGVSVITPGTNASSE 579
Bat_SARS_CoV_HKU3-1/1-1242                          TGVLTSSSKR-FQSFQQFGRDTSDFTDSVRDPQTLEILDISPCSFGGVSVITPGTNASSE 580
Bat_SARS_CoV_Rp3/1-1241                             TGVLTESSKR-FQSFQQFGRDTSDFTDSVRDPQTLEILDISPCSFGGVSVITPGTNASSE 579
Bat_SL_CoV_As6526/1-1241                            TGVLTDSSKR-FQSFQQFGRDTSDFTDSVRDPQTLQVLDITPCSFGGVSVITPGTNASSE 579
Bat_SL_CoV_Rs4237/1-1241                            TGVLTDSSKR-FQSFQQFGRDMSDFTDSVRDPQTLQILDITPCSFGGVSVITPGTNASSE 579
Bat_SL_CoV_Rs4247/1-1242                            TGVLTDSSKR-FQSFQQFGRDTSDFTDSVRDPQTLEILDITPCSFGGVSVITPGTNASSE 580
                                                     *::   .                               :  *      .
```

| | | |
|---|---|---|
| HCoV_NL63/1-1356 | TAVNESRYG--L-Q------NLLQLPNFYYVSNGGN---------------------NCT | 725 |
| Felis_Catus_CoV_NDL/UU88/1-1467 | TAINQTDLFEFV-N------HTQSRRSRSSTSETVTTYTMPQFYYITKWNNDT--LSNC- | 824 |
| Ferret_CoV/1-1438 | TSVQSPILD--L-P------HHIVTPQFYYHSIYNYSATPTS-YRTNNGFDKY--LVNC- | 781 |
| Canine_CoV/1-1457 | TSINSELLG--L-T------HWTTTPNFYYYSIYNYTNERTR----GTAIDSN--DVDC- | 806 |
| Mink_CoV_WD1133/1-1429 | TSVNSELLG--L-K------NHITTPYFYYYSVYNYTADNVT----R-SMQKY--DVNC- | 774 |
| Mink_CoV_1/1-1439 | TSVNSELLG--L-K------NHITTPYFYYYSVYNYTADSNA----R-SMQKY--DVNC- | 784 |
| Mink_CoV_WD1127/1-1438 | TSVNSELLG--L-K------NHIVTPYFYYYSVYNYTADNNS----R-SMQKY--DVNC- | 783 |
| Rhinolophus_affinis_bat_CoV_HKU-2_related/1-1119 | FGVLYRSFDCSHLYAFASR-T-------RSFVPRSGGIATALGCLFNAAYLPNDTMINCS | 522 |
| Rhinolophus_bat_CoV_HKU2/1-1128 | FGLLYRSFDCNQLRISASRFA-------ERLLPSHNGTATALGCLFNATYAPNDTMVNCT | 525 |
| HCoV_OC43/1-1361 | PALLFRNIKCNYVFNNSLTR----------QLQPINYFDSYLGCVVNAYNSTAISVQTCD | 747 |
| HCoV_HKU1/1-1356 | LALLYRNLKCSYVLNNI-------------SLTTQPYFDSYLGCVFNADNLTDYSVSSCA | 736 |
| MERS_CoV/1-1353 | HATLFGSVACEHISSTMSQYSRSTRSMLKRRDSTYGPLQTPVGCVLGLVNSSLF-VEDCK | 728 |
| Bat_SL_CoV_ZC45/1-1246 | VAVLYQDVNCTDVPTTIHADQ--LTPAWRIYATGTNVFQTQAGCLIGAEHVNA--SYECD | 640 |
| Bat_SL_CoV_ZXC21/1-1245 | VAVLYQDVNCTDVPTTIHADQ--LTPAWRIYAIGTSVFQTQAGCLIGAEHVNA--SYECD | 639 |
| Pangolin_CoV_MP789/1-1265 | VAVLYQDVNCTEVPVAIHADQ--LTPTWRVYSTGSNVFQTRAGCLIGAEHVNN--TYECD | 659 |
| Bat_CoV_RaTG13/1-1269 | VAVLYQDVNCTEVPVAIHADQ--LTPTWRVYSTGSNVFQTRAGCLIGAEHVNN--SYECD | 663 |
| SARS-CoV2/1-1273 | VAVLYQDVNCTEVPVAIHADQ--LTPTWRVYSTGSNVFQTRAGCLIGAEHVNN--SYECD | 663 |
| Bat_SL_CoV_Rs4084/1-1256 | VAVLYQDVNCTDVPVAIHADQ--LTPSWRVYSTGNNVFQTQAGCLIGAEHVDT--SYECD | 650 |
| Bat_SL_CoV_RsSHC014/1-1256 | VAVLYQDVNCTDVPVAIHADQ--LTPSWRVYSTGNNVFQTQAGCLIGAEHVDT--SYECD | 650 |
| Bat_SL_CoV_Rs7327/1-1256 | VAVLYQDVNCTDVPVAIHADQ--LTPAWRIYSTGNNVFQTQAGCLIGAEHVDT--SYECD | 650 |
| Bat_SL_CoV_Rs9401/1-1256 | VAVLYQDVNCTDVPVAIHADQ--LTPAWRIYSTGNNVFQTQAGCLIGAEHVDT--SYECD | 650 |
| Bat_SL_CoV_WIV1/1-1256 | VAVLYQDVNCTDVPVAIHADQ--LTPSWRVHSTGNNVFQTQAGCLIGAEHVDT--SYECD | 650 |
| Bat_SL_CoV_Rs3367/1-1256 | VAVLYQDVNCTDVPVAIHADQ--LTPSWRVYSTGNNVFQTQAGCLIGAEHVDT--SYECD | 650 |
| Bat_SL_CoV_Rs4231/1-1255 | VAVLYQDVNCTDVPVAIHADQ--LTPAWRIYSTGNNVFQTQAGCLIGAEHVDT--SYECD | 649 |
| Bat_SL_CoV_Rs4874/1-1255 | VAVLYQDVNCTDVPVAIHADQ--LTPSWRVYSTGNNVFQTQAGCLIGAEHVDT--SYECD | 649 |
| SL_CoV_WIV16/1-1255 | VAVLYQDVNCTDVPVAIHADQ--LTPSWRVYSTGNNVFQTQAGCLIGAEHVDT--SYECD | 649 |
| SARS_CoV_civet010/1-1255 | VAVLYQDVNCTDVSTLIHAEQ--LTPAWRIYSTGNNVFQTQAGCLIGAEHVDT--SYECD | 649 |
| SARS_CoV_Tor2/1-1255 | VAVLYQDVNCTDVSTAIHADQ--LTPAWRIYSTGNNVFQTQAGCLIGAEHVDT--SYECD | 649 |
| Bat_SL_CoV__Rf4092/1-1234 | VAVLYQDVNCTDVPTVIRADQ--LTPAWRVYSTGVNVFQTQAGCLIGAEHVNA--SYECD | 628 |
| BtRf_CoV_JL2012/1-1236 | VAVLYQDVNCTDVPTTIHADH--LTHSWRVYTTGPYVFQTQAGCLIGAEHVNA--SYQCD | 630 |
| BtRs_YN2013/1-1233 | VAVLYQDVNCTDVPTAIRADQ--LTPAWRVYSTGVNVFQTQAGCLIGAEHVNA--SYECD | 627 |
| Bat_CoV_Rp/Shaanxi2011/1-1240 | VAVLYQDVNCTDVPTAINADQ--LTPAWRVYSTGINVFQTQAGCLIGAEHVNA--SYECD | 634 |
| Bat_SARS_CoV_Rf1/2004/1-1241 | VAVLYQDVNCTDVPRTIQADQ--LAPSWRVYTTGPYVFQTQAGCLIGAEHVNA--SYQCD | 635 |
| Bat_CoV/Yunnan2011/1-1241 | VAVLYQDVNCTDVPTAIRADQ--LTPAWRVYSAGVNVFQTQAGCLIGAEHVNA--SYECD | 635 |
| SARS_CoV_Rs_672/2006/1-1241 | VAVLYQDVNCTDVPTAIRADQ--LTPAWRVYSTGVNVFQTQAGCLIGAEHVNA--SYECD | 635 |
| Bat_SL_CoV_Rs4081/1-1241 | VAVLYQDVNCTDVPTAIRADQ--LTPAWRVYSTGINVFQTQAGCLIGAEHVNA--SYECD | 635 |
| Bat_SL_CoV_Rs4255/1-1241 | VAVLYQDVNCTDVPTAIRADQ--LTPAWRIYSTGINVFQTQAGCLIGAEHVNA--SYECD | 635 |
| Bat_SARS_CoV_Rm1/2004/1-1241 | VAVLYQDVNCTDVPTSIHADQ--LTPAWRVYSTGVNVFQTQAGCLIGAEHVNA--SYECD | 635 |
| Bat_SARS_CoV_HKU3-1/1-1242 | VAVLYQDVNCTDVPTAIRADQ--LTPAWRVYSTGVNVFQTQAGCLIGAEHVNA--SYECD | 636 |

```
Bat_SARS_CoV_Rp3/1-1241                              VAVLYQDVNCTDVPAAIHADQ--LTPAWRVYSTGTNVFQTQAGCLIGAEHVNA--SYECD 635
Bat_SL_CoV_As6526/1-1241                             VAVLYQDVNCTDVPTAIRADQ--LTPAWRVYSTGVNVFQTQAGCLIGAEHVNA--SYECD 635
Bat_SL_CoV_Rs4237/1-1241                             VAVLYQDVNCTDVPTAIRADQ--LTPAWRVYSTGINVFQTQAGCLIGAEHVNA--SYECD 635
Bat_SL_CoV_Rs4247/1-1242                             VAVLYQDVNCTDVPTAIRADQ--LTPAWRVYSTGINVFQTQAGCLIGAEHVNA--SYECD 636
                                                      .                                                       *

HCoV_NL63/1-1356                                     TAVMTYSNFGICADGSLIPVRPRN---------------------------SSDNGISA- 757
Felis_Catus_CoV_NDL/UU88/1-1467                      TSVITYSSFAICNTGEIKYVNVTKVEIVD----------------------DSIGVIKP- 861
Ferret_CoV/1-1438                                    TPIITYSNMGVCENGALVFINITQ----------------------------SENPVQP- 812
Canine_CoV/1-1457                                    EPIITYSNIGVCKNGALVFINVTH----------------------------SDGDVQP- 837
Mink_CoV_WD1133/1-1429                               TPVISYSNMGVCANGALVFINVTH----------------------------TNGDVQP- 805
Mink_CoV_1/1-1439                                    TPVISYSNMGVCANGALVFINVTH----------------------------TNGDVQP- 815
Mink_CoV_WD1127/1-1438                               TPVISYSNMGVCANGALVFINVTH----------------------------TNGDVQP- 814
Rhinolophus_affinis_bat_CoV_HKU-2_related/1-1119     TPL----GAGFCADLGRGVSVRRIAFERH-----------------------DTTYVAPV 555
Rhinolophus_bat_CoV_HKU2/1-1128                      NPL----GDGFCADLLSNVVVRRMTFEKH-----------------------DTTYVAPV 558
HCoV_OC43/1-1361                                     LTV----GSGYCVDYSKNRRSR-----GA------ITTGYRFTNFEPFTVNSVNDSLEPV 792
HCoV_HKU1/1-1356                                     LRM----GSGFCVDYNSPSSSSSRRKRRS------ISASYRFVTFEPFNVSFVNDSIESV 786
MERS_CoV/1-1353                                      LPL----GQSLCALPDTPSTLTPRSVRSVPGEMRLASIAFNHPIQVDQLN-SS------- 776
Bat_SL_CoV_ZC45/1-1246                               IPI----GAGICASYHTASI----LRSTS----QKAIVAYTMSLGAENSIAYA------- 681
Bat_SL_CoV_ZXC21/1-1245                              IPI----GAGICASYHTASI----LRSTG----QKAIVAYTMSLGAENSIAYA------- 680
Pangolin_CoV_MP789/1-1265                            IPI----GAGICASYQTQTN----SRSVS----SQAIIAYTMSLGAENSVAYA------- 700
Bat_CoV_RaTG13/1-1269                                IPI----GAGICASYQTQTN----SRSVA----SQSIIAYTMSLGAENSVAYS------- 704
SARS-CoV2/1-1273                                     IPI----GAGICASYQTQTNSPRRARSVA----SQSIIAYTMSLGAENSVAYS------- 708
Bat_SL_CoV_Rs4084/1-1256                             IPI----GAGICASYHTVSS----LRSTS----QKSIVAYTMSLGADSSIAYS------- 691
Bat_SL_CoV_RsSHC014/1-1256                           IPI----GAGICASYHTVSS----LRSTS----QKSIVAYTMSLGADSSIAYS------- 691
Bat_SL_CoV_Rs7327/1-1256                             IPI----GAGICASYHTVSS----LRSTS----QKSIVAYTMSLGADSSIVYS------- 691
Bat_SL_CoV_Rs9401/1-1256                             IPI----GAGICASYHTVSS----LRSTS----QKSIVAYTMSLGADSSIVYS------- 691
Bat_SL_CoV_WIV1/1-1256                               IPI----GAGICASYHTVSS----LRSTS----QKSIVAYTMSLGADSSIAYS------- 691
Bat_SL_CoV_Rs3367/1-1256                             IPI----GAGICASYHTVSS----LRSTS----QKSIVAYTMSLGADSSIAYS------- 691
Bat_SL_CoV_Rs4231/1-1255                             IPI----GAGICASYHTVSS----LRSTS----QKSIVAYTMSLGADSSIAYS------- 690
Bat_SL_CoV_Rs4874/1-1255                             IPI----GAGICASYHTVSS----LRSTS----QKSIVAYTMSLGADSSIAYS------- 690
SL_CoV_WIV16/1-1255                                  IPI----GAGICASYHTVSS----LRSTS----QKSIVAYTMSLGADSSIAYS------- 690
SARS_CoV_civet010/1-1255                             IPI----GAGICASYHTVSS----LRSTS----QKSIVAYTMSLGADSSIAYS------- 690
SARS_CoV_Tor2/1-1255                                 IPI----GAGICASYHTVSL----LRSTS----QKSIVAYTMSLGADSSIAYS------- 690
Bat_SL_CoV__Rf4092/1-1234                            IPI----GAGICASYHTAST----LRGVG----QKSIVAYTMSLGAENSIAYA------- 669
BtRf_CoV_JL2012/1-1236                               IPI----GAGICASYHTASL----LRSTG----QKSIVAYTMSLGAENSVAYA------- 671
BtRs_YN2013/1-1233                                   IPI----GAGICASYHTAST----LRSIG----QKSIVAYTMSLGAENSIAYA------- 668
Bat_CoV_Rp/Shaanxi2011/1-1240                        IPI----GAGICASYHTASV----LRSTG----QKSIVAYTMSLGAENSIAYA------- 675
Bat_SARS_CoV_Rf1/2004/1-1241                         IPI----GAGICASYHTASH----LRSTG----QKSIVAYTMSLGAENSVAYA------- 676
Bat_CoV/Yunnan2011/1-1241                            IPI----GAGICASYHTASL----LRNTG----QKSIVAYTMSLGAENSIAYA------- 676
```

```
SARS_CoV_Rs_672/2006/1-1241                          IPI----GAGICASYHTAST----LRSVG----QKSIVAYTMSLGAENSIAYA------- 676
Bat_SL_CoV_Rs4081/1-1241                             IPI----GAGICASYHTAST----LRSVG----QKSIVAYTMSLGAENSIAYA------- 676
Bat_SL_CoV_Rs4255/1-1241                             IPI----GAGICASYHTAST----LRSVG----QKSIVAYTMSLGAENSIAYA------- 676
Bat_SARS_CoV_Rm1/2004/1-1241                         IPI----GAGICASYHTASV----LRSTG----QKSIVAYTMSLGAENSIAYA------- 676
Bat_SARS_CoV_HKU3-1/1-1242                           IPI----GAGICASYHTASV----LRSTG----QKSIVAYTMSLGAENSIAYA------- 677
Bat_SARS_CoV_Rp3/1-1241                              IPI----GAGICASYHTAST----LRSVG----QKSIVAYTMSLGAENSIAYA------- 676
Bat_SL_CoV_As6526/1-1241                             IPI----GAGICASYHTAST----LRSVG----QKSIVAYTMSLGAENSIAYA------- 676
Bat_SL_CoV_Rs4237/1-1241                             IPI----GAGICASYHTAST----LRSVG----QKSIVAYTMSLGAENSIAYA------- 676
Bat_SL_CoV_Rs4247/1-1242                             IPI----GAGICASYHTAST----LRSVG----QKSIVAYTMSLGAENSIAYA------- 677
                                                       :    . . *

HCoV_NL63/1-1356                                     IITANLSIPSNWTTSVQVEYLQITSTPIVVDCATYVCNGNPRCKNLLKQYTSACKTIEDA 817
Felis_Catus_CoV_NDL/UU88/1-1467                      VSTGNISIPKNFTVAVQAEYIQIQVKPVVVDCAKYVCNGNRHCLSLLTQYTSACQTIENA 921
Ferret_CoV/1-1438                                    ISTGNVTIPSNFTISVQVEYLQMSSEAVSIDCAQYVCNGNPRCNRLLAQYISACHAIEQA 872
Canine_CoV/1-1457                                    ISTGNVTIPTNFTISVQVEYIQVYTTPVSIDCSRYVCNGNPRCNKLLTQYVSACQTIEQA 897
Mink_CoV_WD1133/1-1429                               ISTGNVTIPSNFTISVQMEYVQVSTELVSIDCARYVCNGNARCGKLLSQYVSACHTIEQA 865
Mink_CoV_1/1-1439                                    ISTGNVSIPSNFTISVQMEYVQVSTELVSIDCARYVCNGNARCGRLLSQYVSACHTIEQA 875
Mink_CoV_WD1127/1-1438                               ISTGNVTIPSNFTISVQMEYVQVSTELVSIDCARYVCNGNARCGRLLSQYVSACQTIEQA 874
Rhinolophus_affinis_bat_CoV_HKU-2_related/1-1119     INERYIELATSHQLIMTEQFLQTSMPKFSVSCEAYICDVSKSCKNLLFRYGGFCQKIESD 615
Rhinolophus_bat_CoV_HKU2/1-1128                      TNERFTELPLDHQLVLTEQFLQTTMPKFSISCETYICDVSKACKNLLFRYGGFCQKIEAD 618
HCoV_OC43/1-1361                                     GGLYEIQIPSEFTIGNMEEFIQTSSPKVTIDCAAFVCGDYAACKSQLVEYGSFCDNINAI 852
HCoV_HKU1/1-1356                                     GGLYEIKIPTNFTIVGQEEFIQTNSPKVTIDCSLFVCSNYAACHDLLSEYGTFCDNINSI 846
MERS_CoV/1-1353                                      --YFKLSIPTNFSFGVTQEYIQTTIQKVTVDCKQYVCNGFQKCEQLLREYGQFCSKINQA 834
Bat_SL_CoV_ZC45/1-1246                               --NNSIAIPTNFSISVTTEVMPVSMAKTSVDCTMYICGDSIECSNLLLQYGSFCTQLNRA 739
Bat_SL_CoV_ZXC21/1-1245                              --NNSIAIPTNFSISVTTEVMPVSMAKTSVDCTMYICGDSIECSNLLLQYGSFCTQLNRA 738
Pangolin_CoV_MP789/1-1265                            --NNSIAIPTNFTISVTTEILPVSMTKTSVDCTMYICGDSIECSNLLLQYGSFCTQLNRA 758
Bat_CoV_RaTG13/1-1269                                --NNSIAIPTNFTISVTTEILPVSMTKTSVDCTMYICGDSTECSNLLLQYGSFCTQLNRA 762
SARS-CoV2/1-1273                                     --NNSIAIPTNFTISVTTEILPVSMTKTSVDCTMYICGDSTECSNLLLQYGSFCTQLNRA 766
Bat_SL_CoV_Rs4084/1-1256                             --NNTIAIPTNFSISITTEVMPVSMAKTSVDCNMYICGDSTECANLLLQYGSFCTQLNRA 749
Bat_SL_CoV_RsSHC014/1-1256                           --NNTIAIPTNFSISITTEVMPVSMAKTSVDCNMYICGDSTECANLLLQYGSFCTQLNRA 749
Bat_SL_CoV_Rs7327/1-1256                             --NNTIAIPTNFSISITTEVMPVSMAKTSVDCNMYICGDSTECANLLLQYGSFCTQLNRA 749
Bat_SL_CoV_Rs9401/1-1256                             --NNTIAIPTNFSISITTEVMPVSMAKTSVDCNMYICGDSTECANLLLQYGSFCTQLNRA 749
Bat_SL_CoV_WIV1/1-1256                               --NNTIAIPTNFSISITTEVMPVSMAKTSVDCNMYICGDSTECANLLLQYGSFCTQLNRA 749
Bat_SL_CoV_Rs3367/1-1256                             --NNTIAIPTNFSISITTEVMPVSMAKTSVDCNMYICGDSTECANLLLQYGSFCTQLNRA 749
Bat_SL_CoV_Rs4231/1-1255                             --NNTIAIPTNFSISITTEVMPVSMAKTSVDCNMYICGDSTECANLLLQYGSFCTQLNRA 748
Bat_SL_CoV_Rs4874/1-1255                             --NNTIAIPTNFSISITTEVMPVSMAKTSVDCNMYICGDSTECANLLLQYGSFCTQLNRA 748
SL_CoV_WIV16/1-1255                                  --NNTIAIPTNFSISITTEVMPVSMAKTSVDCNMYICGDSTECANLLLQYGSFCTQLNRA 748
SARS_CoV_civet010/1-1255                             --NNTIAIPTNFSISITTEVMPVSMAKTSVDCNMYICGDSTECANLLLQYGSFCRQLNRA 748
SARS_CoV_Tor2/1-1255                                 --NNTIAIPTNFSISITTEVMPVSMAKTSVDCNMYICGDSTECANLLLQYGSFCTQLNRA 748
Bat_SL_CoV__Rf4092/1-1234                            --NNSIAIPTNFSISVTTEVMPVSMAKTSVDCTMYICGDSQECSNLLLQYGSFCTQLNRA 727
```

```
BtRf_CoV_JL2012/1-1236                            --NNSIAIPTNFSISVTTEVMPVSMAKTSVDCTMYICGDSLECSNLLLQYGSFCTQLNRA 729
BtRs_YN2013/1-1233                                --NNSIAIPTNFSISVTTEVMPVSMAKTSVDCTMYICGDSQECSNLLLQYGSFCTQLNRA 726
Bat_CoV_Rp/Shaanxi2011/1-1240                     --NNSIAIPTNFSISVTTEVMPVSMAKTSVDCTMYICGDSLECSNLLLQYGSFCTQLNRA 733
Bat_SARS_CoV_Rf1/2004/1-1241                      --NNSIAIPTNFSISVTTEVMPVSMAKTSVDCTMYICGDSLECSNLLLQYGSFCTQLNRA 734
Bat_CoV/Yunnan2011/1-1241                         --NNSIAIPTNFSISVTTEVMPVSMAKTSVDCTMYICGDSQECSNLLLQYGSFCTQLNRA 734
SARS_CoV_Rs_672/2006/1-1241                       --NNSIAIPTNFSISVTTEVMPVSMAKTSVDCTMYICGDSQECSNLLLQYGSFCTQLNRA 734
Bat_SL_CoV_Rs4081/1-1241                          --NNSIAIPTNFSISVTTEVMPVSMSKTSVDCTMYICGDSQECSNLLLQYGSFCTQLNRA 734
Bat_SL_CoV_Rs4255/1-1241                          --NNSIAIPTNFSISVTTEVMPVSMSKTSVDCTMYICGDSQECSNLLLQYGSFCTQLNRA 734
Bat_SARS_CoV_Rm1/2004/1-1241                      --NNSIAIPTNFSISVTTEVMPVSIAKTSVDCTMYICGDSLECSNLLLQYGSFCTQLNRA 734
Bat_SARS_CoV_HKU3-1/1-1242                        --NNSIAIPTNFSISVTTEVMPVSMAKTAVDCTMYICGDSLECSNLLLQYGSFCTQLNRA 735
Bat_SARS_CoV_Rp3/1-1241                           --NNSIAIPTNFSISVTTEVMPVSMAKTSVDCTMYICGDSLECSNLLLQYGSFCTQLNRA 734
Bat_SL_CoV_As6526/1-1241                          --NNSIAIPTNFSISVTTEVMPVSMAKTSVDCTMYICGDSQECSNLLLQYGSFCTQLNRA 734
Bat_SL_CoV_Rs4237/1-1241                          --NNSIAIPTNFSISVTTEVMPVSMSKTSVDCTMYICGDSQECSNLLLQYGSFCTQLNRA 734
Bat_SL_CoV_Rs4247/1-1242                          --NNSIAIPTNFSISVTTEVMPVSMSKTSVDCTMYICGDSQECSNLLLQYGSFCTQLNRA 735
                                                         :  .       : :        :.*  ::*.    *   * .*   *  ::
```

```
HCoV_NL63/1-1356                                  LRLSAHLETNDVSSMLTFDSNAFSLANVTSFGDY--------------------NLSSVL 857
Felis_Catus_CoV_NDL/UU88/1-1467                   LNLGARLESLMLNDMVTVSDRSLELATVEKFNTTTL-------GGEKLGGFYFDGLRELL 974
Ferret_CoV/1-1438                                 LITSTRLEALELESMISISDNALALATVETFNSSDYLDPVYNDQHNTIGGIYMDGLKDLL 932
Canine_CoV/1-1457                                 LAMSARLENMEVDSMLFVSENALKLASVEAFNSTEHLDPIYKE-WPNIGGSWLGGLKDIL 956
Mink_CoV_WD1133/1-1429                            LAMGSRLESMELESMISISANALKLASVEEFNSSAMLNPVYNESGNTIGGIYLDGLKDIL 925
Mink_CoV_1/1-1439                                 LAMGSRLESMELESMISISEYALKLASVEEFNSSSMLNPIYNESGNTIGGIYLDGLKDIL 935
Mink_CoV_WD1127/1-1438                            LAMGSRLESMELESMISISENALKLASVEEFNSSAMLNPIYNESGNTIGGIYLDGLKDIL 934
Rhinolophus_affinis_bat_CoV_HKU-2_related/1-1119  IRGAGALLDNDVGELYSTLSAKT--SVSAFDTGPF-------------------NITQSF 654
Rhinolophus_bat_CoV_HKU2/1-1128                   IRGAGVLLDSDVSGLYSTIAAKT--SSITPTTDRF-------------------NVSQFF 657
HCoV_OC43/1-1361                                  LTEVNELLDTTQLQVANSLMNGVTLSTKLKDGVNF-------------------NVDDIN 893
HCoV_HKU1/1-1356                                  LDEVNGLLDTTQLHVADTLMQGVTLSSNLNTNLHF-------------------DVDNIN 887
MERS_CoV/1-1353                                   LHGANLRQDDSVRNLFASVKSSQSSPIIPGFGGDF-------------------NLTLLE 875
Bat_SL_CoV_ZC45/1-1246                            LSGIAIEQDKNTQEVFAQVKQIYKTPPIKDFGG-F-------------------NFSQIL 779
Bat_SL_CoV_ZXC21/1-1245                           LSGIAIEQDKNTQEVFAQVKQIYKTPPIKDFGG-F-------------------NFSQIL 778
Pangolin_CoV_MP789/1-1265                         LTGIAVEQDKNTQEVFAQVKQIYKTPPIKDFGG-F-------------------NFSQIL 798
Bat_CoV_RaTG13/1-1269                             LTGIAVEQDKNTQEVFAQVKQIYKTPPIKDFGG-F-------------------NFSQIL 802
SARS-CoV2/1-1273                                  LTGIAVEQDKNTQEVFAQVKQIYKTPPIKDFGG-F-------------------NFSQIL 806
Bat_SL_CoV_Rs4084/1-1256                          LSGIAVEQDRNTREVFAQVKQMYKTPTLKDFGG-F-------------------NFSQIL 789
Bat_SL_CoV_RsSHC014/1-1256                        LSGIAVEQDRNTREVFAQVKQMYKTPTLKDFGG-F-------------------NFSQIL 789
Bat_SL_CoV_Rs7327/1-1256                          LSGIAVEQDRNTREVFAQVKQMYKTPTLKDFGG-F-------------------NFSQIL 789
Bat_SL_CoV_Rs9401/1-1256                          LSGIAVEQDRNTREVFAQVKQMYKTPTLKDFGG-F-------------------NFSQIL 789
Bat_SL_CoV_WIV1/1-1256                            LSGIAVEQDRNTREVFAQVKQMYKTPTLKDFGG-F-------------------NFSQIL 789
Bat_SL_CoV_Rs3367/1-1256                          LSGIAVEQDRNTREVFAQVKQMYKTPTLKDFGG-F-------------------NFSQIL 789
Bat_SL_CoV_Rs4231/1-1255                          LSGIAVEQDRNTREVFAQVKQMYKTPALKDFGG-F-------------------NFSQIL 788
```

```
Bat_SL_CoV_Rs4874/1-1255                          LSGIAVEQDRNTREVFAQVKQMYKTPTLKDFGG-F-------------------NFSQIL 788
SL_CoV_WIV16/1-1255                               LSGIAVEQDRNTREVFAQVKQMYKTPTLKDFGG-F-------------------NFSQIL 788
SARS_CoV_civet010/1-1255                          LSGIAAEQDRNTREVFVQVKQMYKTPTLKDFGG-F-------------------NFSQIL 788
SARS_CoV_Tor2/1-1255                              LSGIAAEQDRNTREVFAQVKQMYKTPTLKYFGG-F-------------------NFSQIL 788
Bat_SL_CoV__Rf4092/1-1234                         LTGVALEQDKNTQDVFAQVKQMYKTPAIKDFGG-F-------------------NFSQIL 767
BtRf_CoV_JL2012/1-1236                            LSGIAVEQDKNTQEVFAQVKQMYKTPTIRDFGG-F-------------------NFSQIL 769
BtRs_YN2013/1-1233                                LTGVALEQDKNTQEVFAQVKQMYKTPAIKDFGG-F-------------------NFSQIL 766
Bat_CoV_Rp/Shaanxi2011/1-1240                     LTGIAIEQDKNTQEVFAQVKQMYKTPAIKDFGG-F-------------------NFSQIL 773
Bat_SARS_CoV_Rf1/2004/1-1241                      LSGIAVEQDKNTQEVFAQVKQMYKTPTIRDFGG-F-------------------NFSQIL 774
Bat_CoV/Yunnan2011/1-1241                         LSGIAVEQDKNTQEVFAQVKQMYKTPAIKDFGG-F-------------------NFSQIL 774
SARS_CoV_Rs_672/2006/1-1241                       LTGVALEQDKNTQEVFAQVKQMYKTPAIKDFGG-F-------------------NFSQIL 774
Bat_SL_CoV_Rs4081/1-1241                          LTGIAIEQDKNTQEVFAQVKQMYKTPAIKDFGG-F-------------------NFSQIL 774
Bat_SL_CoV_Rs4255/1-1241                          LTGIAIEQDKNTQEVFAQVKQMYKTPAIKDFGG-F-------------------NFSQIL 774
Bat_SARS_CoV_Rm1/2004/1-1241                      LTGIAIEQDKNTQEVFAQVKQMYKTPAIKDFGG-F-------------------NFSQIL 774
Bat_SARS_CoV_HKU3-1/1-1242                        LTGIAIEQDKNTQEVFAQVKQMYKTPAIKDFGG-F-------------------NFSQIL 775
Bat_SARS_CoV_Rp3/1-1241                           LSGIAIEQDKNTQEVFAQVKQMYKTPAIKDFGG-F-------------------NFSQIL 774
Bat_SL_CoV_As6526/1-1241                          LTGVALEQDKNTQEVFAQVKQMYKTPAIKDFGG-F-------------------NFSQIL 774
Bat_SL_CoV_Rs4237/1-1241                          LTGIAIEQDKNTQEVFAQVKQMYKTPAIKDFGG-F-------------------NFSQIL 774
Bat_SL_CoV_Rs4247/1-1242                          LTGIAIEQDKNTQEVFAQVKQMYKTPAIKDFGG-F-------------------NFSQIL 775
                                                  :             :                                       ..


HCoV_NL63/1-1356                                  PQR-----NIRSSRIAGRSALEDLLFSKVVTSGLGTVDVDYKSCT--KGLSIADLACAQY 910
Felis_Catus_CoV_NDL/UU88/1-1467                   PPT-----------IGKRSAVEDLLFNKVVTSGLGTVDDDYKKCS--AGTDVADLVCAQY 1021
Ferret_CoV/1-1438                                 PRR-----SCANKHGTCRSVIEELLFNKVVTSGLGTVDEDYKRCT--NGLDIADLVCAQY 985
Canine_CoV/1-1457                                 PSH--------NSKRKYRSAIEDLLFDKVVTSGLGTVDEDYKRCT--GGYDIADLVCAQY 1006
Mink_CoV_WD1133/1-1429                            PRK--------NKHGSSRSTIEDLLFNKVVTTGLGTVDEDYKRCT--KGSDIADLACAQY 975
Mink_CoV_1/1-1439                                 PRR--------NPHGSSRSTIEDLLFNKVVTTGLGTVDEDYKRCT--KGADIADLACAQY 985
Mink_CoV_WD1127/1-1438                            PRK--------NKHGSSRSTIEDLLFNKVVTTGLGTVDEDYKRCT--KGSDIADLACAQY 984
Rhinolophus_affinis_bat_CoV_HKU-2_related/1-1119  LPS----------ASGGRSVIEDLLFSKIETTGPGFY-NDYYNCK---KNAIQDLTCAQY 700
Rhinolophus_bat_CoV_HKU2/1-1128                   LPKVQ----SNSERFESRSVIEDLLFSKIETTGPGFY-GDYYNCK---KNAIQDLTCAQY 709
HCoV_OC43/1-1361                                  FSPVLGCLGSECSKASSRSAIEDLLFDKVKLSDVGFV-EAYNNCT--GGAEIRDLICVQS 950
HCoV_HKU1/1-1356                                  FKSLVGCLGPHCG-SSSRSFFEDLLFDKVKLSDVGFV-EAYNNCT--GGSEIRDLLCVQS 943
MERS_CoV/1-1353                                   PVSIS------TGSRSARSAIEDLLFDKVTIADPGYM-QGYDDCMQQGPASARDLICAQY 928
Bat_SL_CoV_ZC45/1-1246                            PDP---------SKPSKRSFIEDLLFNKVTLADAGFI-KQYGDCL--GGISARDLICAQK 827
Bat_SL_CoV_ZXC21/1-1245                           PDP---------SKPSKRSFIEDLLFNKVTLADAGFI-KQYGDCL--GDISARDLICAQK 826
Pangolin_CoV_MP789/1-1265                         PDP---------SKPSKRSFIEDLLFNKVTLADAGFI-KQYGDCL--GDIAARDLICAQK 846
Bat_CoV_RaTG13/1-1269                             PDP---------SKPSKRSFIEDLLFNKVTLADAGFI-KQYGDCL--GDIAARDLICAQK 850
SARS-CoV2/1-1273                                  PDP---------SKPSKRSFIEDLLFNKVTLADAGFI-KQYGDCL--GDIAARDLICAQK 854
Bat_SL_CoV_Rs4084/1-1256                          PDP---------LKPTKRSFIEDLLFNKVTLADAGFM-KQYGECL--GDINARDLICAQK 837
Bat_SL_CoV_RsSHC014/1-1256                        PDP---------LKPTKRSFIEDLLFNKVTLADAGFM-KQYGECL--GDINARDLICAQK 837
```

```
Bat_SL_CoV_Rs7327/1-1256                          PDP---------LKPTKRSFIEDLLFNKVTLADAGFM-KQYGECL--GDINARDLICAQK 837
Bat_SL_CoV_Rs9401/1-1256                          PDP---------LKPTKRSFIEDLLFNKVTLADAGFM-KQYGECL--GDINARDLICAQK 837
Bat_SL_CoV_WIV1/1-1256                            PDP---------LKPTKRSFIEDLLFNKVTLADAGFM-KQYGECL--GDINARDLICAQK 837
Bat_SL_CoV_Rs3367/1-1256                          PDP---------LKPTKRSFIEDLLFNKVTLADAGFM-KQYGECL--GDINARDLICAQK 837
Bat_SL_CoV_Rs4231/1-1255                          PDP---------LKPTKRSFIEDLLFNKVTLADAGFM-KQYGECL--GDVNARDLICAQK 836
Bat_SL_CoV_Rs4874/1-1255                          PDP---------LKPTKRSFIEDLLFNKVTLADAGFM-KQYGECL--GDINARDLICAQK 836
SL_CoV_WIV16/1-1255                               PDP---------LKPTKRSFIEDLLFNKVTLADAGFM-KQYGECL--GDINARDLICAQK 836
SARS_CoV_civet010/1-1255                          PDP---------LKPTKRSFIEDLLFNKVTLADAGFM-KQYGECL--GDINARDLICAQK 836
SARS_CoV_Tor2/1-1255                              PDP---------LKPTKRSFIEDLLFNKVTLADAGFM-KQYGECL--GDINARDLICAQK 836
Bat_SL_CoV__Rf4092/1-1234                         PDP---------SKPTKRSFIEDLLFNKVTLADAGFM-KQYGECL--GDISARDLICAQK 815
BtRf_CoV_JL2012/1-1236                            PDP---------LKPTKRSFIEDLLYNKVTLADAGFM-KQYADCL--GGINARDLICAQK 817
BtRs_YN2013/1-1233                                PDP---------SKPTKRSFIEDLLFNKVTLADAGFM-KQYGECL--GDISARDLICAQK 814
Bat_CoV_Rp/Shaanxi2011/1-1240                     PDP---------SKPTKRSFIEDLLFNKVTLADAGFM-KQYGECL--GDISARDLICAQK 821
Bat_SARS_CoV_Rf1/2004/1-1241                      PDP---------LKPTKRSFIEDLLYNKVTLADAGFM-KQYADCL--GGINARDLICAQK 822
Bat_CoV/Yunnan2011/1-1241                         PDP---------SKPTKRSFIEDLLFNKVTLADAGFM-KQYGECL--GDISARDLICAQK 822
SARS_CoV_Rs_672/2006/1-1241                       PDP---------SKPTKRSFIEDLLFNKVTLADAGFM-KQYGECL--GDISARDLICAQK 822
Bat_SL_CoV_Rs4081/1-1241                          PDP---------SKPTKRSFIEDLLFNKVTLADAGFM-KQYGECL--GDINARDLICAQK 822
Bat_SL_CoV_Rs4255/1-1241                          PDP---------SKPTKRSFIEDLLFNKVTLADAGFM-KQYGECL--GDINARDLICAQK 822
Bat_SARS_CoV_Rm1/2004/1-1241                      PDP---------SKPTKRSFIEDLLFNKVTLADAGFM-KQYGECL--GDISARDLICAQK 822
Bat_SARS_CoV_HKU3-1/1-1242                        PDP---------SKPTKRSFIEDLLFNKVTLADAGFM-KQYGDCL--GDVSARDLICAQK 823
Bat_SARS_CoV_Rp3/1-1241                           PDP---------SKPTKRSFIEDLLFNKVTLADAGFM-KQYGECL--GDISARDLICAQK 822
Bat_SL_CoV_As6526/1-1241                          PDP---------SKPTKRSFIEDLLFNKVTLADAGFM-KQYGECL--GDINARDLICAQK 822
Bat_SL_CoV_Rs4237/1-1241                          PDP---------SKPTKRSFIEDLLFNKVTLADAGFM-KQYGECL--GDINARDLICAQK 822
Bat_SL_CoV_Rs4247/1-1242                          PDP---------SKPTKRSFIEDLLFNKVTLADAGFM-KQYGECL--GDINARDLICAQK 823
                                                                   ** .*:**:.*:  :. *     *  *         ** *.*
```

```
HCoV_NL63/1-1356                                  YNGIMVLPGVADAERMAMYTGSLIGGMVLGGLT----SAAAIPFSLALQARLNYVALQTD 966
Felis_Catus_CoV_NDL/UU88/1-1467                   YNGIMVLPGVVDQNKMAMYTASLIGGMALGSIT----SAVAVPFAMQVQARLNYVALQTD 1077
Ferret_CoV/1-1438                                 YNGIMVLPGVVNADKMAMYTASLAGGITLGALGG---GLVSVPFATAVQARLNYVALQTD 1042
Canine_CoV/1-1457                                 YNGIMVLPGVANDDKMAMYTASLAGGITLGALGG---GAVSIPFAVAVQARLNYVALQTD 1063
Mink_CoV_WD1133/1-1429                            YNGIMVLPGVANDGKMSMYTASLSGGISLGALGG---GAVAIPFSLAVQARLNYVALQTD 1032
Mink_CoV_1/1-1439                                 YNGIMVLPGVANDGKMSMYTASLSGGISLGALGG---GAVAIPFSLAVQARLNYVALQTD 1042
Mink_CoV_WD1127/1-1438                            YNGIMVLPGVANDGKMSMYTASLSGGISLGALGG---GAVAIPFSLAVQARLNYVALQTD 1041
Rhinolophus_affinis_bat_CoV_HKU-2_related/1-1119  HNGILVIPPIMDAETLGMYGGIAAASLSLGIFGG---QAGIVTWSTAMAGRLNALGVVQN 757
Rhinolophus_bat_CoV_HKU2/1-1128                   HNGILVIPPVMDAETLGMYGGIAAASLTLGIFGG---QAGITTWSLAMAGRLNALGVVQN 766
HCoV_OC43/1-1361                                  YKGIKVLPPLLSENQISGYTLAATSASLFPLWTA----AAGVPFYLNVQYRINGLGVTMD 1006
HCoV_HKU1/1-1356                                  FNGIKVLPPILSESQISGYTTAATVAAMFPPWSA----AAGIPFSLNVQYRINGLGVTMD 999
MERS_CoV/1-1353                                   VAGYKVLPPLMDVNMEAAYTSSLLGSIAGVGWTAGLSSFAAIPFAQSIFYRLNGVGITQQ 988
Bat_SL_CoV_ZC45/1-1246                            FNGLTVLPPLLTDEMIAAYTAALISGTATAGWTFGAGAALQIPFAMQMAYRFNGIGVTQN 887
Bat_SL_CoV_ZXC21/1-1245                           FNGLTVLPPLLTDEMIAAYTAALISGTATAGWTFGAGAALQIPFAMQMAYRFNGIGVTQN 886
```

```
Pangolin_CoV_MP789/1-1265                               FNGLTVLPPLLTDEMIAQYTSALLAGTITSGWTFGAGAALQIPFAMQMAYRFNGIGVTQN 906
Bat_CoV_RaTG13/1-1269                                   FNGLTVLPPLLTDEMIAQYTSALLAGTITSGWTFGAGAALQIPFAMQMAYRFNGIGVTQN 910
SARS-CoV2/1-1273                                        FNGLTVLPPLLTDEMIAQYTSALLAGTITSGWTFGAGAALQIPFAMQMAYRFNGIGVTQN 914
Bat_SL_CoV_Rs4084/1-1256                                FNGLTVLPPLLTDDMIAAYTAALVSGTATAGWTFGAGAALQIPFAMQMAYRFNGIGVTQN 897
Bat_SL_CoV_RsSHC014/1-1256                              FNGLTVLPPLLTDDMIAAYTAALVSGTATAGWTFGAGAALQIPFAMQMAYRFNGIGVTQN 897
Bat_SL_CoV_Rs7327/1-1256                                FNGLTVLPPLLTDDMIAAYTAALVSGTATAGWTFGAGAALQIPFAMQMAYRFNGIGVTQN 897
Bat_SL_CoV_Rs9401/1-1256                                FNGLTVLPPLLTDDMIAAYTAALVSGTATAGWTFGAGAALQIPFAMQMAYRFNGIGVTQN 897
Bat_SL_CoV_WIV1/1-1256                                  FNGLTVLPPLLTDDMIAAYTAALVSGTATAGWTFGAGAALQIPFAMQMAYRFNGIGVTQN 897
Bat_SL_CoV_Rs3367/1-1256                                FNGLTVLPPLLTDDMIAAYTAALVSGTATAGWTFGAGAALQIPFAMQMAYRFNGIGVTQN 897
Bat_SL_CoV_Rs4231/1-1255                                FNGLTVLPPLLTDDMIAAYTAALVSGTATAGWTFGAGAALQIPFAMQMAYRFNGIGVTQN 896
Bat_SL_CoV_Rs4874/1-1255                                FNGLTVLPPLLTDDMIAAYTAALVSGTATAGWTFGAGAALQIPFAMQMAYRFNGIGVTQN 896
SL_CoV_WIV16/1-1255                                     FNGLTVLPPLLTDDMIAAYTAALVSGTATAGWTFGAGAALQIPFAMQMAYRFNGIGVTQN 896
SARS_CoV_civet010/1-1255                                FNGLTVLPPLLTDDMIAAYTAALVSGTATAGWTFGAGAALQIPFAMQMAYRFNGIGVTQN 896
SARS_CoV_Tor2/1-1255                                    FNGLTVLPPLLTDDMIAAYTAALVSGTATAGWTFGAGAALQIPFAMQMAYRFNGIGVTQN 896
Bat_SL_CoV__Rf4092/1-1234                               FNGLTVLPPLLTDEMIAAYTAALVSGTATAGWTFGAGAALQIPFAMQMAYRFNGIGVTQN 875
BtRf_CoV_JL2012/1-1236                                  FNGLTVLPPLLTDDMIAAYTAALISGTATAGWTFGAGAALQIPFAMQMAYRFNGIGVTQN 877
BtRs_YN2013/1-1233                                      FNGLTVLPPLLTDEMIAAYTAALVSGTATAGWTFGAGAALQIPFAMQMAYRFNGIGVTQN 874
Bat_CoV_Rp/Shaanxi2011/1-1240                           FNGLTVLPPLLTDEMIAAYTAALVSGTATAGWTFGAGAALQIPFAMQMAYRFNGIGVTQN 881
Bat_SARS_CoV_Rf1/2004/1-1241                            FNGLTVLPPLLTDDMIAAYTAALISGTATAGWTFGAGAALQIPFAMQMAYRFNGIGVTQN 882
Bat_CoV/Yunnan2011/1-1241                               FNGLTVLPPLLTDEMIAAYTAALVSGTATAGWTFGAGAALQIPFAMQMAYRFNGIGVTQN 882
SARS_CoV_Rs_672/2006/1-1241                             FNGLTVLPPLLTDEMIAAYTAALVSGTATAGWTFGAGAALQIPFAMQMAYRFNGIGVTQN 882
Bat_SL_CoV_Rs4081/1-1241                                FNGLTVLPPLLTDDMIAAYTAALVSGTATAGWTFGAGAALQIPFAMQMAYRFNGIGVTQN 882
Bat_SL_CoV_Rs4255/1-1241                                FNGLTVLPPLLTDDMIAAYTAALVSGTATAGWTFGAGAALQIPFAMQMAYRFNGIGVTQN 882
Bat_SARS_CoV_Rm1/2004/1-1241                            FNGLTVLPPLLTDEMIAAYTAALVSGTATAGWTFGAGSALQIPFAMQMAYRFNGIGVTQN 882
Bat_SARS_CoV_HKU3-1/1-1242                              FNGLTVLPPLLTDEMVAAYTAALVSGTATAGWTFGAGAALQIPFAMQMAYRFNGIGVTQN 883
Bat_SARS_CoV_Rp3/1-1241                                 FNGLTVLPPLLTDEMIAAYTAALVSGTATAGWTFGAGSALQIPFAMQMAYRFNGIGVTQN 882
Bat_SL_CoV_As6526/1-1241                                FNGLTVLPPLLTDDMIAAYTAALVSGTATAGWTFGAGAALQIPFAMQMAYRFNGIGVTQN 882
Bat_SL_CoV_Rs4237/1-1241                                FNGLTVLPPLLTDDMIAAYTAALVSGTATAGWTFGAGAALQIPFAMQMAYRFNGIGVTQN 882
Bat_SL_CoV_Rs4247/1-1242                                FNGLTVLPPLLTDDMIAAYTAALVSGTATAGWTFGAGAALQIPFAMQMAYRFNGIGVTQN 883
                                                          *  *:* :      . *      .                 :   :  *:* :.:  :

HCoV_NL63/1-1356                                        VLQENQKILAASFNKAINNIVASFSSVNDAITQTAEAIHTVTIALNKIQDVVNQQGSALN 1026
Felis_Catus_CoV_NDL/UU88/1-1467                         VLQENQKILANAFNNAIGNITLALGKVYDAITTIPDGFNSVASALTKIQSVVNQQGEALS 1137
Ferret_CoV/1-1438                                       VLQQNQKILAASFNQAIGNITLAFGKVNSAIQQTAQGLSTVAQALTKVQDVVNSQGKALN 1102
Canine_CoV/1-1457                                       VLNKNQQILANAFNQAIGNITQAFGKVNDAIHQTSQGLATVAKALAKVQDVVNTQGQALS 1123
Mink_CoV_WD1133/1-1429                                  VLQRNQEILAASFNQAIGNITIALGKVNNAIYQTSQSLSTVAQALTKVQDVVNSQGKALN 1092
Mink_CoV_1/1-1439                                       VLQRNQEILAASFNQAIGNITIALGKVNNAIYQTSQSLSTVAQALTKVQDVVNSQGKALN 1102
Mink_CoV_WD1127/1-1438                                  VLQRNQEILAASFNQAIGNITIALGKVNNAIYQTSQSLSTVAQALTKVQDVVNSQGKALN 1101
Rhinolophus_affinis_bat_CoV_HKU-2_related/1-1119        ALVDDVSKIANGFNQLTASV--------------GKLAATTSSALQAIQAVVNQNAAQVE 803
Rhinolophus_bat_CoV_HKU2/1-1128                         ALVDDVNKLANGFNQLTASV--------------GKLALTTSSALQAIQAVVNQNAAQVE 812
```

```
HCoV_OC43/1-1361                  VLSQNQKLIANAFNNALYAI--------------QEGFDATNSALVKIQAVVNANAEALN 1052
HCoV_HKU1/1-1356                  VLNKNQKLIATAFNNALLSI--------------QNGFSATNSALAKIQSVVNSNAQALN 1045
MERS_CoV/1-1353                   VLSENQKLIANKFNQALGAM--------------QTGFTTTNEAFQKVQDAVNNNAQALS 1034
Bat_SL_CoV_ZC45/1-1246            VLYENQKLIANQFNSAIGKI--------------QESLTSTASALGKLQDVVNQNAQALN 933
Bat_SL_CoV_ZXC21/1-1245           VLYENQKLIANQFNSAIGKI--------------QESLTSTASALGKLQDVVNQNAQALN 932
Pangolin_CoV_MP789/1-1265         VLYENQKLIANQFNSAIGKI--------------QDSLSSTASALGKLQDVVNQNAQALN 952
Bat_CoV_RaTG13/1-1269             VLYENQKLIANQFNSAIGKI--------------QDSLSSTASALGKLQDVVNQNAQALN 956
SARS-CoV2/1-1273                  VLYENQKLIANQFNSAIGKI--------------QDSLSSTASALGKLQDVVNQNAQALN 960
Bat_SL_CoV_Rs4084/1-1256          VLYENQKQIANQFNKAISQI--------------QESLTTTSTALGKLQDVVNQNAQALN 943
Bat_SL_CoV_RsSHC014/1-1256        VLYENQKQIANQFNKAISQI--------------QESLTTTSTALGKLQDVVNQNAQALN 943
Bat_SL_CoV_Rs7327/1-1256          VLYENQKQIANQFNKAISQI--------------QESLTTTSTALGKLQDVVNQNAQALN 943
Bat_SL_CoV_Rs9401/1-1256          VLYENQKQIANQFNKAISQI--------------QESLTTTSTALGKLQDVVNQNAQALN 943
Bat_SL_CoV_WIV1/1-1256            VLYENQKQIANQFNKAISQI--------------QESLTTTSTALGKLQDVVNQNAQALN 943
Bat_SL_CoV_Rs3367/1-1256          VLYENQKQIANQFNKAISQI--------------QESLTTTSTALGKLQDVVNQNAQALN 943
Bat_SL_CoV_Rs4231/1-1255          VLYENQKQIANQFNKAISQI--------------QESLTTTSTALGKLQDVVNQNAQALN 942
Bat_SL_CoV_Rs4874/1-1255          VLYENQKQIANQFNKAISQI--------------QESLTTTSTALGKLQDVVNQNAQALN 942
SL_CoV_WIV16/1-1255               VLYENQKQIANQFNKAISQI--------------QESLTTTSTALGKLQDVVNQNAQALN 942
SARS_CoV_civet010/1-1255          VLYENQKQIANQFNKAISQI--------------QESLTTTSTALGKLQDVVNQNAQALN 942
SARS_CoV_Tor2/1-1255              VLYENQKQIANQFNKAISQI--------------QESLTTTSTALGKLQDVVNQNAQALN 942
Bat_SL_CoV__Rf4092/1-1234         VLYENQKQIANQFNKAISQI--------------QESLTTTSTALGKLQDVVNQNAQALN 921
BtRf_CoV_JL2012/1-1236            VLYENQKQIANQFNKAITQI--------------QESLTTTATALGKLQDVVNQNAQALN 923
BtRs_YN2013/1-1233                VLYENQKQIANQFNKAISQI--------------QESLTTTSTALGKLQDVVNQNAQALN 920
Bat_CoV_Rp/Shaanxi2011/1-1240     VLYENQKQIANQFNKAISQI--------------QESLTTTSTALGKLQDVVNQNAQALN 927
Bat_SARS_CoV_Rf1/2004/1-1241      VLYENQKQIANQFNKAITQI--------------QESLTTTSTALGKLQDVVNQNAQALN 928
Bat_CoV/Yunnan2011/1-1241         VLYENQKQIANQFNKAISQI--------------QESLTTTSTALGKLQDVVNQNAQALN 928
SARS_CoV_Rs_672/2006/1-1241       VLYENQKQIANQFNKAISQI--------------QESLTTTSTALGKLQDVVNQNAQALN 928
Bat_SL_CoV_Rs4081/1-1241          VLYENQKQIANQFNKAISQI--------------QESLTTTSTALGKLQDVVNQNAQALN 928
Bat_SL_CoV_Rs4255/1-1241          VLYENQKQIANQFNKAISQI--------------QESLTTTSTALGKLQDVVNQNAQALN 928
Bat_SARS_CoV_Rm1/2004/1-1241      VLYENQKQIANQFNKAISQI--------------QESLTTTSTALGKLQDVVNQNAQALN 928
Bat_SARS_CoV_HKU3-1/1-1242        VLYENQKLIANQFNSAIGKI--------------QESLSSTASALGKLQDVVNQNAQALN 929
Bat_SARS_CoV_Rp3/1-1241           VLYENQKQIANQFNKAISQI--------------QESLTTTSTALGKLQDVVNQNAQALN 928
Bat_SL_CoV_As6526/1-1241          VLYENQKQIANQFNKAISQI--------------QESLTTTSTALGKLQDVVNQNAQALN 928
Bat_SL_CoV_Rs4237/1-1241          VLYENQKQIANQFNKAISQI--------------QESLTTTSTALGKLQDVVNQNAQALN 928
Bat_SL_CoV_Rs4247/1-1242          VLYENQKQIANQFNKAISQI--------------QESLTTTSTALGKLQDVVNQNAQALN 929
                                  .*  : . :*  **.    :                   :.  *:  :* .** :.  :.


HCoV_NL63/1-1356                  HLTSQLRHNFQAISNSIQAIYDRLDSIQADQQVDRLITGRLAALNAFVSQVLNKYTEVRG 1086
Felis_Catus_CoV_NDL/UU88/1-1467   QLTSQLQKNFQAISSSIAEIYNRLEKVEADAQVDRLITGRLAALNAYVSQTLTQYAEVKA 1197
Ferret_CoV/1-1438                 HLTAQLQNNFQAISSSIEDIYYKLDEVNADAQVDRLITGRLASLNAFVTQTLTNQAQIRA 1162
Canine_CoV/1-1457                 HLTVQLQNNFQAISSSISDIYNRLDELSADAQVDRLITGRLTALNAFVSQTLTRQAEVRA 1183
```

```
Mink_CoV_WD1133/1-1429                                HLTLQLQNNFQAISSSIQDIYYKLDDINADAQVDRLITGRLAALNAFVTQTLTRQAEVRA 1152
Mink_CoV_1/1-1439                                     HLTLQLQNNFQAISSSIQDIYYKLDDINADAQVDRLITGRLAALNAFVTQTLTRQAEVRA 1162
Mink_CoV_WD1127/1-1438                                HLTLQLQNNFQAISSSIQDIYYKLDDINADAQVDRLITGRLAALNAFVTQTLTRQAEVRA 1161
Rhinolophus_affinis_bat_CoV_HKU-2_related/1-1119      ALVSGISENFGAISTNFRVISQRLDKLEADVQMDRLINGRMNVLQLFVTNYKLRVAELRN 863
Rhinolophus_bat_CoV_HKU2/1-1128                       SLVSGITENFGAISTNFKVISQRLDKLEADVQMDRLINGRMNVLQLFVTNYKLKIAELRN 872
HCoV_OC43/1-1361                                      NLLQQLSNRFGAISASLQEILSRLDALEAEAQIDRLINGRLTALNAYVSQQLSDSTLVKF 1112
HCoV_HKU1/1-1356                                      SLLQQLFNKFGAISSSLQEILSRLDALEAQVQIDRLINGRLTALNAYVSQQLSDISLVKF 1105
MERS_CoV/1-1353                                       KLASELSNTFGAISASIGDIIQRLDVLEQDAQIDRLINGRLTTLNAFVAQQLVRSESAAL 1094
Bat_SL_CoV_ZC45/1-1246                                TLVKQLSSNFGAISSVLNDILSRLDKVEAEVQIDRLITGRLQSLQTYVTQQLIRAAEIRA 993
Bat_SL_CoV_ZXC21/1-1245                               TLVKQLSSNFGAISSVLNDILSRLDKVEAEVQIDRLITGRLQSLQTYVTQQLIRAAEIRA 992
Pangolin_CoV_MP789/1-1265                             TLVKQLSSNFGAISSVLNDILSRLDKVEAEVQIDRLITGRLQSLQTYVTQQLIRAAEIRA 1012
Bat_CoV_RaTG13/1-1269                                 TLVKQLSSNFGAISSVLNDILSRLDKVEAEVQIDRLITGRLQSLQTYVTQQLIRAAEIRA 1016
SARS-CoV2/1-1273                                      TLVKQLSSNFGAISSVLNDILSRLDKVEAEVQIDRLITGRLQSLQTYVTQQLIRAAEIRA 1020
Bat_SL_CoV_Rs4084/1-1256                              TLVKQLSSNFGAISSVLNDILSRLDKVEAEVQIDRLITGRLQSLQTYVTQQLIRAAEIRA 1003
Bat_SL_CoV_RsSHC014/1-1256                            TLVKQLSSNFGAISSVLNDILSRLDKVEAEVQIDRLITGRLQSLQTYVTQQLIRAAEIRA 1003
Bat_SL_CoV_Rs7327/1-1256                              TLVKQLSSNFGAISSVLNDILSRLDKVEAEVQIDRLITGRLQSLQTYVTQQLIRAAEIRA 1003
Bat_SL_CoV_Rs9401/1-1256                              TLVKQLSSNFGAISSVLNDILSRLDKVEAEVQIDRLITGRLQSLQTYVTQQLIRAAEIRA 1003
Bat_SL_CoV_WIV1/1-1256                                TLVKQLSSNFGAISSVLNDILSRLDKVEAEVQIDRLITGRLQSLQTYVTQQLIRAAEIRA 1003
Bat_SL_CoV_Rs3367/1-1256                              TLVKQLSSNFGAISSVLNDILSRLDKVEAEVQIDRLITGRLQSLQTYVTQQLIRAAEIRA 1003
Bat_SL_CoV_Rs4231/1-1255                              TLVKQLSSNFGAISSVLNDILSRLDKVEAEVQIDRLITGRLQSLQTYVTQQLIRAAEIRA 1002
Bat_SL_CoV_Rs4874/1-1255                              TLVKQLSSNFGAISSVLNDILSRLDKVEAEVQIDRLITGRLQSLQTYVTQQLIRAAEIRA 1002
SL_CoV_WIV16/1-1255                                   TLVKQLSSNFGAISSVLNDILSRLDKVEAEVQIDRLITGRLQSLQTYVTQQLIRAAEIRA 1002
SARS_CoV_civet010/1-1255                              TLVKQLSSNFGAISSVLNDILSRLDKVEAEVQIDRLITGRLQSLQTYVTQQLIRAAEIRA 1002
SARS_CoV_Tor2/1-1255                                  TLVKQLSSNFGAISSVLNDILSRLDKVEAEVQIDRLITGRLQSLQTYVTQQLIRAAEIRA 1002
Bat_SL_CoV__Rf4092/1-1234                             TLVKQLSSNFGAISSVLNDILSRLDKVEAEVQIDRLITGRLQSLQTYVTQQLIRAAEIRA 981
BtRf_CoV_JL2012/1-1236                                TLVKQLSSNFGAISSALNDILSRLDKVEAEVQIDRLITGRLQSLQTYVTQQLIRAAEIRA 983
BtRs_YN2013/1-1233                                    TLVKQLSSNFGAISSVLNDILSRLDKVEAEVQIDRLITGRLQSLQTYVTQQLIRAAEIRA 980
Bat_CoV_Rp/Shaanxi2011/1-1240                         TLVKQLSSNFGAISSVLNDILSRLDKVEAEVQIDRLITGRLQSLQTYVTQQLIRAAEIRA 987
Bat_SARS_CoV_Rf1/2004/1-1241                          TLVKQLSSNFGAISSALNDILSRLDKVEAEVQIDRLITGRLQSLQTYVTQQLIRAAEIRA 988
Bat_CoV/Yunnan2011/1-1241                             TLVKQLSSNFGAISSVLNDILSRLDKVEAEVQIDRLITGRLQSLQTYVTQQLIRAAEIRA 988
SARS_CoV_Rs_672/2006/1-1241                           TLVKQLSSNFGAISSVLNDILSRLDKVEAEVQIDRLITGRLQSLQTYVTQQLIRAAEIRA 988
Bat_SL_CoV_Rs4081/1-1241                              TLVKQLSSNFGAISSVLNDILSRLDKVEAEVQIDRLITGRLQSLQTYVTQQLIRAAEIRA 988
Bat_SL_CoV_Rs4255/1-1241                              TLVKQLSSNFGAISSVLNDILSRLDKVEAEVQIDRLITGRLQSLQTYVTQQLIRAAEIRA 988
Bat_SARS_CoV_Rm1/2004/1-1241                          TLVKQLSSNFGAISSVLNDILSRLDKVEAEVQIDRLITGRLQSLQTYVTQQLIRAAEIRA 988
Bat_SARS_CoV_HKU3-1/1-1242                            TLVKQLSSNFGAISSVLNDILSRLDKVEAEVQIDRLITGRLQSLQTYVTQQLIRAAEIRA 989
Bat_SARS_CoV_Rp3/1-1241                               TLVKQLSSNFGAISSVLNDILSRLDKVEAEVQIDRLITGRLQSLQTYVTQQLIRAAEIRA 988
Bat_SL_CoV_As6526/1-1241                              TLVKQLSSNFGAISSVLNDILSRLDKVEAEVQIDRLITGRLQSLQTYVTQQLIRAAEIRA 988
Bat_SL_CoV_Rs4237/1-1241                              TLVKQLSSNFGAISSVLNDILSRLDKVEAEVQIDRLITGRLQSLQTYVTQQLIRAAEIRA 988
Bat_SL_CoV_Rs4247/1-1242                              TLVKQLSSNFGAISSVLNDILSRLDKVEAEVQIDRLITGRLQSLQTYVTQQLIRAAEIRA 989
                                                       *   :   * ***  :  *  :*: :. : *:****.**:  *: :*::
```

| | | |
|---|---|---|
| HCoV_NL63/1-1356 | SRRLAQQKINECVKSQSNRYGFCGNGTHIFSIVNSAPDGLLFLHTVLLPTDYKNVKAWSG | 1146 |
| Felis_Catus_CoV_NDL/UU88/1-1467 | SRQLAMEKVNECVKSQSDRYGFCGNGTHLFSLANSAPDGLLFFHTVLLPTEWEEVTAWSG | 1257 |
| Ferret_CoV/1-1438 | SRQLSKEKINECVRSQSSRFGFCGNGTHLFSLANAAPRGVMLFHTVLLPTSYKTVTAWSG | 1222 |
| Canine_CoV/1-1457 | SRQLAKDKVNECVRSQSQRFGFCGNGTHLFSLANAAPNGMIFFHSVLLPTAYETVTAWSG | 1243 |
| Mink_CoV_WD1133/1-1429 | SRQLAKEKVNECVRSQSSRFGFCGNGTHLFSLTNAAPNGMVFFHTVLVPTAYETVTAWSG | 1212 |
| Mink_CoV_1/1-1439 | SRQLAKEKVNECVRSQSSRFGFCGNGTHLFSLANAAPNGMVFFHTVLVPTAYQTVTAWSG | 1222 |
| Mink_CoV_WD1127/1-1438 | SRQLAKQKVNECVRSQSSRFGFCGNGTHLFSLANAAPNGMVFFHTVLVPTAYQTVTAWSG | 1221 |
| Rhinolophus_affinis_bat_CoV_HKU-2_related/1-1119 | THRYVQSLINECVYAQSLRNGFCGQGLHVLSLMQNAPSGIMFFHYSLVPNDTIIVRTTPG | 923 |
| Rhinolophus_bat_CoV_HKU2/1-1128 | THRYVQSLINECVYAQSLRNGFCGQGLHVLSLMQNAPSGIMFFHYSLIPNNTITVKTTPG | 932 |
| HCoV_OC43/1-1361 | SAAQAMEKVNECVKSQSSRINFCGNGNHIISLVQNAPYGLYFIHFSYVPTKYVTARVSPG | 1172 |
| HCoV_HKU1/1-1356 | GAALAMEKVNECVKSQSPRINFCGNGNHILSLVQNAPYGLLFMHFSYKPISFKTVLVSPG | 1165 |
| MERS_CoV/1-1353 | SAQLAKDKVNECVKAQSKRSGFCGQGTHIVSFVVNAPNGLYFMHVGYYPSNHIEVVSAYG | 1154 |
| Bat_SL_CoV_ZC45/1-1246 | SANLAATKMSECVLGQSKRVDFCGKGYHLMSFPQSAPHGVVFLHVTYIPSQEKNFTTAPA | 1053 |
| Bat_SL_CoV_ZXC21/1-1245 | SANLAATKMSECVLGQSKRVDFCGKGYHLMSFPQSAPHGVVFLHVTYIPSQEKNFTTAPA | 1052 |
| Pangolin_CoV_MP789/1-1265 | SANLAATKMSECVLGQSKRVDFCGKGYHLMSFPQSAPHGVVFLHVTYVPSQEKNFTTTPA | 1072 |
| Bat_CoV_RaTG13/1-1269 | SANLAATKMSECVLGQSKRVDFCGKGYHLMSFPQSAPHGVVFLHVTYVPAQEKNFTTAPA | 1076 |
| SARS-CoV2/1-1273 | SANLAATKMSECVLGQSKRVDFCGKGYHLMSFPQSAPHGVVFLHVTYVPAQEKNFTTAPA | 1080 |
| Bat_SL_CoV_Rs4084/1-1256 | SANLAATKMSECVLGQSKRVDFCGKGYHLMSFPQAAPHGVVFLHVTYVPSQERNFTTAPA | 1063 |
| Bat_SL_CoV_RsSHC014/1-1256 | SANLAATKMSECVLGQSKRVDFCGKGYHLMSFPQAAPHGVVFLHVTYVPSQERNFTTAPA | 1063 |
| Bat_SL_CoV_Rs7327/1-1256 | SANLAATKMSECVLGQSKRVDFCGKGYHLMSFPQAAPHGVVFLHVTYVPSQERNFTTAPA | 1063 |
| Bat_SL_CoV_Rs9401/1-1256 | SANLAATKMSECVLGQSKRVDFCGKGYHLMSFPQAAPHGVVFLHVTYVPSQERNFTTAPA | 1063 |
| Bat_SL_CoV_WIV1/1-1256 | SANLAATKMSECVLGQSKRVDFCGKGYHLMSFPQAAPHGVVFLHVTYVPSQERNFTTAPA | 1063 |
| Bat_SL_CoV_Rs3367/1-1256 | SANLAATKMSECVLGQSKRVDFCGKGYHLMSFPQAAPHGVVFLHVTYVPSQERNFTTAPA | 1063 |
| Bat_SL_CoV_Rs4231/1-1255 | SANLAATKMSECVLGQSKRVDFCGKGYHLMSFPQAAPHGVVFLHVTCVPSQERNFTTAPA | 1062 |
| Bat_SL_CoV_Rs4874/1-1255 | SANLAATKMSECVLGQSKRVDFCGKGYHLMSFPQAAPHGVVFLHVTYVPSQERNFTTAPA | 1062 |
| SL_CoV_WIV16/1-1255 | SANLAATKMSECVLGQSKRVDFCGKGYHLMSFPQAAPHGVVFLHVTYVPSQERNFTTAPA | 1062 |
| SARS_CoV_civet010/1-1255 | SANLAATKMSECVLGQSKRVDFCGKGYHLMSFPQAAPHGVVFLHVTYVPSQERNFTTAPA | 1062 |
| SARS_CoV_Tor2/1-1255 | SANLAATKMSECVLGQSKRVDFCGKGYHLMSFPQAAPHGVVFLHVTYVPSQERNFTTAPA | 1062 |
| Bat_SL_CoV__Rf4092/1-1234 | SANLAATKMSECVLGQSKRVDFCGRGYHLMSFPQAAPHGVVFLHVTYVPSHEKNFTTAPA | 1041 |
| BtRf_CoV_JL2012/1-1236 | SANLAATKMSECVLGQSKRVDFCGKGYHLMSFPQSAPHGVVFLHVTYVPAQEKNFTTAPA | 1043 |
| BtRs_YN2013/1-1233 | SANLAATKMSECVLGQSKRVDFCGRGYHLMSFPQAAPHGVVFLHVTYVPSHEKNFTTAPA | 1040 |
| Bat_CoV_Rp/Shaanxi2011/1-1240 | SANLAATKMSECVLGQSKRVDFCGKGYHLMSFPQAAPHGVVFLHVTYVPSQERNFTTAPA | 1047 |
| Bat_SARS_CoV_Rf1/2004/1-1241 | SANLAATKMSECVLGQSKRVDFCGKGYHLMSFPQSAPHGVVFLHVTYVPSQEKNFTTAPA | 1048 |
| Bat_CoV/Yunnan2011/1-1241 | SANLAATKMSECVLGQSKRVDFCGKGYHLMSFPQAAPHGVVFLHVTYVPSQERNFTTAPA | 1048 |
| SARS_CoV_Rs_672/2006/1-1241 | SANLAATKMSECVPGQSKRVDFCGRGYHLMSFPQAAPHGVVFLHVTYVPSQEKNFTTAPA | 1048 |
| Bat_SL_CoV_Rs4081/1-1241 | SANLAATKMSECVLGQSKRVDFCGRGYHLMSFPQAAPHGVVFLHVTYVPSQEKNFTTAPA | 1048 |
| Bat_SL_CoV_Rs4255/1-1241 | SANLAATKMSECVLGQSKRVDFCGRGYHLMSFPQAAPHGVVFLHVTYVPSQEKNFTTAPA | 1048 |
| Bat_SARS_CoV_Rm1/2004/1-1241 | SANLAATKMSECVLGQSKRVDFCGKGYHLMSFPQAAPHGVVFLHVTYVPSQERNFTTAPA | 1048 |
| Bat_SARS_CoV_HKU3-1/1-1242 | SANLAATKMSECVLGQSKRVDFCGKGYHLMSFPQSAPHGVVFLHVTYVPSQEKNFTTAPA | 1049 |

```
Bat_SARS_CoV_Rp3/1-1241                               SANLAATKMSECVLGQSKRVDFCGKGYHLMSFPQAAPHGVVFLHVTYVPSQERNFTTAPA 1048
Bat_SL_CoV_As6526/1-1241                              SANLAATKMSECVLGQSKRVDFCGRGYHLMSFPQAAPHGVVFLHVTYVPSQEKNFTTAPA 1048
Bat_SL_CoV_Rs4237/1-1241                              SANLAATKMSECVLGQSKRVDFCGRGYHLMSFPQAAPHGVVFLHVTYVPSQEKNFTTAPA 1048
Bat_SL_CoV_Rs4247/1-1242                              SANLAATKMSECVLGQSKRVDFCGRGYHLMSFPQAAPHGVVFLHVTYVPSQEKNFTTAPA 1049
                                                              :.*** .** * .***.* *:.*:   ** *: ::*    *          .
```

```
HCoV_NL63/1-1356                                      ICVDG---IYGYVLRQPNLVLYSDN------GVFRVTSRVMFQPRLPVLSDFVQIYNCNV 1197
Felis_Catus_CoV_NDL/UU88/1-1467                       ICVND---TYAYVLKDFDQSIFSYN------GTYMVTPRNMFQPRKPQMSDFVQITSCEV 1308
Ferret_CoV/1-1438                                     VCAISNNKTMGFIVKDVSLTLFKNHD-----DKFYLTPRTMYEPRVATMSDFVQIESCTT 1277
Canine_CoV/1-1457                                     ICASDGDRTFGLVVKDVQLTLFRNLD-----GKFYLTPRTMYQPRAATSSDFVQIEGCDV 1298
Mink_CoV_WD1133/1-1429                                ICASDDSRTFGLIVKDVSLTLFRNHD-----GKFYLTPRTMYQPRVATSADFVQIVDCDV 1267
Mink_CoV_1/1-1439                                     ICASDGDRTFGLVVKDVSLTLFRNHN-----NGFYLTPRTMYQPRVATSADFVQIVACDV 1277
Mink_CoV_WD1127/1-1438                                ICASDGDRTFGLIVKDVSLTLFRNYN-----NSFYLTPRTMYQPRIATSADFVQIADCDV 1276
Rhinolophus_affinis_bat_CoV_HKU-2_related/1-1119      LCEDNSLGSKCIAPK---DGIFVSAN----LSYWQWSPRNIYRPENISVLNVAV-VSRGL 975
Rhinolophus_bat_CoV_HKU2/1-1128                       LCESDELGSKCIVAK---DGVLVSAN----LSYWQWSPRNLYKPENLTFANVIA-VSRGA 984
HCoV_OC43/1-1361                                      LCIAGDR---GIAPK---SGYFVNVN-----NTWMYTGSGYYYPEPITENNVVVMSTCAV 1221
HCoV_HKU1/1-1356                                      LCISGDV---GIAPK---QGYFIKHN-----DHWMFTGSSYYYPEPISDKNVVFMNTCSV 1214
MERS_CoV/1-1353                                       LCDAANP-TNCIAPV---NGYFIKTNNTRIVDEWSYTGSSFYAPEPITSLNTKY-VAPQV 1209
Bat_SL_CoV_ZC45/1-1246                                ICHEGK----AHFPR---EGVFVSNG-----THWFVTQRNFYEPKIITTDNTFVSGNCDV 1101
Bat_SL_CoV_ZXC21/1-1245                               ICHEGK----AHFPR---EGVFVSNG-----THWFVTQRNFYEPQIITTDNTFVSGNCDV 1100
Pangolin_CoV_MP789/1-1265                             ICHEGK----AHFPR---EGVFVSNG-----THWFVTQRNFYEPQIITTDNTFVSGSCDV 1120
Bat_CoV_RaTG13/1-1269                                 ICHDGK----AHFPR---EGVFVSNG-----THWFVTQRNFYEPQIITTDNTFVSGSCDV 1124
SARS-CoV2/1-1273                                      ICHDGK----AHFPR---EGVFVSNG-----THWFVTQRNFYEPQIITTDNTFVSGNCDV 1128
Bat_SL_CoV_Rs4084/1-1256                              ICHEGK----AYFPR---EGVFVFNG-----TSWFITQRNFFSPQIITTDNTFVSGSCDV 1111
Bat_SL_CoV_RsSHC014/1-1256                            ICHEGK----AYFPR---EGVFVFNG-----TSWFITQRNFFSPQIITTDNTFVSGSCDV 1111
Bat_SL_CoV_Rs7327/1-1256                              ICHEGK----AYFPR---EGVFVFNG-----TSWFITQRNFFSPQIITTDNTFVSGNCDV 1111
Bat_SL_CoV_Rs9401/1-1256                              ICHEGK----AYFPR---EGVFVFNG-----TSWFITQRNFFSPQIITTDNTFVSGNCDV 1111
Bat_SL_CoV_WIV1/1-1256                                ICHEGK----AYFPR---EGVFVFNG-----TSWFITQRNFFSPQIITTDNTFVSGSCDV 1111
Bat_SL_CoV_Rs3367/1-1256                              ICHEGK----AYFPR---EGVFVFNG-----TSWFITQRNFFSPQIITTDNTFVSGSCDV 1111
Bat_SL_CoV_Rs4231/1-1255                              ICHEGK----AYFPR---EGVFVFNG-----TSWFITQRNFFSPQIITTDNTFVSGSCDV 1110
Bat_SL_CoV_Rs4874/1-1255                              ICHEGK----AYFPR---EGVFVFNG-----TSWFITQRNFFSPQIITTDNTFVSGSCDV 1110
SL_CoV_WIV16/1-1255                                   ICHEGK----AYFPR---EGVFVFNG-----TSWFITQRNFFSPQIITTDNTFVSGSCDV 1110
SARS_CoV_civet010/1-1255                              ICHEGK----AYFPR---EGVFVFNG-----TSWFITQRNFFSPQIITTDNTFVSGNCDV 1110
SARS_CoV_Tor2/1-1255                                  ICHEGK----AYFPR---EGVFVFNG-----TSWFITQRNFFSPQIITTDNTFVSGNCDV 1110
Bat_SL_CoV__Rf4092/1-1234                             ICHEGK----AYFPR---EGVFVSNG-----TSWFITQRNFYSPQIITTDNTFVAGNCDV 1089
BtRf_CoV_JL2012/1-1236                                ICHEGK----AYFPR---EGVFVSNG-----SSWFITQRNFYSPQIITTDNTFVAGSCDV 1091
BtRs_YN2013/1-1233                                    ICHEGK----AYFPR---EGVFVSNG-----TSWFITQRNFYSPQIITTDNTFVAGNCDV 1088
Bat_CoV_Rp/Shaanxi2011/1-1240                         ICHEGK----AYFPR---EGVFVSNG-----TSWFITQRNFYSPQIITTDNTFVAGSCNV 1095
Bat_SARS_CoV_Rf1/2004/1-1241                          ICHEGK----AYFPR---EGVFVSNG-----SSWFITQRNFYSPQIITTDNTFVAGSCDV 1096
Bat_CoV/Yunnan2011/1-1241                             ICHQGK----AYFPR---EGVFVSNG-----TSWFITQRNFFSPQIITTDNTFVSGNCDV 1096
```

```
SARS_CoV_Rs_672/2006/1-1241                       ICHEGK----AYFPR---EGVFVSNG-----TSWFITQRNFYSPQIITTDNTFVAGNCDV 1096
Bat_SL_CoV_Rs4081/1-1241                          ICHEGK----AYFPR---EGVFVSNG-----TSWFITQRNFYSPQIITTDNTFVAGNCDV 1096
Bat_SL_CoV_Rs4255/1-1241                          ICHEGK----AYFPR---EGVFVSNG-----TSWFITQRNFYSPQIITTDNTFVAGNCDV 1096
Bat_SARS_CoV_Rm1/2004/1-1241                      ICHEGK----AYFPR---EGVFVSNG-----TSWFITQRNFYSPQIITTDNTFVAGNCDV 1096
Bat_SARS_CoV_HKU3-1/1-1242                        ICHEGK----AYFPR---EGVFVSNG-----TSWFITQRNFYSPQLITTDNTFVSGNCDV 1097
Bat_SARS_CoV_Rp3/1-1241                           ICHEGK----AYFPR---EGVFVSNG-----TSWFITQRNFYSPQIITTDNTFVAGSCDV 1096
Bat_SL_CoV_As6526/1-1241                          ICHEGK----AYFPR---EGVFVSNG-----TSWFITQRNFYSPQIITTDNTFVAGNCDV 1096
Bat_SL_CoV_Rs4237/1-1241                          ICHEGK----AYFPR---EGVFVSNG-----TSWFITQRNFYSPQIITTDNTFVAGNCDV 1096
Bat_SL_CoV_Rs4247/1-1242                          ICHEGK----AYFPR---EGVFVSNG-----TSWFITQRNFYSPQIITTDNTFVAGNCDV 1097
                                                  :*                               :  :    : *.     :
```

```
HCoV_NL63/1-1356                                  TFVNISRVELHTVIPD--YVDVNKTLQEFA-QNLPKYVKPNFDLT----PFNLTYLNLSS 1250
Felis_Catus_CoV_NDL/UU88/1-1467                   TFLNATYTKFQDIVID--YIDINKTISDMLDKYNYTTSELDLQLE----IFNQTKLNLTA 1362
Ferret_CoV/1-1438                                 TFVNATVAELPSIIPD--YIDINGTIKDMLDQYKPNWTVPELTID----VFNQTYLNLTG 1331
Canine_CoV/1-1457                                 LFVNATVIDLPSIIPD--YIDINQTVQDILENYRPNWTVPELTLD----IFNATYLNLTG 1352
Mink_CoV_WD1133/1-1429                            LFVNATILELPSIVPD--YIDINKTVQDLLDSYKPNWTVPEFSLD----IFNQTYLNITN 1321
Mink_CoV_1/1-1439                                 LFVNATILELPSIIPD--YIDINKTVQDLLDSYKPNWTVPDFSLD----IFNQTYLNITN 1331
Mink_CoV_WD1127/1-1438                            LFVNATILELPSIIPD--YIDINKTVQDLLDSYKPNWTVPELSLD----IFNQTYLNITN 1330
Rhinolophus_affinis_bat_CoV_HKU-2_related/1-1119  NYTTLNHTFDIPQLND--TFP----IEEEFREYFQNMSSELQALK--DLTADMAKLNLTA 1027
Rhinolophus_bat_CoV_HKU2/1-1128                   NYTTLNRTFDIPELNS--TFP----IDEEFREYFQNMSSELQALK--NLTADMSKLNISA 1036
HCoV_OC43/1-1361                                  NYTKAPYVMLNTSIPN--LPD----FKEELDQWFKNQTSVAPDLS--LDYINVTFLDLQV 1273
HCoV_HKU1/1-1356                                  NFTKAPLVYLNHSVPK--LSD----FESELSHWFKNQTSIAPNLTLNLHTINATFLDLYY 1268
MERS_CoV/1-1353                                   TYQNISTNLPPPLLGNSTGID----FQDELDEFFKNVSTSIPNFG-SLTQINTTLLDLTY 1264
Bat_SL_CoV_ZC45/1-1246                            VIGIINNTVYDPLQPE--LDS----FKEELDKYFKNHTSPDIDLG-DISGINASVVNIQK 1154
Bat_SL_CoV_ZXC21/1-1245                           VIGIINNTVYDPLQPE--LDS----FKEELDKYFKNHTSPDIDLG-DISGINASVVNIQK 1153
Pangolin_CoV_MP789/1-1265                         VIGIVNNTVYDPLQPE--LDS----FKEELDKYFKNHTSPDVDLG-DISGINASVVNIQK 1173
Bat_CoV_RaTG13/1-1269                             VIGIVNNTVYDPLQPE--LDS----FKEELDKYFKNHTSPDVDLG-DISGINASVVNIQK 1177
SARS-CoV2/1-1273                                  VIGIVNNTVYDPLQPE--LDS----FKEELDKYFKNHTSPDVDLG-DISGINASVVNIQK 1181
Bat_SL_CoV_Rs4084/1-1256                          VIGIINNTVYDPLQPE--LDS----FKEELDKYFKNHTSPDVDLG-DISGINASVVNIQK 1164
Bat_SL_CoV_RsSHC014/1-1256                        VIGIINNTVYDPLQPE--LDS----FKEELDKYFKNHTSPDVDLG-DISGINASVVNIQK 1164
Bat_SL_CoV_Rs7327/1-1256                          VIGIINNTVYDPLQPE--LDS----FKEELDKYFKNHTSPDVDLG-DISGINASVVNIQK 1164
Bat_SL_CoV_Rs9401/1-1256                          VIGIINNTVYDPLQPE--LDS----FKEELDKYFKNHTSPDVDLG-DISGINASVVNIQK 1164
Bat_SL_CoV_WIV1/1-1256                            VIGIINNTVYDPLQPE--LDS----FKEELDKYFKNHTSPDVDLG-DISGINASVVNIQK 1164
Bat_SL_CoV_Rs3367/1-1256                          VIGIINNTVYDPLQPE--LDS----FKEELDKYFKNHTSPDVDLG-DISGINASVVNIQK 1164
Bat_SL_CoV_Rs4231/1-1255                          VIGIINNTVYDPLQPE--LDS----FKEELDKYFKNHTSPDVDLG-DISGINASVVNIQK 1163
Bat_SL_CoV_Rs4874/1-1255                          VIGIINNTVYDPLQPE--LDS----FKEELDKYFKNHTSPDVDLG-DISGINASVVNIQK 1163
SL_CoV_WIV16/1-1255                               VIGIINNTVYDPLQPE--LDS----FKEELDKYFKNHTSPDVDLG-DISGINASVVNIQK 1163
SARS_CoV_civet010/1-1255                          VIGIINNTVYDPLQPE--LDS----FKEELDKYFKNHTSPDVDLG-DISGINASVVNIQE 1163
SARS_CoV_Tor2/1-1255                              VIGIINNTVYDPLQPE--LDS----FKEELDKYFKNHTSPDVDLG-DISGINASVVNIQK 1163
Bat_SL_CoV__Rf4092/1-1234                         VIGIINNTVYDPLQPE--LDS----FKEELDKYFKNHTSPDVDLG-DISGINASVVNIQK 1142
```

```
BtRf_CoV_JL2012/1-1236                            VIGIINNTVYDPLQPE--LDS----FKQELDKYFKNHTSPDVDLG-DISGINASVVDIQK 1144
BtRs_YN2013/1-1233                                VIGIINNTVYDPLQPE--LDS----FKEELDKYFKNHTSPDVDLG-DISGINASVVNIQK 1141
Bat_CoV_Rp/Shaanxi2011/1-1240                     VIGIINNTVYDPLQPE--LDS----FKEELDKYFKNHTSPDVDLG-DISGINASVVNIQK 1148
Bat_SARS_CoV_Rf1/2004/1-1241                      VIGIINNTVYDPLQPE--LDS----FKQELDKYFKNHTSPDVDLG-DISGINASVVDIQK 1149
Bat_CoV/Yunnan2011/1-1241                         VIGIINNTVYDPLQPE--LDS----FKEELDKYFKNHTSPDVDLG-DISGINASVVNIQK 1149
SARS_CoV_Rs_672/2006/1-1241                       VIGIINNTVYDPLQPE--LDS----FKEELDKYFKNHTSPDVDLG-DISGINASVVNIQK 1149
Bat_SL_CoV_Rs4081/1-1241                          VIGIINNTVYDPLQPE--LDS----FKEELDKYFKNHTSPDVDLG-DISGINASVVNIQK 1149
Bat_SL_CoV_Rs4255/1-1241                          VIGIINNTVYDPLQPE--LDS----FKEELDKYFKNHTSPDVDLG-DISGINASVVNIQK 1149
Bat_SARS_CoV_Rm1/2004/1-1241                      VIGIINNTVYDPLQPE--LDS----FKEELDKYFKNHTSPDVDLG-DISGINASVVNIQK 1149
Bat_SARS_CoV_HKU3-1/1-1242                        VIGIINNTVYDPLQPE--LDS----FKEELDKYFKNHTSPDVDLG-DISGINASVVNIQK 1150
Bat_SARS_CoV_Rp3/1-1241                           VIGIINNTVYDPLQPE--LDS----FKEELDKYFKNHTSPDVDLG-DISGINASVVNIQK 1149
Bat_SL_CoV_As6526/1-1241                          VIGIINNTVYDPLQPE--LDS----FKEELDKYFKNHTSPDVDLG-DISGINASVVNIQK 1149
Bat_SL_CoV_Rs4237/1-1241                          VIGIINNTVYDPLQPE--LDS----FKEELDKYFKNHTSPDVDLG-DISGINASVVNIQK 1149
Bat_SL_CoV_Rs4247/1-1242                          VIGIINNTVYDPLQPE--LDS----FKEELDKYFKNHTSPDVDLG-DISGINASVVNIQK 1150
                                                                 .         ...       .       :       : : :::


HCoV_NL63/1-1356                                  ELKQLEAKTASLFQTTVELQGLIDQINSTYVDLKLLNRFENYIKWPWWVWLIISVVFVVL 1310
Felis_Catus_CoV_NDL/UU88/1-1467                   EIDQLEQRADNLTTIAHELQQYIDNLNKTLVDLKWLNRIETYVKWPWYVWLLIGLVIVFC 1422
Ferret_CoV/1-1438                                 EINELENRSVILQQTVLELESLIDNINGTLVNLEWLNRVETFVKWPWWVWVIIGLILLIA 1391
Canine_CoV/1-1457                                 EIDDLEFRSEKLHNTTVELAILIDNINNTLVNLEWLNRIETYVKWPWYVWLLIGLVVVFC 1412
Mink_CoV_WD1133/1-1429                            EINDLENRSVVLYNTTKELELLIQSINNTLVDLEWLNKIETYVKWPWYVWVLIALIFLFV 1381
Mink_CoV_1/1-1439                                 EINDLENRSVVLYNTTKELELLIQSINNTLVDLEWLNKIETYVKWPWYVWVLIALIFLFV 1391
Mink_CoV_WD1127/1-1438                            EINDLENRSVVLYNTTKELELLIQSINNTLVDLEWLNKIETYVKWPWYVWVLIALIFLFV 1390
Rhinolophus_affinis_bat_CoV_HKU-2_related/1-1119  EIKLI--------------NEIAVNVSNMHIEVEKFQKYVNYVKWAWWQWLIIFIALVLL 1073
Rhinolophus_bat_CoV_HKU2/1-1128                   EIQLI--------------NEIAHNVSNMRVEVEKFQRYVNYVKWAWWQWLIIFIALTLL 1082
HCoV_OC43/1-1361                                  EMNRL--------------QEAIKVLNQSYINLKDIGTYEYYVKWPWYVWLLICLAGVAM 1319
HCoV_HKU1/1-1356                                  EMNLI--------------QESIKSLNNSYINLKDIGTYEMYVKWPWYVWLLISFSFIIF 1314
MERS_CoV/1-1353                                   EMLSL--------------QQVVKALNESYIDLKELGNYTYYNKWPWYIWLGFIAGLVAL 1310
Bat_SL_CoV_ZC45/1-1246                            EIDRL--------------NEVARNLNESLIDLQELGKYEQYIKWPWYVWLGFIAGLIAI 1200
Bat_SL_CoV_ZXC21/1-1245                           EIDRL--------------NEVARNLNESLIDLQELGKYEHYIKWPWYVWLGFIAGLIAI 1199
Pangolin_CoV_MP789/1-1265                         EIDRL--------------NEVAKNLNESLIDLQELGKYEQYIKWPWYIWLGFIAGLIAI 1219
Bat_CoV_RaTG13/1-1269                             EIDRL--------------NEVAKNLNESLIDLQELGKYEQYIKWPWYIWLGFIAGLIAI 1223
SARS-CoV2/1-1273                                  EIDRL--------------NEVAKNLNESLIDLQELGKYEQYIKWPWYIWLGFIAGLIAI 1227
Bat_SL_CoV_Rs4084/1-1256                          EIDRL--------------NEVAKNLNESLIDLQELGKYEQYIKWPWYVWLGFIAGLIAI 1210
Bat_SL_CoV_RsSHC014/1-1256                        EIDRL--------------NEVAKNLNESLIDLQELGKYEQYIKWPWYVWLGFIAGLIAI 1210
Bat_SL_CoV_Rs7327/1-1256                          EIDRL--------------NEVAKNLNESLIDLQELGKYEQYIKWPWYVWLGFIAGLIAI 1210
Bat_SL_CoV_Rs9401/1-1256                          EIDRL--------------NEVAKNLNESLIDLQELGKYEQYIKWPWYVWLGFIAGLIAI 1210
Bat_SL_CoV_WIV1/1-1256                            EIDRL--------------NEVAKNLNESLIDLQELGKYEQYIKWPWYVWLGFIAGLIAI 1210
Bat_SL_CoV_Rs3367/1-1256                          EINRL--------------NEVAKNLNESLIDLQELGKYEQYIKWPWYVWLGFIAGLIAI 1210
Bat_SL_CoV_Rs4231/1-1255                          EIDRL--------------NEVAKNLNESLIDLQELGKYEQYIKWPWYVWLGFIAGLIAI 1209
```

```
Bat_SL_CoV_Rs4874/1-1255                          EIDRL--------------NEVAKNLNESLIDLQELGKYEQYIKWPWYVWLGFIAGLIAI 1209
SL_CoV_WIV16/1-1255                               EIDRL--------------NEVAKNLNESLIDLQELGKYEQYIKWPWYVWLGFIAGLIAI 1209
SARS_CoV_civet010/1-1255                          EIDRL--------------NEVAKNLNESLIDLQELGKYEQYIKWPWYVWLGFIAGLIAI 1209
SARS_CoV_Tor2/1-1255                              EIDRL--------------NEVAKNLNESLIDLQELGKYEQYIKWPWYVWLGFIAGLIAI 1209
Bat_SL_CoV__Rf4092/1-1234                         EIDRL--------------NEVAKNLNESLIDLQELGKYEQYIKWPWYVWLGFIAGLIAI 1188
BtRf_CoV_JL2012/1-1236                            EIDRL--------------NEVAKNLNESLIDLQELGKYEQYIKWPWYVWLGFIAGLVGL 1190
BtRs_YN2013/1-1233                                EIDRL--------------NEVAKNLNESLIDLQELGKYEQYIKWPWYVWLGFIAGLIAI 1187
Bat_CoV_Rp/Shaanxi2011/1-1240                     EIDRL--------------NEVAKNLNESLIDLQELGKYEQYIKWPWYVWLGFIAGLIAI 1194
Bat_SARS_CoV_Rf1/2004/1-1241                      EIDRL--------------NEVAKNLNESLIDLQELGKYEQYIKWPWYVWLGFIAGLVGL 1195
Bat_CoV/Yunnan2011/1-1241                         EIDRL--------------NEVAKNLNESLIDLQELGKYEQYIKWPWYVWLGFIAGLIAI 1195
SARS_CoV_Rs_672/2006/1-1241                       EIDRL--------------NEVAKNLNESLIDLQELGKYEQYIKWPWYVWLGFIAGLIAI 1195
Bat_SL_CoV_Rs4081/1-1241                          EIDRL--------------NEVAKNLNESLIDLQELGKYEQYIKWPWYVWLGFIAGLIAI 1195
Bat_SL_CoV_Rs4255/1-1241                          EIDRL--------------NEVAKNLNESLIDLQELGKYEQYIKWPWYVWLGFIAGLIAI 1195
Bat_SARS_CoV_Rm1/2004/1-1241                      EIDRL--------------NEVAKNLNESLIDLQELGKYEQYIKWPWYVWLGFIAGLIAI 1195
Bat_SARS_CoV_HKU3-1/1-1242                        EIDRL--------------NEVAKNLNESLIDLQELGKYEQYIKWPWYVWLGFIAGLIAI 1196
Bat_SARS_CoV_Rp3/1-1241                           EIDRL--------------NEVAKNLNESLIDLQELGKYEQYIKWPWYVWLGFIAGLIAI 1195
Bat_SL_CoV_As6526/1-1241                          EIDRL--------------NEVAKNLNESLIDLQELGKYEQYIKWPWYVWLGFIAGLIAI 1195
Bat_SL_CoV_Rs4237/1-1241                          EIDRL--------------NEVAKNLNESLIDLQELGKYEQYIKWPWYVWLGFIAGLIAI 1195
Bat_SL_CoV_Rs4247/1-1242                          EIDRL--------------NEVAKNLNESLIDLQELGKYEQYIKWPWYVWLGFIAGLIAI 1196
                                                  *:  :                    :.   :::: :     : ** *: *: :


HCoV_NL63/1-1356                                  LSLLVFCCLSTGCCGCCNCLTSSMRGCCDCGSTKL--PYYEFEKVHVQ-- 1356
Felis_Catus_CoV_NDL/UU88/1-1467                   IPLLLFCCLSTGCCGCFGCLGTCCHSLC---SRRQFESYEPIEKVHIH-- 1467
Ferret_CoV/1-1438                                 LPMLLFCCLSTGCCGCCGCLTSCLAGCCKNSCKR-PSYYEPMEKVHIN-- 1438
Canine_CoV/1-1457                                 IPLLLFCCCSTGCCGCIGCLGSCCHSIC---SRRQFENYEPIEKVHVH-- 1457
Mink_CoV_WD1133/1-1429                            LPMLLFCCLSTGCCGCCGCLSSCAAGCCKYGCSRDLSRYEPIEKVHVN-- 1429
Mink_CoV_1/1-1439                                 LPMLLFCCLSTGCCGCCGCLSSCAAGCCKYSCSRDLSRYEPIEKVHVN-- 1439
Mink_CoV_WD1127/1-1438                            LPMLLFCCLSTGCCGCCGCLSSCAAGCCKYSCSRDLSRYEPIEKVHVN-- 1438
Rhinolophus_affinis_bat_CoV_HKU-2_related/1-1119  AGLMLWCCLATGCCGMCGCLAATCSSCCDCRGTKLQ--SYEIEKIHVQ-- 1119
Rhinolophus_bat_CoV_HKU2/1-1128                   AGLMLWCCLATGCCGMCGCLAATCASCCDCRGTKLQ--SYEIEKIHIQ-- 1128
HCoV_OC43/1-1361                                  LVLLFFICCCTGCGTSCF---KKCGGCCDDYT-GYQ--ELVIKTSHDD-- 1361
HCoV_HKU1/1-1356                                  LVLLFFICCCTGCGSACF---SKCHNCCDEYG-GHH--DFVIKTSHDD-- 1356
MERS_CoV/1-1353                                   ALCVFFILCCTGCGTNCMG-KLKCNRCCDRYEEYDL--EPHKVHVH---- 1353
Bat_SL_CoV_ZC45/1-1246                            VMVTILLCCMTSCCSCLKG-CCSCGSCCK-FDEDDS--EPVLKGVKLHYT 1246
Bat_SL_CoV_ZXC21/1-1245                           VMVTILLCCMTSCCSCLKG-CCSCGFCCK-FDEDDS--EPVLKGVKLHYT 1245
Pangolin_CoV_MP789/1-1265                         IMVTIMLCCMTSCCSCLKG-CCSCGSCCK-FDEDDS--EPVLKGVKLHYT 1265
Bat_CoV_RaTG13/1-1269                             IMVTIMLCCMTSCCSCLKG-CCSCGSCCK-FDEDDS--EPVLKGVKLHYT 1269
SARS-CoV2/1-1273                                  VMVTIMLCCMTSCCSCLKG-CCSCGSCCK-FDEDDS--EPVLKGVKLHYT 1273
Bat_SL_CoV_Rs4084/1-1256                          VMVTILLCCMTSCCSCLKG-ACSCGSCCK-FDEDDS--EPVLKGVKLHYT 1256
Bat_SL_CoV_RsSHC014/1-1256                        VMVTILLCCMTSCCSCLKG-ACSCGSCCK-FDEDDS--EPVLKGVKLHYT 1256
```

```
Bat_SL_CoV_Rs7327/1-1256            VMVTILLCCMTSCCSCLKG-ACSCGSCCK-FDEDDS--EPVLKGVKLHYT 1256
Bat_SL_CoV_Rs9401/1-1256            VMVTILLCCMTSCCSCLKG-ACSCGSCCK-FDEDDP--EPVLKGVKLHYT 1256
Bat_SL_CoV_WIV1/1-1256              VMVTILLCCMTSCCSCLKG-ACSCGSCCK-FDEDDS--EPVLKGVKLHYT 1256
Bat_SL_CoV_Rs3367/1-1256            VMVTILLCCMTSCCSCLKG-ACSCGSCCK-FDEDDS--EPVLKGVKLHYT 1256
Bat_SL_CoV_Rs4231/1-1255            VMVTILLCCMTSCCSCLKG-ACSCGSCCK-FDEDDS--EPVLKGVKLHYT 1255
Bat_SL_CoV_Rs4874/1-1255            VMVTILLCCMTSCCSCLKG-ACSCGSCCK-FDEDDS--EPVLKGVKLHYT 1255
SL_CoV_WIV16/1-1255                 VMVTILLCCMTSCCSCLKG-ACSCGSCCK-FDEDDS--EPVLKGVKLHYT 1255
SARS_CoV_civet010/1-1255            VMVTILLCCMTSCCSCLKG-ACSCGSCCK-FDEDDS--EPVLKGVKLHYT 1255
SARS_CoV_Tor2/1-1255                VMVTILLCCMTSCCSCLKG-ACSCGSCCK-FDEDDS--EPVLKGVKLHYT 1255
Bat_SL_CoV__Rf4092/1-1234           VMATILLCCMTSCCSCLKG-ACSCGSCCK-FDEDDS--EPVLKGVKLHYT 1234
BtRf_CoV_JL2012/1-1236              FMAIILLCYFTSCCSCCKG-MCSCGSCCR-FDEDDS--EPVLKGVKLHYT 1236
BtRs_YN2013/1-1233                  VMATILLCCMTSCCSCLKG-ACSCGSCCK-FDEDDS--EPVLKGVKLHYT 1233
Bat_CoV_Rp/Shaanxi2011/1-1240       VMVTILLCCMTSCCSCLKG-ACSCGSCCK-FDEDDS--EPVLKGVKLHYT 1240
Bat_SARS_CoV_Rf1/2004/1-1241        FMAIILLCYFTSCCSCCKG-MCSCGSCCR-FDEDDS--EPVLKGVKLHYT 1241
Bat_CoV/Yunnan2011/1-1241           VMATILLCCMTSCCSCLKG-ACSCGSCCK-FDEDDS--EPVLKGVKLHYT 1241
SARS_CoV_Rs_672/2006/1-1241         VMATILLCCMTSCCSCLKG-ACSCGSCCK-FDEDDS--EPVLKGVKLHYT 1241
Bat_SL_CoV_Rs4081/1-1241            VMATILLCCMTSCCSCLKG-ACSCGSCCK-FDEDDS--EPVLKGVKLHYT 1241
Bat_SL_CoV_Rs4255/1-1241            VMATILLCCMTSCCSCLKG-ACSCGSCCK-FDEDDS--EPVLKGVKLHYT 1241
Bat_SARS_CoV_Rm1/2004/1-1241        VMVTILLCCMTSCCSCLKG-ACSCGSCCK-FDEDDS--EPVLKGVKLHYT 1241
Bat_SARS_CoV_HKU3-1/1-1242          VMVTILLCCMTSCCSCLKG-ACSCGSCCK-FDEDDS--EPVLKGVKLHYT 1242
Bat_SARS_CoV_Rp3/1-1241             VMVTILLCCMTSCCSCLKG-ACSCGSCCK-FDEDDS--EPVLKGVKLHYT 1241
Bat_SL_CoV_As6526/1-1241            VMATILLCCMTSCCSCLKG-ACSCGSCCK-FDEDDS--EPVLKGVKLHYT 1241
Bat_SL_CoV_Rs4237/1-1241            VMATILLCCMTSCCSCLKG-ACSCGSCCK-FDEDDS--EPVLKGVKLHYT 1241
Bat_SL_CoV_Rs4247/1-1242            VMATILLCCMTSCCSCLKG-ACSCGSCCK-FDEDDS--EPVLKGVKLHYT 1242
                                        .     *.*              *                 :
```
